# Replacing the *Z*-phenyl Ring in Tamoxifen^®^ with a *para*-Connected NCN Pincer-Pt-Cl Grouping by Post-Modification †

**DOI:** 10.3390/molecules26071888

**Published:** 2021-03-26

**Authors:** Guido D. Batema, Ties J. Korstanje, Gabriela Guillena, Gema Rodríguez, Martin Lutz, Gerard P. M. van Klink, Robert A. Gossage, Gerard van Koten

**Affiliations:** 1 Organic Chemistry and Catalysis, Faculty of Science, Utrecht University, Universiteitsweg 99, 3584 CG Utrecht, The Netherlands; guidobatema@gmail.com (G.D.B.); ties@korstanje.org (T.J.K.); gabriela.guillena@ua.es (G.G.); gemalongarela@hotmail.com (G.R.); gpmvanklink@gmail.com (G.P.M.v.K.); 2 Structural Biochemistry, Faculty of Science, Utrecht University, Padualaan 8, 3584 CH Utrecht, The Netherlands; m.lutz@uu.nl; 3 Department of Chemistry & Biology, Ryerson University, 350 Victoria Street, Toronto, ON M5B 2K3, Canada; gossage@ryerson.ca

**Keywords:** post-modification of NCN-pincer platinum halide, McMurry reaction on organometallics, NCN-pincer platinum Tamoxifen^®^-type derivatives

## Abstract

Post-modification of a series of NCN-pincer platinum(II) complexes [PtX(NCN-R-4)] (NCN = [C_6_H_2_(CH_2_NMe_2_)_2_-2,6]^–^, R = C(O)H, C(O)Me and C(O)Et), X = Cl^–^ or Br^–^) at the *para*-position using the McMurry reaction was studied. The synthetic route towards two new [PtCl(NCN-R-4)] (R = C(O)Me and C(O)Et) complexes used above is likewise described. The utility and limitations of the McMurry reaction involving these pincer complexes was systematically evaluated. The predicted “homo-coupling” reaction of [PtBr(NCN-C(O)H-4)] led to the unexpected formation of 3,3′,5,5′-tetra[(dimethylamino)methyl]-4,4′-bis(platinum halide)-benzophenone (halide = Br or Cl), referred to hereafter as the bispincer-benzophenone complex **13**. This material was further characterized using X-ray crystal structure determination. The applicability of the pincer complexes in the McMurry reaction is shown to open a route towards the synthesis of tamoxifen-type derivatives of which one phenyl ring of Tamoxifen^®^ itself is replaced by an NCN arylplatinum pincer fragment. The newly synthesized derivatives can be used as potential candidates in anti-cancer drug screening protocols. Two NCN-arylpincer platinum tamoxifen type derivatives, **5** and **6**, were successfully synthesized and of **5** the separation of the diastereomeric *E-/Z*-forms was achieved. Compound **6**, which is the pivaloyl protected NCN pincer platinum hydroxy-Tamoxifen^®^ derivative, was obtained as a mixture of *E-/Z-*isomers. The new derivatives were further analyzed and characterized with ^1^H-, ^13^C{^1^H}- and ^195^Pt{^1^H}-NMR, IR, exact mass MS and elemental analysis.

## 1. Introduction

The increasing interest in the application of organometallic complexes in functional materials, for example, in biocatalysis with artificial metalloenzymes [1], in natural enzymes [2], in organic light emitting diodes (OLEDs) [3], in polymers [4], and organometallic pharmaceuticals [5,6,7], has stimulated the development of metal complexes with an increased stability towards air, moisture and high thermal stress. Due to their increased stability, these complexes can be modified or used as a reactant in a chemical reaction without decomposing. The ferrocenes are a good example of organometallic complexes used in compounds with anticancer activity [8]. Another class of stable organometallic compounds that is pertinent to many of the research projects in the career of Dr. Michel Pfeffer, are those that emerged from his pioneering research in the field of cyclometallated organometallic compounds [9]. Starting with the synthesis of the first cyclopalladated [9,10] and ruthenated [11,12] complexes, Pfeffer later extended his interests beyond the synthesis aspects while entering into multidisciplinary collaborations studying the biochemical aspects of his ruthenated compounds [13,14,15,16]. In his cyclometallation research, he focused merely on the use of nitrogen donor atom containing directing groups, e.g., amines, imines, or 2-pyridyl groupings, leading to C,N-cyclometallated aryl, alkenyl and alkyl organometallics. In our longstanding collaborations, we used directed *ortho*-lithiation [17,18] followed by transmetallation as a useful, alternate approach when direct *ortho*-metallation through C-H activation using directing nitrogen donor groupings as a synthetic route appeared not to be feasible [19].

The latter approach was extensively explored for the synthesis of bis(*ortho*-chelated) NCN-aryl-pincer platinum complexes, [PtX(NCN-R-4)] (NCN = [4-RC_6_H_2_(CH_2_NMe_2_)_2_-2,6]^–^; (X = Cl, Br, I) [20], by the Amsterdam and Utrecht research groups. Based on their extraordinary, broad stability towards air, moisture and higher temperatures, these complexes were studied for their properties as, for instance, color biomarkers, a detection label, and as a luminescent material [21]. We also showed the feasibility of post-modification of the NCN backbone in the [MX(NCN-R-4)] pincer system (M = Ni, Pd or Pt; X = Cl, Br or I) with a large library of different *para*-substituents R (R = –NO_2_, –C(O)Me, –COOH, –CHO, –SO_3_H, –PO(OEt)_2_, –PO(OH)(OEt), –PO(OH)_2_, –I, –Cl, –H, –CH_2_OH, –OMe, –SMe or –NH_2_) [22]. In some cases, these substituents could even be directly used as a reactant in subsequent synthetic reaction protocols [23,24,25].

**Chart 1:**
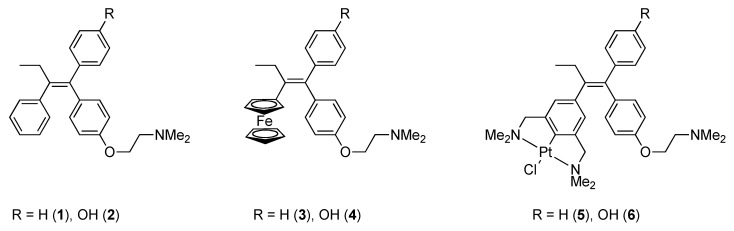
Tamoxifen^®^ (**1** and **2**), ferrocifens (**3** and **4**) and its NCN-pincer Pt(II) analogues, named pincercifen (**5** and **6**).

To further explore the scope of the *para* post-modification of NCN-pincer metal complexes, we were prompted by the extensive work of Jaouen and coworkers on the development of both ferrocifen (**3**) and hydroxyferrocifen (**4**) [8,26]. In these organometallic derivatives of the anti-cancer drug Tamoxifen^®^ (**1**) [26,27], a phenyl ring of Tamoxifen^®^ is replaced by a ferrocene moiety (Chart 1) [26]. Introduction of this metallocene fragment resulted in an enhanced anti-proliferation effect in cancer cells.

So, on the one hand prompted by the question to explore the scope of the *para* post-modification of NCN pincer-metal complexes, and on the other to explore the effect of the replacement of the ferrocenyl group by an NCN pincer-metal halide one, we set out to develop a synthetic methodology in order to synthesize NCN-pincer platinum halide containing tamoxifen-type derivatives. In this manuscript, we report our first results and findings on the synthesis of these molecules.

## 2. Results

### 2.1. Synthesis

**Scheme 1:**
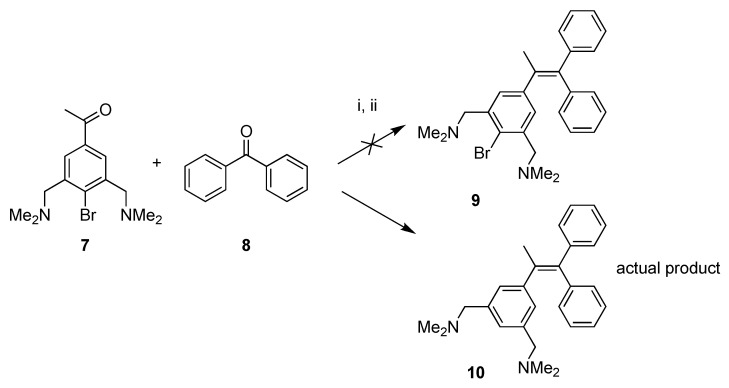
McMurry reaction of **7** and **8** yielding de-halogenated product **10**. *Reagents and conditions*: (i) TiCl_4_(THF)_2_, Zn(0), THF, reflux, 16 h; (ii) H_2_O, K_2_CO_3_.

In general, the Tamoxifen^®^ and ferrocifen derivatives are synthesized using the McMurry coupling reaction of ketones [26,28], using low valent titanium as a coupling intermediate. In this study, we will also use this methodology in order to synthesize the NCN-pincer platinum derivatives **5** and **6**. To test the applicability of pincer ligands in the McMurry reaction, the acetophenone pincer ligand **7** was synthesized by a known procedure [22]. We thus attempted to couple **7** with benzophenone **8**, under typical McMurry conditions. Surprisingly, the product mixture obtained after purification, did not contain desired product **9**, but rather consisted mainly of the corresponding alkene derivative **10** (concluded via ^1^H-NMR spectroscopy). In this dehalogenated product, the bromide had been cleaved from the ligand backbone and was exchanged for a hydrogen atom. Apparently, under the applied McMurry reaction conditions (refluxing in THF for 16 h in the presence of Zn and Ti), the phenyl bromide **7** or **9** is effectively hydrolyzed (Scheme 1). From the literature, it is known that the McMurry reaction should be compatible with organohalides [28]. For example, it has been shown that 4-bromoacetophenone can be used in the McMurry reaction, forming the homocoupled product, without affecting the phenyl bromide bond [29]. A possible explanation for the dehalogenation of **7** or **9** can be the intermediate formation of an NCN-Ti(IV) complex [30]. These complexes are known to be highly sensitive to air and moisture, and readily decompose at higher temperatures thereby forming the resulting protonated NCN ligand. It was therefore decided to use metallated building blocks directly as a reagent in the McMurry reaction in order to avoid this problem.

**Scheme 2:**
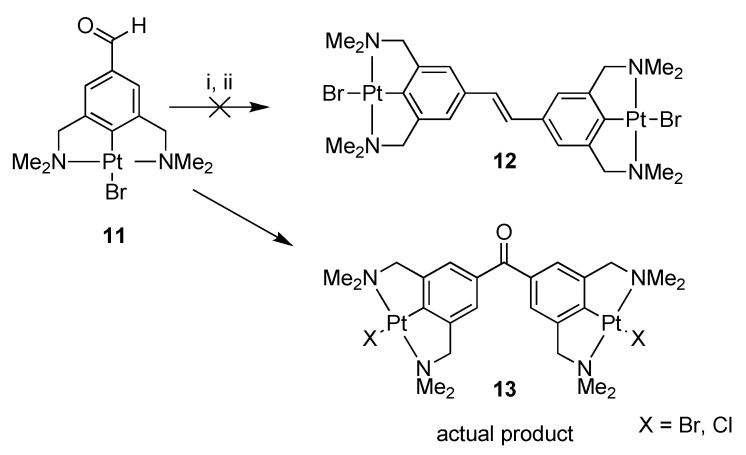
*Reagents and conditions*: (i) TiCl_4_(THF)_2_, Zn(0), THF, reflux, 16 h; (ii) H_2_O, K_2_CO_3_.

**Figure 1:**
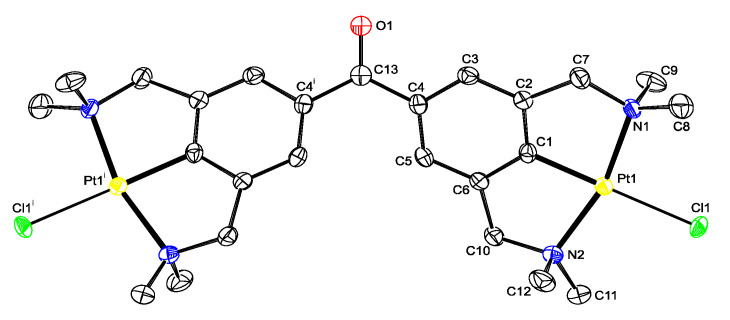
Displacement ellipsoid plot of **13**, drawn at the 50% probability level. Hydrogen atoms and disordered solvent molecules are omitted for clarity. The halogen position is occupied by 60% chlorine and 40% bromine; only chlorine derivative is shown. Symmetry operation i: −x, y, 0.5-z.

Platinum pincer aldehyde [PtBr(NCN-CHO-4)] **11**, a complex developed in a previous study [22,24], was used in a homocoupling reaction under McMurry conditions, in order to synthesize the bisplatinum-stilbene compound **12** (Scheme 2). After workup, the isolated product consisted of a mixture of compounds, also containing the starting aldehyde **11**. In attempts to separate the product mixture into separate components, a yellow crystalline solid was obtained after crystallization of the mixture from toluene solution. The structure in the solid state was determined with X-ray crystallography, and, surprisingly, confirmed the crystals to be of the bimetallic benzophenone platinum pincer **13**; i.e., rather than **12** the coupling product **13** was obtained in which the two platinum pincer fragments are connected via a carbonyl linker. No signals of the stilbenoid product **12** were found in the residue. During the coupling reaction on the titanium surface, an as yet unclear reaction has occurred. From the crystal structure data (see Figure 1), it was also observed that there is substitutional disorder between bromine and chlorine on the halogen sites, the chlorido ligand likely having been delivered by TiCl_4_.

Similar halogen scrambling was observed in both the ^1^H- and ^195^Pt{^1^H}-NMR spectra, by the presence of two signals for the dimethylamino protons of the CH_2_NMe_2_ groups, and likewise, in the ^195^Pt-NMR spectrum two signals were found at −3107 and −3130 ppm resulting from the two different platinum halide species. From the mass spectrum, it became clear that for **13** only the X = Br, Cl, and X = Cl species were present, whereas no mass peak for the X = Br only was found (see Figure S1). To avoid halogen scrambling, it was decided to use only PtCl complexes in the rest of our study.

**Scheme 3:**
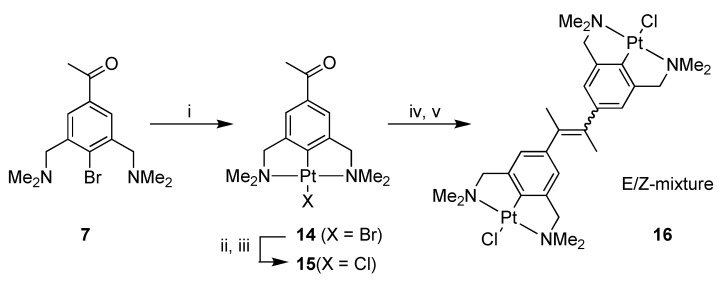
Synthesis of **14**, **15** and **16**. *Reagents and Conditions:* (i) [Pt(4-Tol)_2_(SEt_2_)]_2_, benzene, reflux, 3 h, 92%; (ii) AgOTf, acetone, rt, 30 min; (iii) NaCl, rt, 1 h, 82%; (iv) TiCl_4_(THF)_2_, Zn(0), THF, reflux, 16 h; (v) H_2_O, K_2_CO_3_.

Platinum pincer acetophenone **14** was prepared by platination of 4-bromo-3,5-bis[(dimethylamino)methyl]acetophenone (**7**) [22,24] with Canty’s reagent, [Pt(4-Tol)_2_(SEt_2_)]_2_ [31]. The PtBr complex **14** was subsequently converted into the PtCl complex **15** by the use of silver salts (AgOTf), and subsequent addition of NaCl (Scheme 3).

Platinum pincer acetophenone **15** was used in the McMurry reaction, in order to achieve the homocoupled platinum alkene **16** (Scheme 3). The reaction resulted in the isolation of a product mixture consisting of an equimolar amount of the corresponding *E-* and *Z*-isomers of **16** combined with unreacted **15**. The formation of **16** showed that it was possible to use the platinum complex as a reactant in a McMurry reaction. Attempts to remove **15** from the product mixture by crystallization were, unfortunately, not successful.

**Scheme 4:**
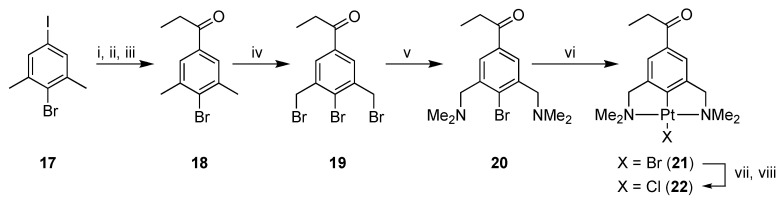
Reagents and Conditions: (i) *n*-BuLi, THF, −78 °C; (ii) *N,N*-dimethylpropionamide, −78 °C → rt; (iii) H_2_O, 43%; (iv) NBS, AIBN, MeOAc, 12 h, h*ν*, 49%; (v) HNMe_2_, Et_2_O, 12 h, rt, 94%; (vi) [Pt(4-Tol)_2_(SEt_2_)]_2_, toluene, 70 °C, 3 h, 96%; (vii) AgBF_4_, acetone, rt; (viii) NaCl, 96%.

Proceeding on our quest to synthesize **5** and **6**, platinum-containing ketone **22** was synthesized starting from 4-iodo-1-bromo-2,6-dimethylbenzene (**17**) [22,24] via a multistep reaction procedure (Scheme 4). In the first step, the iodo group of **17** was selectively substituted with lithium by a metallation reaction with *n*-BuLi at low temperature. The lithiated species was reacted in situ with *N,N*-dimethylpropionamide and after hydrolysis 4-bromo-3,5-dimethylphenyl ethyl ketone **18** was obtained, which was converted to bis-benzyl bromide **19** via a radical bromination reaction [32].

**Scheme 5:**
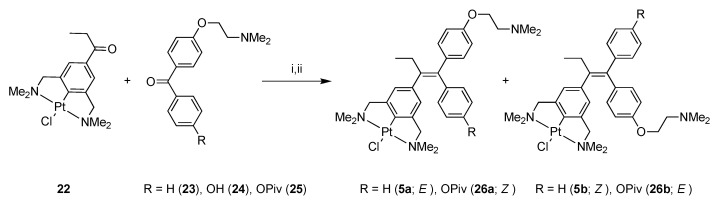
Synthesis of *E/Z* complexes **5** and **26** via McMurry coupling. *Reagents and Conditions*: (i) TiCl_4_(THF)_2_, Zn(0), THF, reflux, 16h; (ii) H_2_O, K_2_CO_3_ (for **5** 36%).

Compound **19** was then converted into 2,6-bis(dimethylamino)benzyl bromide **20** by an amination reaction using excess dimethylamine. Propiophenone pincer ligand **20** was selectively platinated with Canty’s reagent, [Pt(4-Tol)_2_(SEt_2_)]_2_, affording platinum pincer complex **21** in a good overall yield (19% starting from **17**). Complex **21** was then converted to PtCl complex **22** via a halogen exchange reaction using AgBF_4_ and NaCl, subsequently, as described earlier. The organic benzophenone fragment **23** was synthesized according to a literature procedure [33] (Scheme 5).

**Figure 2:**
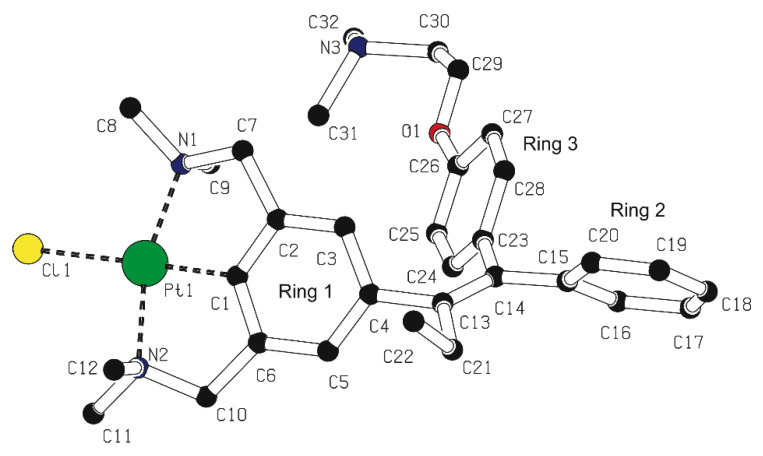
PLUTON visualization of **5b** in the crystal [34]. Hydrogen atoms are omitted for clarity.

Finally, the ketones **22** and **23** were coupled, using the McMurry protocol, giving a mixture of products containing the *E*/*Z* mixture **5a** and **5b**, respectively, and the homo-coupled products derived from **23**. The inorganic compounds were partly separated from the organics by precipitation from CH_2_Cl_2_, with pentane, and the inorganics were isolated after centrifugation; this resulted in the isolation of an *E*/*Z* mixture **5a** and **5b** (3:7). However, this material still contained some impurities. Fortunately, the isomers could be separated by silica gel column chromatography, although mainly yielding fractions containing different ratios of both isomers but also some fractions containing the separated isomers in pure form. The isomers **5a** and **5b** were characterized with ^1^H-, ^13^C{^1^H}-, and ^195^Pt{^1^H}-NMR, IR, exact mass MS and elemental analysis. For the *Z*-isomer **5b**, an X-ray crystal structure [34] was obtained (see Figure 2 below).

With the successful synthesis of **5** in mind, we tried to synthesize **6**, which is an analogue of both **2** and **4**, i.e., the hydroxy analogues of Tamoxifen^®^ and ferrocifen. These compounds are also active metabolites when tested for anti-cancer activity.

**Scheme 6:**
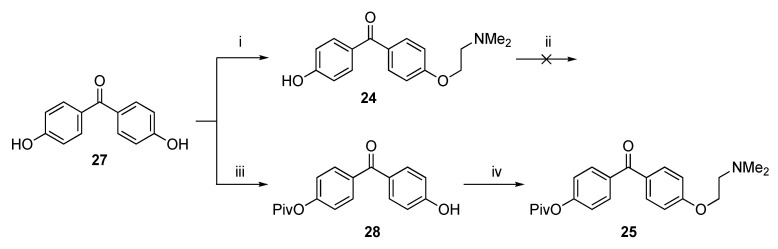
Functionalization of 4,4′-hydroxybenzophenone **27**. Reagents and Conditions: (i) ClCH_2_CH_2_NMe_2_·HCl, K_2_CO_3_, acetone, reflux, 20 h, 21%.; (ii) TBDMS-Cl, imidazole or NaH, THF; (iii) NaH, PivOCl, THF; (iv) ClCH_2_CH_2_NMe_2_·HCl, K_2_CO_3_, acetone, reflux, 20 h, 20%.

In order to be able to prepare **6**, we first functionalized 4,4′-dihydroxybenzophenone **27** with a single 2-(dimethylamino)ethoxy group, obtaining **24** according to a modified literature procedure [35]. Repeated attempts to couple **22** and **24** in order to obtain **6′** i.e., the precursor of **6** (as *E-*/*Z-*mixture) failed, and only the starting metal complex **22** was recovered from the reaction mixtures. Presumably, the free phenol group in **24** hampers the coupling reaction. Therefore, it was decided to protect the phenol group of **24** with a *tert*-butyldimethylsilyl (TBDMS) group (Scheme 6). When **24** was reacted with TBDMS-Cl using imidazole as a base, no reaction took place to form the desired product. The use of NaH was likewise not successful. It was decided to prepare the single protected pivaloyl (OPiv) hydroxybenzophenone **28** instead using a literature procedure [35]. Next, **28** was functionalized with a dimethylaminoethoxy group offering **25**.

The doubly functionalized benzophenone **25** was then successfully coupled with **22** via the McMurry reaction, yielding a product mixture after workup. Purification by silica gel chromatography gave **26** as a mixture of *E*- and *Z*-isomers (62% yield). In a second run, attempts were made to separate the *E*/*Z* isomers using silica gel chromatography, yielding a fraction in which one of the isomers of **26** was present in a 49:1 ratio, see Experimental section. Regrettably, synthetic attempts to de-protect **26** in order to obtain **6**, were unsuccessful up to this time. Synthetic methodologies are currently under development to address this issue.

### 2.2. Structural Features of ***13***, and ***5b*** in the Solid State

Suitable crystals for single crystal X-ray structure determination for **13** were obtained by slow evaporation of a toluene solution containing the product mixture from the reaction depicted in Scheme 2 (Figure 1). In the crystals the molecule is positioned on an exact, crystallographic two-fold axis. For **13**, the overall geometric conformation is similar to that found for other benzophenone solid state structures [28]. An interplanar angle of 23.47(13)° is observed between the plane of the phenyl ring and the plane containing the C=O fragment and the C4 and C4^i^ atoms. The non-planarity of the molecule results from the minimization of the intramolecular interactions between the two rings. The length of the C=O bond of 1.237(5) Å is in the expected bond length order of 1.22 Å.

Crystals of **5b** were obtained by slow evaporation of a toluene/ethanol/dichloromethane mixture containing the compound. For **5b**, the overall conformation of the structure is similar to that of other tamoxifen-based structures in the solid state (Figure 2) [26,36]. The triphenylethylene system containing the platinum pincer fragment (ring 1), the unsubstituted phenyl (ring 2) and the 4-[2-(dimethylamino)ethoxy]phenyl (ring 3), adopts a propeller conformation.

In **5b**, angles of 74.9(7), 48.6(7), and 88.4(6)° are found between the planes of ring 1 and ring 2, ring 1 and ring 3, ring 2 and ring 3, respectively. A dihedral angle of 52.5(7)° is found between ring 1 and the plane formed by C4, C13, C14, C21. Angles of 48.8(9) and 56.9(9)° are found for ring 2 and ring 3, respectively, with the plane formed by C13, C14, C15, and C23. The phenyl rings are not coplanar with the ethylene bond C13-C14, and an angle of 6.4(9)° is found between the planes formed by C4, C13, C14, C21, and C13, C14, C15, C23, which both contain the ethylene fragment.

Angles for C4-C13-C21 and C15-C14-C23 of 113.7(12) and 117.0(11)°, respectively, are found, which are shorter than the angle of 120° expected to be found for formal sp^2^ hybridization, but which is commonly found in tamoxifen analogs [20,37].

Comparison of the molecular structures of **5b** and **13** with earlier reported [PtX(NCN)] (X = Br or Cl) pincer complexes (see the various *mer*-NCN pincer-metal complexes reported in refs 20 and 37) show a similar distorted square planar geometry around the Pt nucleus, in which the carbon atoms of the benzylic arms (C7 and C10) are positioned above and below the plane defined by C1-N1-Pt1-N2-Cl1. For the two five-membered metallacycles, which are slightly puckered, torsion angles for the Pt1-N1-C7-C2 and Pt1-N2-C10-C6 bonds amounting to −29.0(3)° to −29.2(2)° were found in **13**. The Pt-C distances of **5b**, and **13** are as expected [20,37].

## 3. Discussion

**Figure 3:**
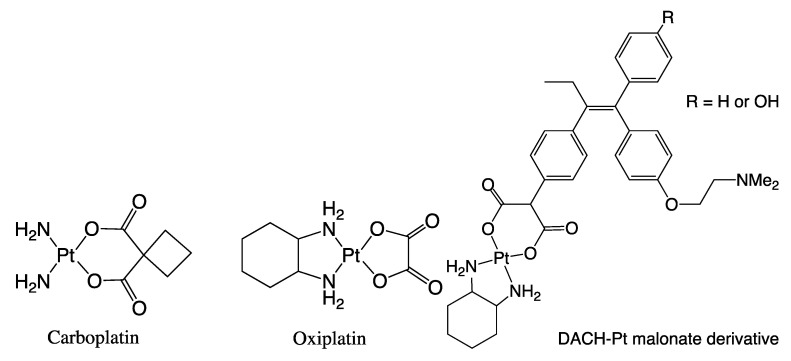
Example of a coordination compound containing Pt(II) used in the screening for anti-cancer activity.

The success of combining an organometallic complex with a selective estrogen receptor modulator (SERM) [38] is clearly demonstrated by the creative and extensive work of Jaouen and coworkers on ferrocifen (**3**) and hydroxyferrocifen (**4**) (Figure 3) [8,25,39,40,41,42]. In these organometallic derivatives of the anticancer drug Tamoxifen^®^ (**1**) [43], a phenyl ring of the original Tamoxifen^®^ is replaced by a ferrocenyl moiety. The fixation of a ferrocenyl group onto an anti-estrogen vector hydroxytamoxifen (**2**), provided hydroxyferrocifen (**4**) which showed an anti-estrogenic effect and also cytotoxic activity [44]. The cytotoxic activity of the ferrocifen derivatives is ascribed to the redox properties of the ferrocene moiety [45]. The anti-estrogenic effect of this compound was ascribed to a conformational change of the α-receptor site of the estrogen receptor containing protein, present in the tumor cell, upon binding of hydroxy-ferrocifen [44,46]. Unfortunately, the ferrocifens show a reduced relative binding affinity for the estrogen receptor, compared to that of hydroxy-Tamoxifen^®^, due to the steric size of the ferrocenyl group [39].

The present result shows that the use of the McMurry reaction for post-modification of the *para* aryl-ring position of parent NCN-pincer platinum is possible [22,37]. This reaction protocol provides a direct, short synthetic route to the platinum(II) containing tamoxifen derivatives **5** and **6** (abbreviated as pincercifens, cf. Figure 3). To accomplish this, new NCN platinum pincer acetophenone and propiophenone building blocks were developed. Both the applications and limitations of the McMurry coupling protocol were established using the free NCN ligands and their platinum complexes as starting compounds.

Similar to ferrocifen, the pincercifens **5** and **6** contain a redox active metal center, but like the ferrocenyl fragment in ferrocifen, the NCN-pincer platinum fragment is geometrically different from that of the *Z*-phenyl group, which is present in Tamoxifen^®^, cf. the structures of these compounds in the solid state, see Supporting Materials, 5 [42,47]. Both properties make the pincercifens potential interesting candidates for screening on anticancer activity.

Based on the bonding motifs present in the well-known and clinically used Pt-based antitumor drugs [48,49] such as cisplatin (i.e., *cis*-[PtCl_2_(NH_3_)_2_]) [50,51] and other platinum containing complexes (Carboplatin^®^ and Oxiplatin^®^: Figure 3), the Jaouen group also synthesized and screened diaminocyclohexane-malonate-platinum(II) derivatives of Tamoxifen^®^: DACH-Pt (Figure 3) [27]. In contrast to the stability of the organometallic fragments in ferrocifen and pincercifen, the lability of this type of platinum complexes shown in Figure 3 under physiological conditions is sometimes used to release and deliver an active metal center close to the target site [27]. However, this lability can also be problematic, especially when the metal center is released before reaching the receptor or target site [5]. In the case of ferrocifen and pincercifen, it is the stability of the respective organometallic groupings that can be an advantage, because it is shown for both complexes that the covalently bound metal center is compatible under a diversity of reaction conditions without the cleavage of the metal-C sigma bond [24].

In the mode of action of some Pt-based antitumor drugs, a cationic [PtCl(H_2_O)(L)_2_]^+^ species (L = coordinating ligand) that is formed in the cell by aquation of the [PtCl_2_(L)_2_] complex, binds to DNA [51,52]. Here, the pincercifens offer potentially interesting features. For an NCN-PtCl complex, it was found that the Pt-Cl grouping is able to form intermolecular non-covalent hydrogen bonds, for example, with a phenol group, i.e., forming O–H•••Cl–Pt bonds [53]. Furthermore, it was shown that [Pt(NCN)(H_2_O)]^+^ cations, either as such or as irreversibly embedded by covalent bonding in lipases, under chloride poor reaction conditions, strongly bind to, for example, pyridyl functionalized substrates [54,55]. Therefore, it is not unlikely that, under physiological conditions, an NCN-PtCl or an aquated NCN-Pt complex [55] will bind to DNA via coordination and/or hydrogen bonding, inducing cytotoxic activity.

## 4. Materials and Methods

### 4.1. Synthesis

#### 4.1.1. General Information

All reactions involving air- or moisture-sensitive reagents were performed by standard Schlenk techniques unless stated otherwise. Pentane, THF, benzene, toluene and Et_2_O were distilled from Na/benzophenone, CH_2_Cl_2_ was distilled from CaH_2_ prior to use. The platinum precursor [Pt(p-Tol)_2_(SEt_2_)]_2_ [36], [PtBr(NCN-CHO-4)] (**11**) [20], 4-bromo-3,5-bis(dimethylamino)methyl]acetophenone (**7**) [22], 3,5-dimethyl-4-bromo-iodobenze (**17**) [20], 4-[2-(dimethylamino)ethoxy]benzophenone (**23**) [25], 4-[2-(dimethylamino)ethoxy]-4′-hydroxybenzophenone (**24**) [54], and 4-trimethylacetoxy-4′-hydroxybenzophenone (**28**) [54] were synthesized according to literature procedures. All other reagents were commercially available and used without further purification. ^1^H- and ^13^C{^1^H}-NMR spectra were recorded at 25 °C on AC 300 NMR (Bruker) or Inova 300 or 400 spectrometers (Varian) (operating frequencies: for ^1^H spectra at 300 and 400 MHz; for ^13^C spectra at 75 and 101 MHz), chemical shifts are depicted in ppm and referenced to residual solvents resonances. ^195^Pt{^1^H}-NMR spectra were recorded on a Varian Inova 300 MHz NMR spectrometer (operating at 64.4 MHz), referenced to external Na_2_PtCl_6_ (1 M in D_2_O, δ = 0 ppm). Elemental analyses were performed by Kolbe, Mikroanalytisches Laboratorium (Mülheim a.d. Ruhr, Germany). ESI-MS spectra were obtained from the Biomolecular Mass Spectrometry Group at Utrecht University. Infrared spectra were recorded with a Spectrum 1 FT-IR spectrometer (Perkin Elmer).

##### 4-Bromo-3,5-bis[(dimethylamino)methyl]acetophenone (**7**)

IR (ATR): ῦ = 3074, 2976, 2948, 2858, 2822, 2772, 1683 (C=O), 1577, 1455, 1428, 1401, 1361, 1348, 1296, 1277, 1265, 1218, 1189, 1153, 1097, 1057, 1041, 1012, 983, 939, 912, 897, 856, 710, 685 cm^−1^ [15].

##### [PtBr(NCN-C(O)Me-4)] (**14**)

A mixture of 4-bromo-3,5-bis[(dimethylamino)methyl]acetophenone (**7**, 0.85 g, 2.71 mmol) and [Pt(Tol-4)_2_(SEt_2_)]_2_ (1.26 g, 1.35 mmol) in dry benzene (50 mL) was refluxed for 3 h. The slightly yellow solution was allowed to cool to RT, and all volatiles were removed in vacuo. The residue was dissolved in a minimal amount of CH_2_Cl_2_ and the product precipitated from the solution upon addition of pentane. Product **14** was isolated as a yellow powder (1.27 g, 2.50 mmol, 92%). ^1^H-NMR (400 MHz, CD_2_Cl_2_): δ = 7.45 (s, 2H; ArH), 4.07 (s, ^3^*J*(H,Pt) = 46.0 Hz, 4H; CH_2_N), 3.09 (s, ^3^*J*(H,Pt) = 38.4 Hz, 12H; CH_3_N), 2.48 (s, 3H; CH_3_C(O)); ^13^C{^1^H}-NMR (101 MHz, CD_2_Cl_2_): δ = 197.7 (C=O), 155.3 (C *ipso* to Pt), 143.9 (Ar*C*C(O)), 133.5 (Ar*C*CH_2_), 119.9 (^3^*J*(C,Pt) = 34.9 Hz; ArCH), 77.2 (^2^*J*(C,Pt) = 64.1 Hz; (CH_2_N), 55.0 (CH_3_N), 26.6 (*C*H_3_C(O)); IR (ATR): ῦ = 3008, 2982, 2920, 2832, 1663 (C=O), 1581, 1570, 1454, 1432, 1401, 1352, 1301, 1270, 1199, 1140, 1113, 1049, 1030, 1015, 972, 961, 943, 896, 887, 870, 836, 711, 672 cm^−1^; MS (ES+; CH_2_Cl_2_) *m/z*: 509.0188 [M + H]^+^; elemental analysis calcd (%) for C_14_H_21_BrN_2_OPt (508.31): C 33.08, H 4.16, N 5.51; found: C 33.23, H 4.20, N 5.42.

##### [PtCl(NCN-C(O)Me-4)] (**15**)

A mixture of **14** (0.29 g, 0.57 mmol) and silver triflate (AgOTf, 0.16 g, 0.62 mmol) in wet acetone (20 mL) was stirred for 30 min. The formed suspension was filtered over Celite and the filtrate was evaporated to dryness in vacuo. The residue was dissolved in wet acetone (20 mL) and an excess of NaCl was added. The resulting suspension was stirred for 1h after which the solvent was removed in vacuo. The residue was dissolved in CH_2_Cl_2_ and subsequently washed with water and brine. The organic layer was separated, dried over MgSO_4_, and filtered. The filtrate was evaporated to dryness and dissolved in a minimal amount of CH_2_Cl_2_. The product precipitated from the solution upon addition of pentane and afforded **15** as a pale white powder (0.22 g, 0.47 mmol, 82%). ^1^H-NMR (300 MHz, CD_2_Cl_2_): δ = 7.45 (s, 2H; ArH), 4.07 (s, ^3^*J*(H,Pt) = 46.2 Hz, 4H; CH_2_N), 3.05 (s, ^3^*J*(H,Pt) = 38.1 Hz, 12H; CH_3_N), 2.48 (s, 3H; CH_3_C(O)); ^13^C{^1^H}-NMR (75 MHz, CDCl_3_): δ = 197.7 (C=O), 154.2 (C *ipso* to Pt), 143.5 (Ar*C*C(O)), 133.2 (Ar*C*CH_2_), 119.7 (ArCH), 77.2 (NCH_2_), 54.3 (NCH_3_), 26.4 (*C*H_3_C(O)); ^195^Pt{^1^H}-NMR (64 MHz, CD_2_Cl_2_): δ = –3101; IR (ATR): ῦ = 3046, 3009, 2981, 2921, 1663 (C=O), 1580, 1568, 1456, 1433, 1397, 1355, 1300, 1274, 1233, 1197, 1141, 1115, 1049, 1017, 973, 961, 944, 896, 882, 873, 836, 712, 670 cm^−1^; MS (ES+; CH_2_Cl_2_) *m/z*: 464.0717 [M + H]^+^; elemental analysis calcd (%) for C_14_H_21_ClN_2_OPt (463.86): C 36.25, H 4.56, N 6.04; found: C 36.39, H 4.51, N 6.11.

##### 3,5-Dimethyl-4-bromopropiophenone (**18**)

To a solution of **17** (10.2 g, 32.8 mmol) in dry Et_2_O (250 mL) at −78 °C a solution of *n*-BuLi (1.6M in hexane, 22 mL, 35.2 mmol) was added dropwise, and the mixture was stirred at this temperature for 30 min. Next, *N,N*-dimethylpropionamide (9 mL, 82.8 mmol) was added dropwise and the reaction mixture was stirred at the same temperature for 1 h. The mixture was allowed to warm to rt, and the reaction was quenched by adding an aqeous HCl solution (1 M, 40 mL). The layers were separated and the water layer was extracted with Et_2_O (2 × 40 mL). The combined organic fractions were subsequently washed with an aqueous HCl solution (1 M, 2 × 40 mL), a saturated sodium bicarbonate solution (2 × 40 mL), and brine (40 mL). The organic layer was dried using MgSO_4_, and evaporated to dryness. The residual oil was crystallized from hexane to afford **18** as colorless crystals (3.39 g, 14.1 mmol, 43%). ^1^H-NMR (200 MHz, CDCl_3_): δ = 7.64 (s, 2H; ArH), 2.95 (q, ^3^*J*(H,H) = 7.3 Hz, 2H; CH_2_), 2.47 (s, 6H; ArCH_3_), 1.21 (t, ^3^*J*(H,H) = 7.3 Hz, 3H; CH_3_); ^13^C{^1^H}-NMR (75 MHz, CDCl_3_): δ = 200.5 (C=O), 138.9 (Ar*C*CH_2_), 135.4 (Ar*C*C(O)), 133.1 (ArCBr), 127.3 (ArCH), 32.0 (*C*H_2_C(O)), 24.2 (ArCH_3_), 8.4 (*C*H_3_CH_2_); IR (ATR): ῦ = 2988, 2941, 2907, 1674 (C=O), 1586, 1464, 1436, 1410, 1377, 1349, 1291, 1177, 1093, 1044, 1028, 1007, 978, 946, 891, 855, 801, 703 cm^−1^; MS (ES+; CH_2_Cl_2_) *m/z*: 481.0164 [2M + H]^+^, 240.9976 [M + H]^+^; elemental analysis calcd (%) for C_11_H_13_BrO (241.12): C 54.79, H 5.43; found: C 54.67, H 5.38.

##### 3,5-Bis(bromomethyl)-4-bromopropiophenone (**19**)

In a 100 mL one necked round-bottom flask, **18** (1.81 g, 7.5 mmol) was dissolved in MeOAc (40 mL). To the solution *N*-bromosuccinimide (3.18 g, 17.9 mmol) and 2,2’-azobisisobutyronitrile (0.08 g, 0.5 mmol) were added. After the connection of a reflux condenser, the stirred mixture was irradiated for 12 h with a lamp (80 W). The solvent was evaporated and the residue was dissolved in a mixture of CHCl_3_/hexane (1:9). The formed precipitate was filtered off and the filtrate was evaporated to dryness, and subsequently the residue was crystallized from a mixture of CHCl_3_/hexane (1:2) to obtain **19** as a white solid (1.48 g, 3.7 mmol, 49%). ^1^H-NMR (400 MHz, CDCl_3_): δ = 7.96 (s, 2H, ArH), 4.66 (s, 4H, CH_2_Br), 2.99 (q, ^3^*J*(H,H) = 7.2 Hz, 2H; C*H*_2_C(O)), 1.22 (t, ^3^*J*(H,H) = 7.2 Hz, 3H; C*H*_3_CH_2_); ^13^C{^1^H}-NMR (101 MHz, CDCl_3_): δ = 199.0 (C=O), 139.4 (Ar*C*CH_2_), 136.6 (Ar*C*C(O)), 131.7 (ArCBr), 130.5 (ArCH), 33.5 (CH_2_Br), 32.2 (*C*H_2_C(O)), 8.2 (*C*H_3_CH_2_); IR (ATR): ῦ = 3058, 2978, 2935, 2902, 2874, 1689 (C=O), 1586, 1461, 1438, 1409, 1375, 1350, 1291, 1259, 1213, 1185, 1120, 1091, 1040, 1022, 981, 936, 900, 890, 878, 853, 804, 756, 737, 726, 695, 661 cm^−1^; MS (ES+; CH_2_Cl_2_) *m/z*: 398.8243 [M + H]^+^; elemental analysis calcd (%) for C_11_H_11_Br_3_O (398.92): C 33.12, H 2.78; found: C 33.22, H 2.71.

##### 3,5-Bis[(dimethylamino)methyl]-4-bromopropiophenone (**20**)

To a cold solution (0 °C) of **19** (1.25 gr, 3.1 mmol) in dry diethyl ether (50 mL), an excess of anhydrous dimethylamine (10 mL) was added and the reaction mixture was allowed to warm to rt and stirred for 3 h. The resulting white suspension was washed with an aqueous NaOH solution (2M, 2 × 50 mL) and with brine (50 mL). The organic layer was dried using MgSO_4_ and evaporated to dryness to yield **20** as a yellow oil (0.96 g, 2.9 mmol, 94%). ^1^H-NMR (400 MHz, CDCl_3_): δ = 7.91 (s, 2H; ArH), 3.58 (s, 4H; NCH_2_), 3.01 (q, ^3^*J* (H,H) = 7.2 Hz, 2H; C*H*_2_CH_3_), 2.31 (s, 12H; N(CH_3_)_2_), 1.20 (t, ^3^*J* (H,H) = 7.2 Hz, 3H; C*H*_3_CH_2_); ^13^C{^1^H}-NMR (101 MHz, CDCl_3_): δ = 200.7 (C=O), 139.4 (Ar*C*CH_2_), 135.6 (Ar*C*C(O)), 132.4 (ArCBr), 129.0 (ArCH), 64.0 (CH_2_N), 45.8 (N(CH_3_)_2_), 32.2 (*C*H_2_C(O)), 8.4 (*C*H_3_CH_2_); IR (ATR): ῦ = 2974, 2941, 2904, 2857, 2818, 2770, 1686 (C=O), 1585, 14,456, 1406, 1377, 1358, 1343, 1265, 1174, 1093, 1040, 1019, 986, 949, 916, 900, 853, 840, 806, 730, 700 cm^−1^; MS (ES+; CH_2_Cl_2_) *m/z*: 327.0954 [M + H]^+^; elemental analysis calcd (%) for C_15_H_23_BrN_2_O (327.26): C 55.05, H 7.08, N 8.56; found: C 54.92, H 6.93, N 8.44.

##### [PtBr(NCN-C(O)Et-4)] (**21**)

A mixture of **20** (0.96 g, 2.9 mmol) and [Pt(tol-4)_2_(SEt_2_)]_2_ (1.31 g, 1.4 mmol) in toluene (30 mL) was heated at 75 °C for 3 h, after which the solvent was evaporated on the rotary evaporator. The residue was dissolved in a minimal amount of CH_2_Cl_2_ and upon the addition of pentane the product precipitated. After centrifugation, **21** was obtained as a light-yellow powder (1.40 g, 2.7 mmol, 96%). ^1^H-NMR (300 MHz, CDCl_3_): δ = 7.47 (s, 2H; ArH). 4.06 (s, ^3^*J*(H,Pt) = 45.9 Hz, 4H; NCH_2_), 3.13 (s, ^3^*J*(H,Pt) = 37.9 Hz, 12H; N(CH_3_)_2_), 2.89 (q, ^3^*J*(H,H) = 7.4 Hz, 2H; C*H*_2_CH_3_), 1.20 (t, ^3^*J*(H,H) = 7.4 Hz, 3H; CH_2_C*H*_3_); ^13^C{^1^H}-NMR (75 MHz, CDCl_3_): δ = 200.9 (C=O), 154.9 (C ipso Pt), 143.8, 133.4, 119.9, 77.4 (NCH_2_), 55.3 (N(CH_3_)_2_), 31.8 (*C*H_2_CH_3_), 8.9 (CH_2_*C*H_3_); IR (ATR): ῦ = 3057, 3013, 2969, 2916, 2889, 2830, 2783, 1655 (C=O), 1582, 1573, 1452, 1430, 1418, 1398, 1353, 1287, 1258, 1230, 1169, 1104, 1068, 1015, 990, 971, 943, 900, 863, 838, 801, 733, 705 cm^−1^; MS (ES+; CH_2_Cl_2_) *m/z*: 523.0468 [M + H]^+^; elemental analysis calcd (%) for C_15_H_23_BrN_2_OPt (522.34): C 34.49, H 4.44, N 5.36; found: C 34.61, H 4.43, N 5.27.

##### [PtCl(NCN-C(O)Et-4)] (**22**)

To a mixture of **21** (1.40 g, 2.7 mmol) in acetone (30 mL) AgBF_4_ (0.56 g, 2.9 mmol) was added, causing a white suspension. The mixture was covered with aluminum foil and stirred for 30 min, after which the aluminum foil was removed and the grey suspension was stirred for another 30 min. The suspension was filtered over Celite and the residue was subsequently washed with CH_2_Cl_2_ (20 mL), acetone (10 mL) and demineralized water (10 mL). A solution of NaCl (0.47 g, 8.0 mmol) in demineralized water (10 mL) was added, and the formed layers were separated. The aqueous layer was washed with CH_2_Cl_2_ (2 × 15 mL), and the combined organic fractions were dried using MgSO_4_ and evaporated to dryness. The residue was dissolved in a minimal amount of CH_2_Cl_2_, from which the product precipitated upon addition of pentane. After centrifugation **22** was isolated as a light-yellow powder (1.25 g, 2.6 mmol, 96%). ^1^H-NMR (300 MHz, CDCl_3_): δ = 7.46 (s, 2H; ArH). 4.06 (s, ^3^*J*(H,Pt) = 46.4 Hz, 4H; NCH_2_), 3.09 (s, ^3^*J*(H,Pt) = 37.9 Hz, 12H; N(CH_3_)_2_), 2.89 (q, ^3^*J*(H,H) = 7.4 Hz, 2H; C*H*_2_CH_3_), 1.21 (t, ^3^*J*(H,H) = 7.4 Hz, 3H; CH_2_C*H*_3_); ^13^C{^1^H}-NMR (75 MHz, CDCl_3_): δ = 200.6 (C=O), 153.9 (C ipso Pt), 143.5, 133.1, 119.5, 77.8 (NCH_2_), 54.4 (N(CH_3_)_2_), 31.5 (*C*H_2_CH_3_), 8.6 (CH_2_*C*H_3_); ^195^Pt{^1^H}-NMR (64 MHz, CD_2_Cl_2_): δ = –3116; IR (ATR): ῦ = 2976, 2920, 1661 (C=O), 1582, 1570, 1454, 1432, 1398, 1350, 1317, 1287, 1168, 1105, 1068, 1014, 978, 964, 944, 899, 880, 863, 836, 805, 735, 710 cm^−1^; MS (ES+; CH_2_Cl_2_) *m/z*: 479.1310 [M + H]^+^, 442.1285 [M–Cl]^+^; elemental analysis calcd (%) for C_15_H_23_N_2_OPtCl (477.89): C 37.70, H 4.85, N 5.86, found: C 37.86, H 4.95, N 5.76.

##### 4-[2-(Dimethylamino)ethoxy]-4′-hydroxybenzophenone (**24**)

The compound was synthesized according to a modified reaction procedure of Gauthier and coworkers [35], i.e., K_2_CO_3_ was used as the base and acetone was used as the solvent (instead of Cs_2_CO_3_ and DMF, respectively). 4,4′-Dihydroxybenzophenone (5.0 g, 23.3 mmol) was dissolved in a mixture of acetone (240 mL) and water (10 mL). To this mixture K_2_CO_3_ (10.0 g, 72.4 mmol), and 2-(dimethylamino)ethyl chloride hydrochloride (3.8 gr, 26.4 mmol) were added and the mixture was heated at reflux for 20 h. After cooling, the mixture was evaporated to dryness and taken up in a mixture of EtOAc:MeOH (1:1, 100 mL) and filtered. The filtrate was evaporated to dryness and the residue was triturated from CH_2_Cl_2_ with pentane. The residue was purified by silicagel column chromatography (EtOAc:MeOH, 1:1). The second fraction contained the desired product **24**, which was triturated from CH_2_Cl_2_ with pentane to yield the **24** as a light-yellow powder (1.4 g, 4.9 mmol; 21%). The analytical data obtained for **24** is in agreement with the published data. ^1^H-NMR (300 MHz, CD_3_OD): δ = 7.78 (d, ^3^*J* = 8.5 Hz, 2H; ArH), 7.71 (d, ^3^*J* = 8.5 Hz, 2H; ArH), 7.12 (d, ^3^*J* = 8.5 Hz; ArH), 6.92 (d, ^3^*J* = 8.5 Hz, 2H; ArH), 4.31 (t, ^3^*J* = 5.2 Hz, 2H; CH_2_O), 3.05 (t, ^3^*J* = 5.2 Hz, 2H; CH_2_N), 2.55 (s, 6H; CH_3_); ^13^C{^1^H}-NMR (300 MHz, CD_3_OD): δ = 196.8 (C=O), 163.4, 163.4, 133.7, 133.3, 132.3, 130.3, 116.2, 115.2, 66.3, 58.7, 45.5.

##### Attempted Protection of **24** with TBDMS-Cl

To a solution of **24** (0.26 g, 0.93 mmol) in dry THF (20 mL), *t*-butyldimethylsilyl chloride (TBDMS-Cl, 0.14 g, 0.94 mmol) and imidazole (64 mg, 0.94 mmol) were added. The mixture was stirred for 3 h and the suspension was filtered over Celite. The filtrate was extracted with ethyl acetate (3 × 20 mL) and the combined organic layers were washed with brine (2 × 100 mL), dried using MgSO_4_, filtered and evaporated to dryness. Only starting compound **24** was isolated. The same reaction was performed using NaH (60% dispersion in mineral oil) as base, again only starting compound **24** was isolated.

##### 4-Trimethylacetoxy-4′-[2-(dimethylamino)ethoxy]benzophenone (**25**)

A mixture of **28** (7.0 gr, 23.5 mmol), 2-(dimethylamino)ethyl chloride hydrochloride (8.5 g, 59.0 mmol), and K_2_CO_3_ (15.0 g, 0.11 mol) in acetone (250 mL) was heated at reflux for 20 h. After cooling to rt the mixture was evaporated to dryness, and subsequently EtOAc (150 mL) and water (100 mL) were added. The layers were separated and the aqueous layer was extracted with EtOAc (2 × 100 mL). The combined organic fractions were washed with brine (2 × 100 mL), dried using MgSO_4_, and evaporated to dryness. The residue was purified using column chromatography (Al_2_O_3_, CH_2_Cl_2_). The second fraction that came off the column contained the product **25** as a white solid (1.74 g, 4.7 mmol, 20%). ^1^H-NMR (400 MHz, CDCl_3_): δ = 7.81 (d, ^3^*J* = 8.8 Hz, 2H; ArH), 7.80 (d, ^3^*J* = 8.8 Hz, 2H; ArH), 7.17 (d, ^3^*J* = 8.8 Hz, 2H; ArH), 6.98 (d, ^3^*J* = 8.8 Hz, 2H; ArH), 4.24 (t, ^3^*J* = 5.4 Hz, 2H; CH_2_), 2.91 (t, ^3^*J* = 5.4 Hz, 2H; CH_2_), 2.47 (s, 6H; N(CH_3_)_2_), 1.38 (s, 9H; C(CH_3_)_3_); ^13^C{^1^H}-NMR (101 MHz, CDCl_3_): δ = 194.4 (Ar_2_C=O), 176.7, 162.1, 154.1, 135.5, 132.5, 131.3, 130.4, 121.4, 114.2, 65.7 (OCH_2_), 57.8 (N*C*H_2_CH_2_), 45.5 ((*C*H_3_)_2_NCH_2_CH_2_), 39.2 (*C*(CH_3_)_3_, 27.1 (C(*C*H_3_)_3_; IR (ATR): ῦ = 2956, 2818, 2769, 1750 (C=O pivaloyl), 1639 (C=O benzophenone), 1600 and 1575 (C–O), 1461, 1252, 1108, 1030, 928, 899, 845, 759, 682 cm^−1^; MS (ES+; CH_2_Cl_2_) *m/z*: 370.13 [M + H]^+^; elemental analysis calcd (%) for C_22_H_27_NO_4_ (369.45): C 71.51, H 7.37, N 3.79, found: C 71.39, H 7.32, N 3.71.

#### 4.1.2. McMurry Reaction

In a flame dried Schlenk tube an amount of TiCl_4_(THF)_2_ (6 equiv. in respect to the platinum aldehyde or ketone) was transferred via a glass bend from the TiCl_4_(THF)_2_ storage Schlenk. The complex was dissolved in dry THF (20 mL), and the yellow solution was added via a cannula to a stirred suspension of Zn (10 equiv. with respect to the platinum aldehyde or ketone) in dry THF (10 mL) at −20 °C. The mixture was heated at reflux for 2 h, and then allowed to cool to rt. To this solution a mixture of the platinum complex (1 equiv.) and the ketone (6 equiv. with respect to the platinum aldehyde or ketone) in dry THF (20 mL) was added dropwise, and the resulting mixture was heated at reflux for 16 h. After cooling to rt the mixture was hydrolyzed with a 10% K_2_CO_3_ solution (50 mL). Then EtOAc (100 mL) was added and the mixture was stirred for 15 min. The formed slurry was filtered over Celite, and from the filtrate the organic layer was separated. The organic layer was washed with brine (20 mL) and dried using MgSO_4_ and evaporated to dryness to afford the product with the reported yields. When necessary the compounds were further purified as indicated below.

##### Attempted Synthesis of 1-(1,1-Diphenyl-1-propenyl)-4-bromo-3,5-bis[(dimethylamino)methyl]Benzene (**9**); Formation of 1-(1,1-Diphenyl-1-propenyl)-3,5-bis[(dimethylamino)methyl]Benzene (**10**)

The reaction was performed according to the general McMurry procedure, using 4-bromo-3,5-bis[(dimethylamino)methyl]acetophenone **7** (0.28 g, 0.89 mmol) (instead of the platinum ketone) and benzophenone (as the ketone). After hydrolysis, the reaction mixture was extracted using diethyl ether, and the organic layer was dried over MgSO_4_. After filtration the volatiles were removed on the rotary evaporator leaving a product mixture. The crude product (0.9 g) was further purified by silica gel column chromatography, first eluting with CH_2_Cl_2_ to remove the excess of benzophenone, secondly a gradient elution starting with a CH_2_Cl_2_:MeOH (49:1) mixture changing to CH_2_Cl_2_:MeOH (9:1) ending with a solvent mixture of CH_2_Cl_2_:MeOH:pyridine (44:5:1). From the ^1^H-NMR spectrum it became clear that the isolated fraction did not contain the desired compound **9**, but mainly consisted of the dehalogenated compound **10**, together with a small amount of other impurities. For **10**: ^1^H-NMR (300 MHz, CDCl_3_): δ = 7.39-7.22 (m, ArH), 7.14 (s, 2H; ArH), 7.06–6.92 (m, ArH), 6.81 (s, 1H; ArH), 3.41 (s, 4H; CH_2_), 2.11 (s, 12H; (CH_3_)_2_N), 2.01 (s, 3H; CH_3_).

##### Attempted Synthesis of 3,3′,5,5′-Tetra(dimethylamino)methyl-4,4′-bisplatinumbromide-stilbene (**12**); Formation of 3,3′,5,5′-Tetra(dimethylaminomethyl)-4,4′-bisplatinum Halide-Benzophenone (**13**), (Halide = Br and Cl)

The reaction was performed according to the general McMurry procedure, using [PtBr(NCN-CHO-4)] **11** (0.60 g, 1.2 mmol), yielding a product mixture (340 mg) containing the starting aldehyde **11** and other compounds. The product mixture was precipitated from CH_2_Cl_2_ with pentane (2 ×), yielding a yellow powder (220 mg), consisting of multiple compounds. The mixture was crystallized from toluene giving yellow crystals (26 mg), which were determined with X-ray crystallography to be the bis(platinum halide)benzophenone **13** (halide = Br and Cl). Analytical data obtained for **13**: ^1^H-NMR (400 MHz, CD_2_Cl_2_): δ = 7.26 (s, 4H; ArH), 4.08 (s, ^3^*J*(H,Pt) = 42.0 Hz, 8H; CH_2_N), 3.12 and 3.07 (s, 24H; CH_3_N, X = Br or Cl); ^13^C{^1^H}-NMR (101 MHz, CD_2_Cl_2_): δ = 196.8 (C=O), 153.8 and 153.3 (C ipso to Pt, X = Br or Cl), 143.6 (Ar*C*CH_2_), 134.2 (Ar*C*C(O)), 121.5 (ArCH), 77.5 and 77.3 (CH_2_N, X = Br or Cl), 55.1 and 54.4 (CH_3_N); ^195^Pt{^1^H}-NMR (64 MHz, CD_2_Cl_2_): δ = −3107 and −3130; IR (ATR): ῦ = 3047, 3016, 2976, 2921, 2889, 2820, 2790, 1614 (C=O), 1579, 1557, 1467, 1456, 1425, 1402, 1390, 1346, 1317, 1265, 1177, 1144, 1098, 1068, 1017, 984, 960, 949, 885, 838, 777, 761, 718, 703 cm^−1^; MS (ES+; CH_2_Cl_2_) *m/z*: 949.0305 [M + H + Cl]^+^ (X = Cl, Br), 914.1157 [M + H]^+^ (X = Cl, Br), 905.1085 [M+H+Cl]^+^ (X = Cl), 869.1773 [M + H]^+^ (X = Cl), 833.1945 [M + H–Cl]^+^ (X = Cl).

##### 1-(2-(1-[(4-Dimethylaminoethoxy)phenyl]-1-phenyl-1-butenyl))-4-PtCl-3,5-bis[(dimethylamino)methyl]) Benzene (**5**)

The reaction was performed according to the general McMurry procedure, using **22** (0.58 g, 1.2 mmol) and **23** (1.96 g, 7.3 mmol), yielding a crude reaction product (2.29 g). The crude product was dissolved in a minimal amount of CH_2_Cl_2_, and upon addition of pentane a solid precipitated, which was isolated after centrifugation. This procedure was repeated and a solid was isolated (510 mg) containing **5** as the *E*/*Z* mixture (3:7 according to NMR), which was further purified with silica gel chromatography. A side product was eluted from the column with CH_2_Cl_2_, followed by a gradient elution, changing the eluent from pure CH_2_Cl_2_ to CH_2_Cl_2_:MeOH (9:1). One of the first fractions and one of the final fractions contained the separate *E-* (**5a**, 20 mg) and *Z*-isomer (**5b**, 15 mg), respectively, in pure form. The rest of the product fractions contained the product in a changing *E*:*Z* ratio. In total 310 mg (0.43 mmol, 36%) of product **5** was isolated, indicating the sum of the separately isolated product fractions. For **5a**: ^1^H-NMR (300 MHz, CDCl_3_): δ = 7.12 (d, ^3^*J*(H,H) = 8.5 Hz, 2H, ArH), 6.97 (m, 3H; ArH), 6.89–6.82 (m, 4H, ArH), 6.51 (s, 2H; ArH), 4.13 (t, ^3^*J*(H,H) = 5.8 Hz, 2H; OCH_2_), 3.86 (s, ^3^*J*(H,Pt) = 46.4 Hz, 4H; NCH_2_), 3.02 (s, ^3^*J*(H,Pt) = 35.7 Hz, 12H; N(CH_3_)_2_), 2.82 (t, ^3^*J*(H,H) = 5.8 Hz, 2H; NC*H*_2_CH_2_), 2.45 (q, ^3^*J*(H,H) = 7.4 Hz, 2H; C*H*_2_CH_3_), 2.41 (s, 6H; CH_2_CH_2_N(C*H*_3_)_2_), 0.97 (t, ^3^*J*(H,H) = 7.4 Hz, 3H; C*H*_3_CH_2_); ^13^C{^1^H}- NMR (75 MHz, CDCl_3_): δ = 157.3 (Ar*C*OCH_2_), 143.8, 143.0 (C ipso to Pt), 142.8, 142.5, 137.4, 137.3, 136.5, 130.8, 130.5, 127.1, 125.3, 120.7, 114.1, 77.5 (ArCH_2_N), 65.6 (OCH_2_), 58.2 (N*C*H_2_CH_2_), 54.2 (ArCH_2_N(*C*H_3_)_2_), 45.6 ((*C*H_3_)_2_NCH_2_CH_2_), 29.7 (C=C*C*H_2_CH_3_), 13.9 (*C*H_3_CH_2_C=C); ^195^Pt{^1^H}-NMR (64 MHz, CDCl_3_): δ = −3208; IR (ATR): ῦ = 3007, 2966, 2923, 2869, 2853, 2779, 1683, 1604, 1508, 1463, 1450, 1397, 1370, 1337, 1295, 1284, 1270, 1251, 1234, 1190, 1174, 1137, 1124, 1104, 1074, 1024, 982, 965, 941, 863, 835, 811, 763, 698, 662 cm^−1^; MS (ES+; CH_2_Cl_2_) *m/z*: 716.21 [M+H]^+^, 679.31 [M–Cl]^+^; elemental analysis calcd (%) for C_32_H_42_ClN_3_OPt (715.23): C 53.74 0, H 5.92, N 5.88, found: C 53.66, H 6.05, N 5.79.

For **5b**: ^1^H-NMR (300 MHz, CDCl_3_): δ = 7.35–7.19 (m, 5H; C_6_H_5_), 6.75 (d, ^3^*J* = 8.8 Hz, 2H; ArH), 6.56 (d, ^3^*J* = 8.8 Hz, 2H; ArH), 6.55 (s, 2H; ArH), 3.99 (t, ^3^*J*(H,H) = 5.6 Hz, 2H; OCH_2_), 3.89 (s, ^3^*J*(H,Pt) = broad, 4H; NCH_2_), 3.04 (s, ^3^*J*(H,Pt) = 34.6 Hz, 12H; N(CH_3_)_2_), 2.74 (t, ^3^*J*(H,H) = 5.6 Hz, 2H; NC*H*_2_CH_2_), 2.39 (q, ^3^*J*(H,H) = 7.4 Hz, 2H; C*H*_2_CH_3_), 2.36 (s, 6H; CH_2_CH_2_N(C*H*_3_)_2_), 0.94 (t, ^3^*J*(H,H) = 7.4 Hz, 3H; C*H*_3_CH_2_); ^13^C{^1^H}-NMR (75 MHz, CDCl_3_): δ = 156.4 (Ar*C*OCH_2_), 144.1, 143.0 (C_ipso_ to Pt), 142.6, 142.2, 137.5, 137.2, 136.1, 131.9, 129.4, 128.1, 126.4, 120.7, 113.3, 77.6 (ArCH_2_N), 65.5 (OCH_2_), 58.1 (N*C*H_2_CH_2_), 54.3 (ArCH_2_N(*C*H_3_)_2_), 45.6 ((*C*H_3_)_2_NCH_2_CH_2_), 29.7 (C=C*C*H_2_CH_3_), 13.9 (*C*H_3_CH_2_C=C); ^195^Pt{^1^H}- NMR (64 MHz, CDCl_3_): δ = −3207; IR (ATR): ῦ = 3032, 2955, 2922, 2868, 2855, 2767, 1667, 1603, 1571, 1508, 1492, 1449, 1402, 1372, 1335, 1286, 1244, 1173, 1138, 1124, 1024, 960, 945, 913, 865, 829, 804, 774, 727, 703, 667 cm^−1^; MS (ES+; CH_2_Cl_2_) *m/z*: 716.25 [M + H]^+^, 679.26 [M–Cl]^+^; elemental analysis calcd (%) for C_32_H_42_ClN_3_OPt (715.23): C 53.74 0, H 5.92, N 5.88, found: C 53.82, H 6.08, N 5.81.

##### 1-(4-[2-(Dimethylamino)ethoxy]phenyl)-1-(4-trimethylacetoxyphenyl)-2-(NCN-PtCl)but-1-ene (**26**)

The reaction was performed according to the general McMurry procedure, using **22** (378 mg, 0.79 mmol) and **25** (1.7 g, 4.7 mmol). After extraction of the reaction mixture with CH_2_Cl_2_, the crude product was isolated after evaporation and was purified with silica gel chromatography with a gradient elution. The eluent was changed from CH_2_Cl_2_:MeOH (9:1) to CH_2_Cl_2_:MeOH (5:1) to CH_2_Cl_2_:MeOH:Et_3_N (80:19:1). The second fraction collected contained the desired product **26** (*E-*:*Z*-mixture, 7:9, based on NMR) as a white solid (0.40 g, 0.49 mmol, 62%). A subsequent separation of the isomers was performed by silica gel flash chromatography (SiO_2_, CH_2_Cl_2_:MeOH, 9:1) None of the fractions contained the pure (either *E-* or *Z-*) isomer, best ratio was 1:49 (based on NMR). Major isomer of **26**. ^1^H-NMR (400 MHz, C_6_D_6_): δ = 7.27 (d, ^3^*J*(H,H) = 8.8 Hz, 2H; ArH), 7.07 (d, ^3^*J*(H,H) = 8.4 Hz, 2H; ArH), 6.86 (d, ^3^*J*(H,H) = 8.8 Hz, 2H; ArH), 6.75 (d, ^3^*J*(H,H) = 8.4 Hz, 2H; ArH), 6.54 (s, 2H; ArH), 3.83 (t, ^3^*J*(H,H) = 6.0 Hz, 2H; OC*H*_2_CH_2_), 3.27 (s, ^3^*J*(H,Pt) = broad, 4H; ArC*H*_2_N(CH_3_)_2_), 2.69 (q, ^3^*J*(H,H) = 7.6 Hz, 2H; C*H*_2_CH_3_), 2.67 (s, ^3^*J*(H,Pt) = broad, 12H; ArCH_2_N(C*H*_3_)_2_), 2.50 (t, ^3^*J*(H,H) = 5.6 Hz, 2H; OCH_2_C*H*_2_), 2.08 (s, 6H; CH_2_CH_2_N(C*H*_3_)_2_), 1.18 (t, ^3^*J*(H,H) = 7.6 Hz, 3H; C*H*_3_CH_2_), 1.07 (s, 9H; C(O)(CH_3_)_3_); ^13^C{^1^H}-NMR (101 MHz, C_6_D_6_): δ = 177.2 (C=O), 157.6, 149.1, 143.7 (C*_ipso_* to Pt), 143.1, 137.3, 136.4, 131.8, 130.7, 130.5, 121.3, 120.9, 120.4, 114.3, 113.4, 77.7 (Ar*C*H_2_N), 65.8 (OCH_2_), 58.2 (N*C*H_2_CH_2_), 54.3 (ArCH_2_N(*C*H_3_)_2_), 45.5 ((*C*H_3_)_2_NCH_2_CH_2_), 39.0 (*C*(CH_3_)_3_), 29.9 (C=C*C*H_2_CH_3_), 27.0 (C(*C*H_3_)_3_), 13.7 (*C*H_3_CH_2_C=C); δ = ^195^Pt{^1^H}-NMR (64 MHz, CD_2_Cl_2_): δ = −3192; IR (ATR): ῦ = 2971, 2928, 1748 (C=O stretch of pivaloyl group), 1606 (C–O stretch), 1505, 1462, 1241, 1198, 1164, 1116, 1029, 1016, 833 cm^−1^. Minor isomer of **26**. ^1^H-NMR (400 MHz, C_6_D_6_): δ = 7.33 (d, ^3^*J*(H,H) = 8.8 Hz, 2H; ArH), 7.08 (d, ^3^*J*(H,H) = 8.4 Hz) 7.00 (d, ^3^*J* = 8.8 Hz, 2H; ArH), 6.60 (d, ^3^*J* = 8.4 Hz, 2H; ArH), 6.59 (s, 2H; ArH), 3.65 (t, ^3^*J* = 5.8 Hz, OC*H*_2_CH_2_), 3.20 (s, ^3^*J*(H,Pt) = broad, 4H; ArC*H*_2_N(CH_3_)_2_), 2.63 (s, ^3^*J*(H,Pt) = broad, 12H; ArCH_2_N(C*H*_3_)_2_), 2.58 (q, ^3^*J*(H,H) = 7.6 Hz, 2H; C*H*_2_CH_3_), 2.37 (t, ^3^*J*(H,H) = 6.0 Hz, 2H; OCH_2_C*H*_2_), 1.99 (s, 6H; CH_2_CH_2_N(C*H*_3_)_2_), 1.21 (s, 9H; C(O)(CH_3_)_3_), 0.83 (t, ^3^*J*(H,H) = 7.0 Hz, 3H; C*H*_3_CH_2_).

### 4.2. X-ray Crystal Structure Determination of Compound ***13***

C_25_H_36_Br_0.82_Cl_1.18_N_4_OPt_2_ + disordered solvent, Fw = 906.11^[*]^, yellow needle, 0.39 × 0.12 × 0.09 mm^3^, monoclinic, C2/c (no. 15), a = 21.3275(11), b = 13.8471(9), c = 11.9515(8) Å, β = 93.981(5)°, V = 3521.1(4) Å^3^, Z = 4, D_x_ = 1.709 g/cm^3[*]^, μ = 8.98 mm^−1[*]^. The diffraction experiment was performed on a Nonius KappaCCD diffractometer with rotating anode and graphite monochromator (λ = 0.71073 Å) at a temperature of 150(2) K up to a resolution of (sin θ/λ)_max_ = 0.65 Å^−1^. The Eval-14 software [56] was used for the intensity integration. An analytical absorption correction was performed with PLATON [57] (correction range 0.06–0.58). The SortAV software [58] was used for scaling and merging. A total of 34116 reflections was measured, 4050 reflections were unique (R_int_ = 0.042), 3435 reflections were observed [I > 2σ(I)]. The structure was solved with Patterson methods using DIRDIF99 [59]. Structure refinement was performed with SHELXL-2018 [60] on F^2^ of all reflections. The crystal structure contains large voids (1068 Å^3^/unit cell) filled with severely disordered solvent molecules. Their contribution to the structure factors was secured by the SQUEEZE algorithm [61] resulting in 344 electrons/unit cell. Non-hydrogen atoms were refined freely with anisotropic displacement parameters. Hydrogen atoms were introduced in calculated positions and refined with a riding model. On the halogen site there is substitutional disorder between bromine and chlorine. 163 Parameters were refined with two restraints (Pt-Br and Pt-Cl distances). R1/wR2 [I > 2σ(I)]: 0.0169/0.0357. R1/wR2 [all refl.]: 0.0242/0.0375. S = 1.038. Residual electron density between −0.68 and 0.73 e/Å^3^. Geometry calculations and checking for higher symmetry was performed with the PLATON program [57]. Derived values do not contain the contribution of the disordered solvent. For detailed information on **5b**, see [34]. 

## Data Availability

All data are contained within the article and the Supplementary Materials.

## Supplementary Materials

The following are available online, Figure S1: ESI mass spectrum of bis(NCN-pincer platinum halide)benzophenone **13**, showing the halogen scrambling (Br^−^ vs. Cl^−^) on the platinum centers, Table S1: Relevant ^13^C{^1^H}, ^195^Pt{^1^H} NMR and IR data including those of the NCN arylpincer platinum halide substituted compounds^a^ (for all data see Experimental Section). Table S2: Selected bond lengths [Å] and angles [°] of **13**. CCDC 2062971 contains the supplementary crystallographic data for this paper. These data can be obtained free of charge from The Cambridge Crystallographic Data Centre via www.ccdc.cam.ac.uk/structures. Comparison of the structural features of **5b**, **1** and **3**.

## Sample Availability

Samples of the compounds are not available from the authors.
