# Supplementary material for: Replacing the Z-phenyl Ring in Tamoxifen® with a para-Connected NCN Pincer-Pt-Cl Grouping by Post-Modification
Source: Molecules. 2021 Mar 26;26(7):1888. doi: 10.3390/molecules26071888 (PMC8038112; doi:10.3390/molecules26071888)
Supplement: Supplementary file 1 [file molecules-26-01888-s001.pdf]

Supplementary Materials

Replacing the *Z*-phenyl ring in Tamoxifen® for a *para*-connected NCN pincer-Pt-Cl grouping by post-modification

Guido D. Batema,[a] Ties J. Korstanje,[a] Gabriela Guillena,[a] Gema Rodríguez,[a] Martin Lutz,[b] Huub Kooijman, Anthony L. Spek,[b] Gerard P.M. van Klink,[a] Robert A. Gossage[c] and Gerard van Koten*[a]§

Content:

1. Fig. S1: ESI mass spectrum of bis platinum pincer benzophenone **13**, with halogen scrambling (Br^- and Cl^-) on the platinum centers.
2. Table S1 of relevant $^{13}C\{^1H\}$, $^{195}Pt\{^1H\}$ NMR and IR data including those of the NCN arylpincer platinum halide substituted compounds.
3. Relevant IR and NMR spectra
4. X-ray data Table S2 and files
5. Comparison of the structural features of **5b**, **1** and **3**.

1. *Figure S1: ESI mass spectrum of bis(platinum pincer)benzophenone* **13**

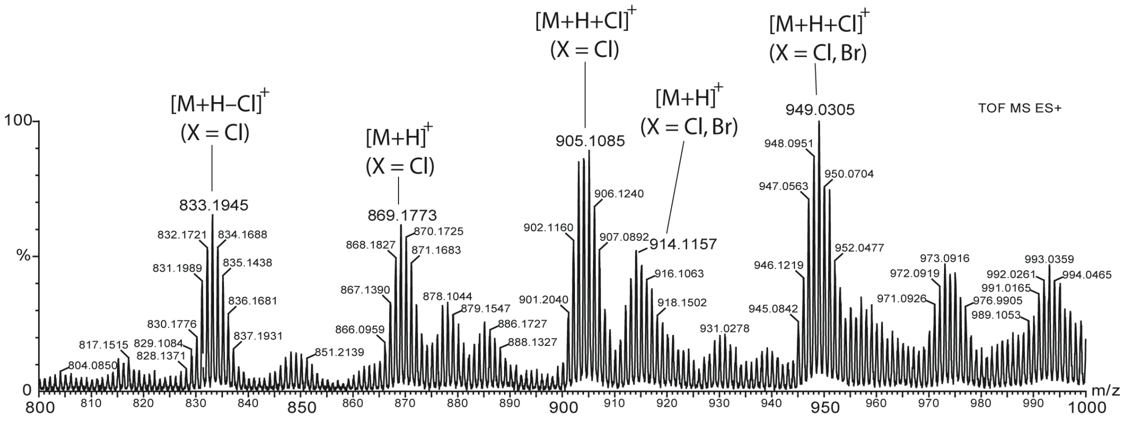

ESI mass spectrum of bis(platinumpincer)-benzophenone **13**, with halogen scrambling (Br^- and Cl^-) on the platinum centers.

2. *Table S1: Relevant $^{13}C\{^1H\}$, $^{195}Pt\{^1H\}$ NMR and IR data including those of the NCN arylpincer platinum halide substituted compounds[a] (for all data see Experimental Section).*

Compound	δ ^{13}C (C=O) {^{1}H} (ppm	δ ^{13}C *ipso* {^{1}H} (ppm)[c]	δ ^{195}Pt{^{1}H} (ppm)[d]	IR νC=O (stretch, cm^{-1})
4-Br-3,5-bis(Me_2NCH_2)acetophenone (**7**)	197.8[e]			1683
[PtBr(NCN-C(O)Me-4)] (**14**)	197.7	155.3		1663
[PtCl(NCN-C(O)Me-4)] (**15**)	197.7	154.2	–3101	1663
3,5-Me_2-4-Br-propiophenone (**18**)	200.1			1674
3,5-bis($BrCH_2$)-4-Br-propiophenone (**19**)	199.0			1689
3,5- bis(Me_2NCH_2)-4-bromo-propiophenone (**20**)	200.7			1686
[PtBr(NCN-C(O)Et-4)] (**21**)	200.9[b]	154.9[b]		1655
[PtCl(NCN-C(O)Et-4)] (**22**)	200.6[b]	153.9[b]	–3116[a]	1661
[PtCl(NCN(C_2H_5C=C(C_6H_5)($C_6H_4OC_2H_4NMe_2$-4')-4)-*E*][b] (**5a**)		143.0	–3208	
[PtCl(NCN(C_2H_5C=C(C_6H_5)($C_6H_4OC_2H_4NMe_2$-4')-4)-*Z*][b] (**5b**)		143.0	–3207	
4-Trimethylacetoxy-4'-[2-$Me_2NCH_2CH_2O$]-benzophenone (**25**)				1750[f], 1639[g]
[PtCl(NCN(C_2H_5C=C(C_6H_4OPiv-4')-($C_6H_4OC_2H_4NMe_2$-4")-4)-*X*][a] **(26)**, *X*= *E* or *Z*			–3192	1748[f]
Bis(NCNPtX)$_2$benzophenone (**13**), X=Br or Cl	196.8	153.8 and 153.3	–3107 and –3130	1614
Benzophenone				1664[h]

[a] in CD_2Cl_2; [b] in $CDCl_3$; [c] chemical shift of C_{ipso} to Pt; [d] Na_2PtCl_6 as external reference; [e] obtained from ref [22]; [f] acetyl; [g] benzophenone; obtained from ref. Shani et al. *J. Med. Chem.* **1985**, *28*, 1504-1511.

Structural features in solution

NMR characterization. The NMR data of the various ketone and pincercifen platinum compounds are compiled in Table S2. The proton signals of the *E*/*Z*-isomers of **5** were assigned for the separate isomers,

using the general comments on a series of tamoxifen analogues, as described by Shani and coworkers, see table S1. Indeed, for the *Z*-isomer, the resonances of all the protons in the basic chain and 2-(dimethylamino)ethoxy group were found at higher field compared to the *E*-form. This assumption was confirmed by the elucidation of the molecular structure in the solid state for **5b**.

In the $^{13}C\{^{1}H\}$ NMR spectra of **14** a two (^{2}J(C,Pt) = 77 Hz) and three bond coupling (^{3}J(C,Pt) = 35 Hz) of platinum to carbon was observed, for the other compounds these couplings were not resolved. Due to the strong electron withdrawing character of the carbonyl oxygen, the signal of the de-shielded carbonyl carbon atom of the C=O containing molecules can be found down-field between 196.8 and 200.9 ppm. Introduction of the platinum on the ligand backbone, had no clear effect on the shielding of the carbonyl carbon atoms. The chemical shift of the carbon atom bound to Pt, C_{ipso}, of the metallated ketones, are found between 153.3 and 155.3 ppm, which is in agreement with shift data of other *para*-substituted NCN-pincer platinum complexes containing *para*-substituents with an electron withdrawing character [37]. The shift of the C*ipso* found for the Pincercifen platinum complexes **5**, which are part of a conjugated system, were found at 143 ppm, which is in agreement with shift data found in earlier studies for stilbenoid NCN-pincer platinum complexes [47].

For the $^{195}Pt\{^{1}H\}$ NMR shifts of the metal complexes a similar trend is observed as what was found for the C_{ipso} carbon atoms. The complexes which contain the more electron withdrawing carbonyl group (**13**, **15** and **22**) situated on the *para* position of the Pt center, show resonance signals at lower field between –3101 and –3130 ppm. The Pt centers from the conjugated molecule **5** is more shielded and their signals is found at higher field between –3192 ppm, also in agreement with the data found for other NCN pincer platinum complexes. The shifts of the *E*- and Z-isomer of **5** differ only by 1 ppm.

With respect to the platinum pincer derivatives, in the ^{1}H NMR spectra resonances for the $(CH_3)_2N$ and the $ArCH_2N$ protons of the CH_2NMe_2 substituents were observed at δ = 3.02-3.13 ppm and at δ = 4.06-4.07 ppm, respectively, except for **5**, which showed the resonances of the CH_2 protons at higher field, at δ = 3.86-3.89 ppm. All the resonances of the CH_2NMe_2 substituents showed characteristic satellites resulting from platinum coupling (^{3}J(H,Pt) ≈ 35-38 Hz and 45-46 Hz, for the CH_3 and CH_2 resonances, respectively).

For the bispincer-benzophenone **13**, the presence of different halido ligands (Cl and Br) could be observed as two signals for the NMe_2 protons at 3.07 and 3.12 ppm. The chemical shift of the CH_2N protons were not influenced by the electronic effect of the different halides, and only one signal at 4.08 ppm (containing the Pt satellites) was observed.

With a combination of COSY and NOESY NMR the identity of the major isomer in the final fraction of **26** was hoped to be elucidated. Although the peaks to the individual compounds could be assigned as a result from the COSY spectrum (and by use of the peak intensities), it was not possible from the NOESY to assign unequivocally to either the *E*- or *Z*-isomer. The relevant COSY and NOESY spectra go included, *vide infra*.

As a result of the more important pivaloyl group, the *E* (**26b**) and *Z* (**26a**) assignments change when compared to the assignment of **5a** (*E*) and **5b** (*Z*).

IR spectroscopy. The molecules which contain the IR active carbonyl group, all show the carbonyl stretch frequency in the expected 1689-1614 cm^{-1} region. The introduction of platinum into **7** and **20**, is accompanied by the occurrence of the C=O band for **14** and **21**, respectively, at lower wavenumbers, because substitution of the electronegative bromide with the electron donating platinum [37], reduces the double-bond character of the C=O bond. The presence of two donating platinum centers in **13** moves the carbonyl absorption band to 1614 cm^{-1}, which is at lower wavenumbers comparing with normal benzophenone, which shows the carbonyl absorption at 1664 cm^{-1}.

3. *Relevant IR and NMR spectra*

4-Bromo-3,5-bis [(dimethylamino)methyl]acetophenone (7). IR (ATR):

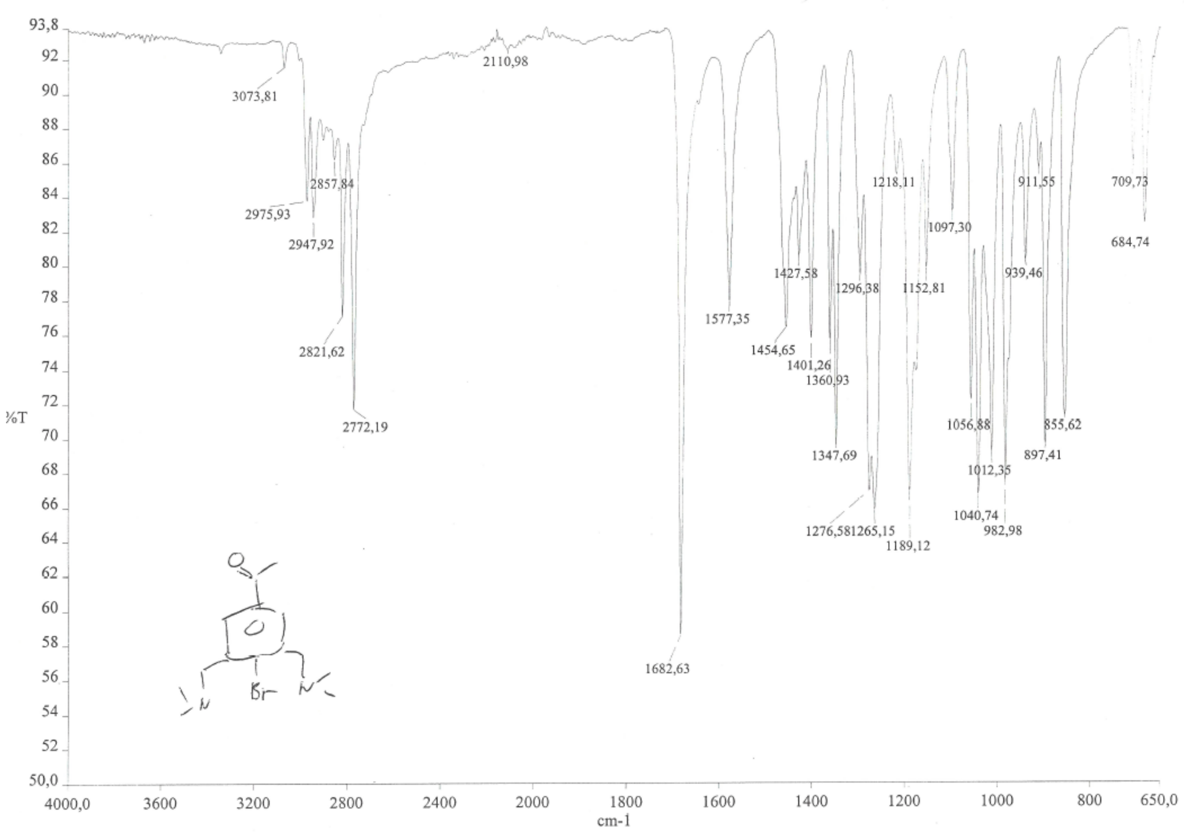

[PtBr(NCN-C(O)Me-4)] (14). ^{1}H NMR (400 MHz, CD_2Cl_2):

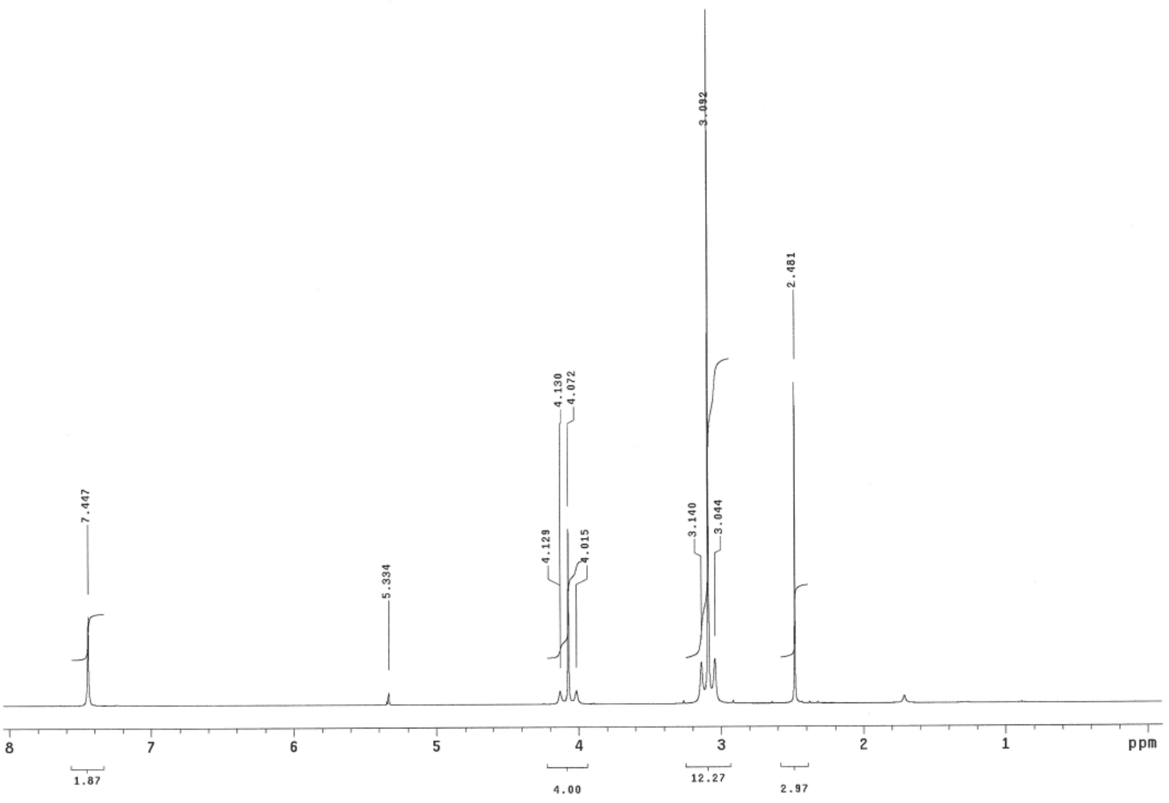

[PtBr(NCN-C(O)Me-4)] (14). $^{13}C\{^{1}H\}$ NMR (101 MHz, CD_2Cl_2):

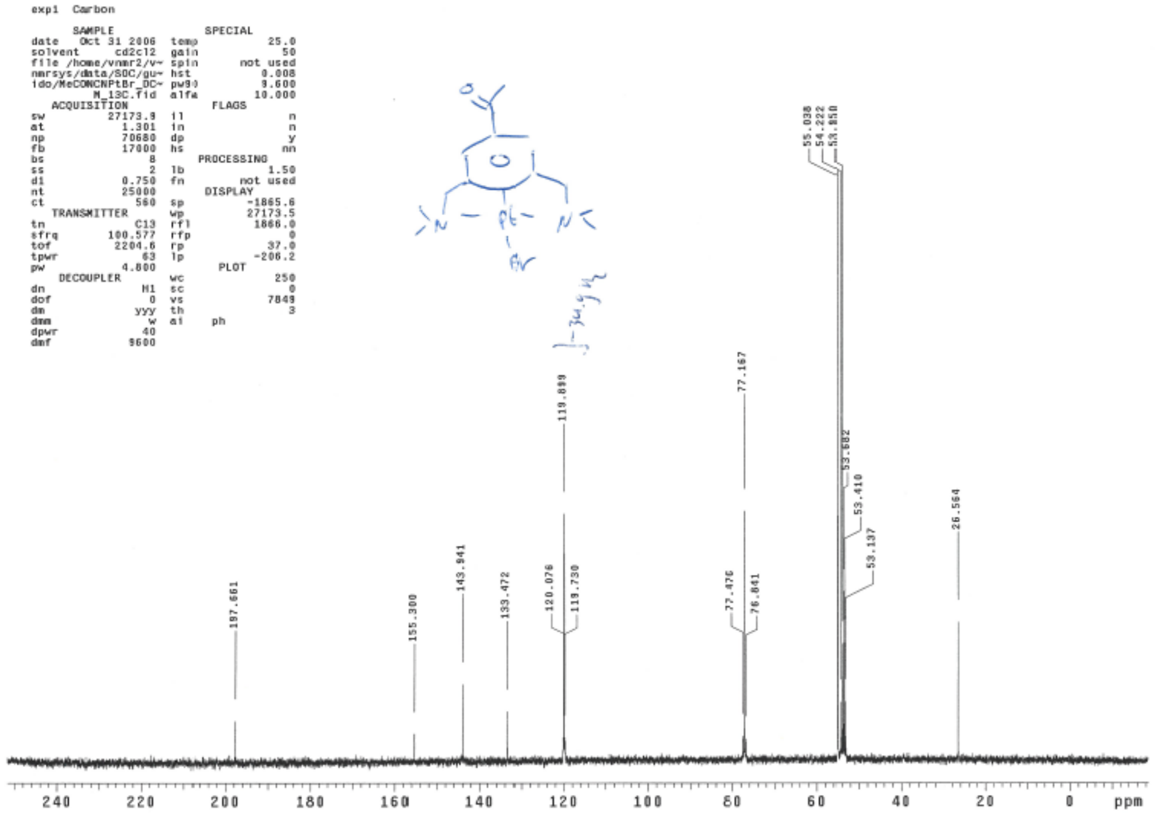

[PtBr(NCN-C(O)Me-4)] (14). IR (ATR):

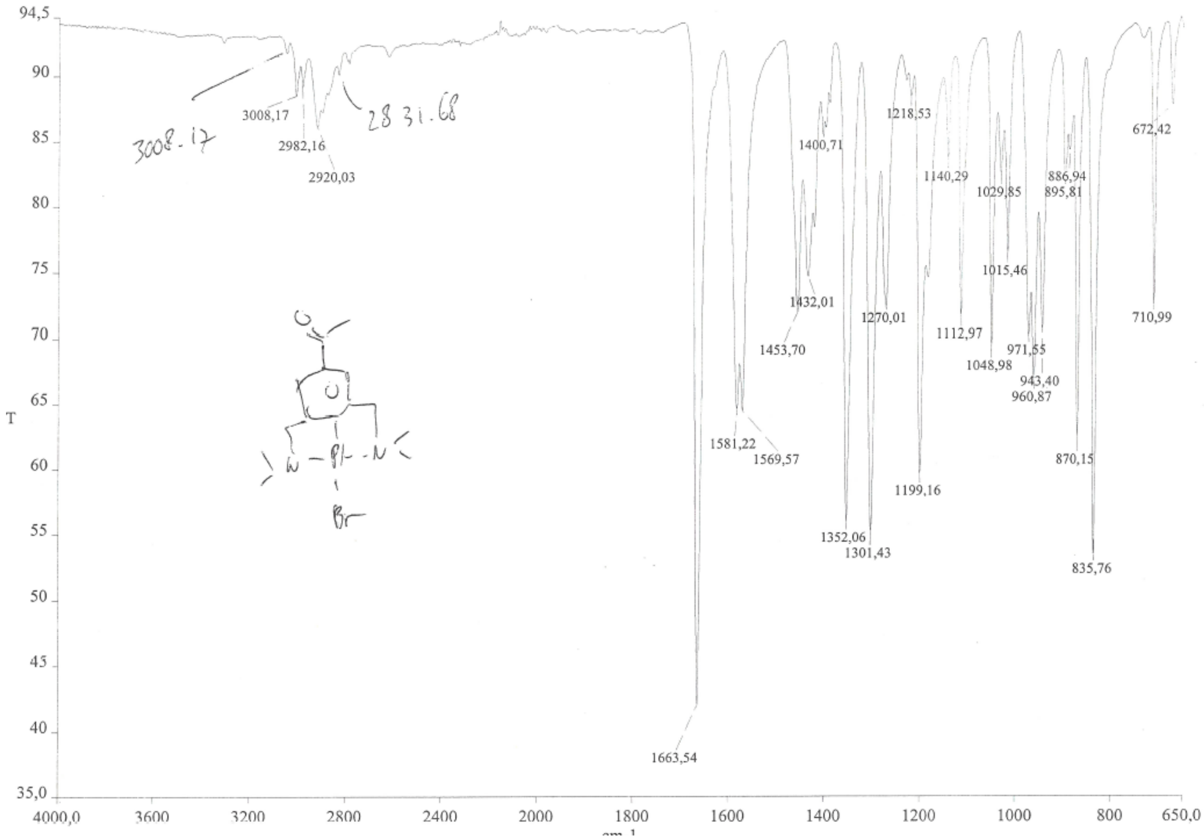

[PtCl(NCN-C(O)Me-4)] (15). ^{1}H NMR (300 MHz, CD_2Cl_2):

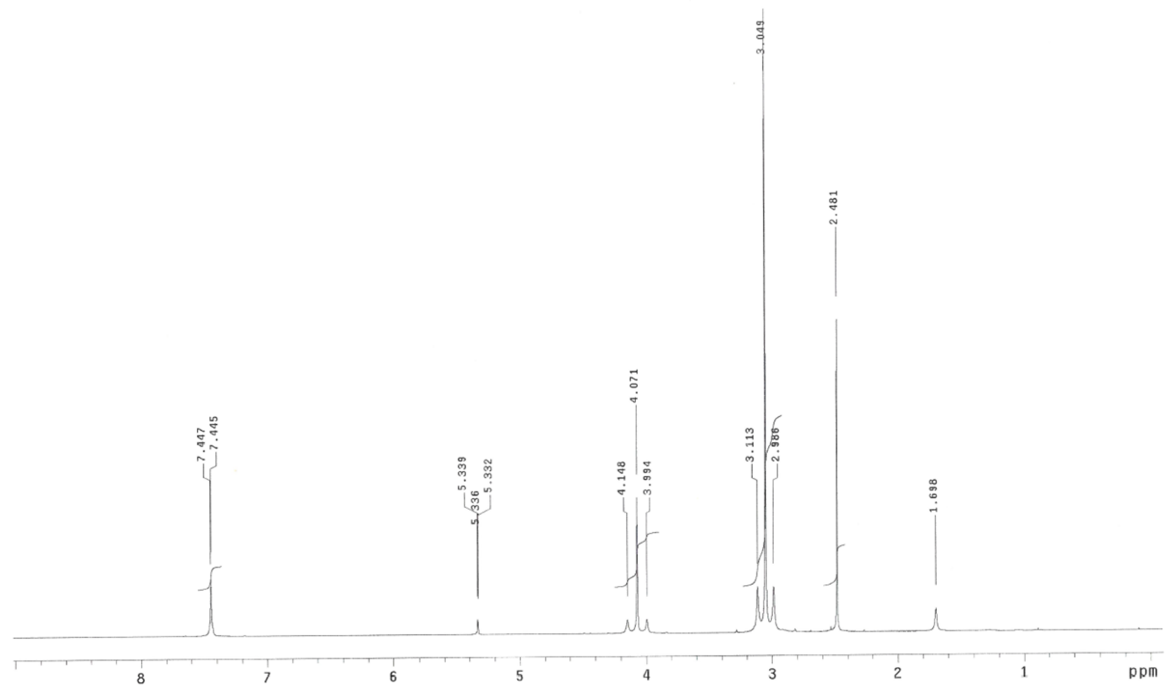

[PtCl(NCN-C(O)Me-4)] (15). $^{13}C\{^1H\}$ NMR (75 MHz, $CDCl_3$):

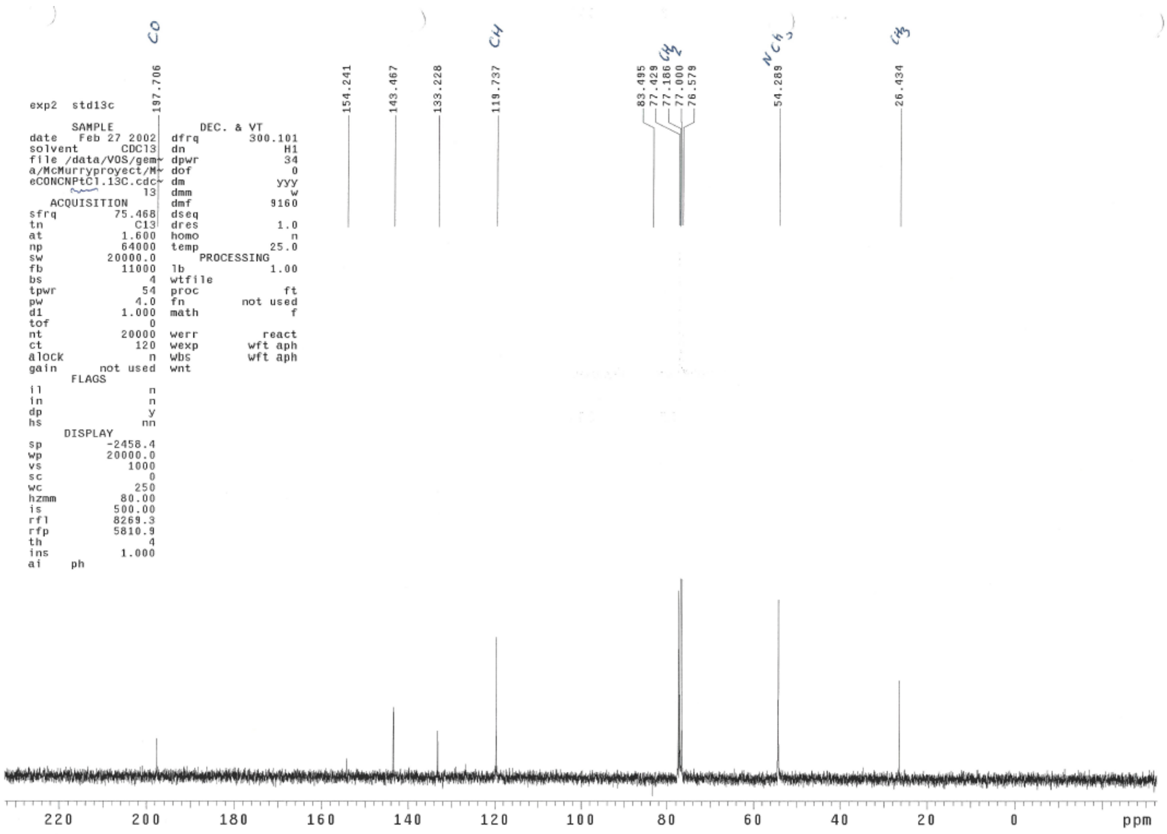

[PtCl(NCN-C(O)Me-4)] (15). $^{195}Pt\{^1H\}$ NMR (64 MHz, CD_2Cl_2):

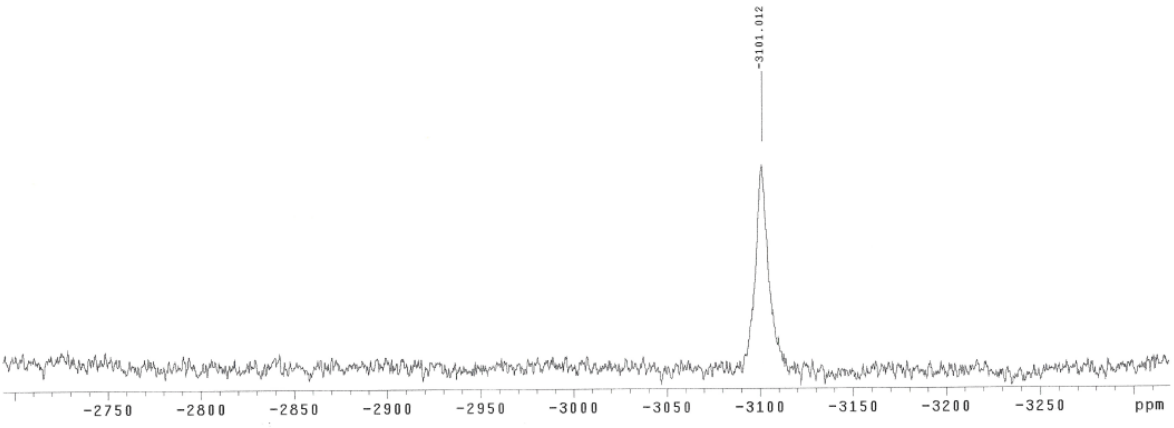

[PtCl(NCN-C(O)Me-4)] (15). IR (ATR):

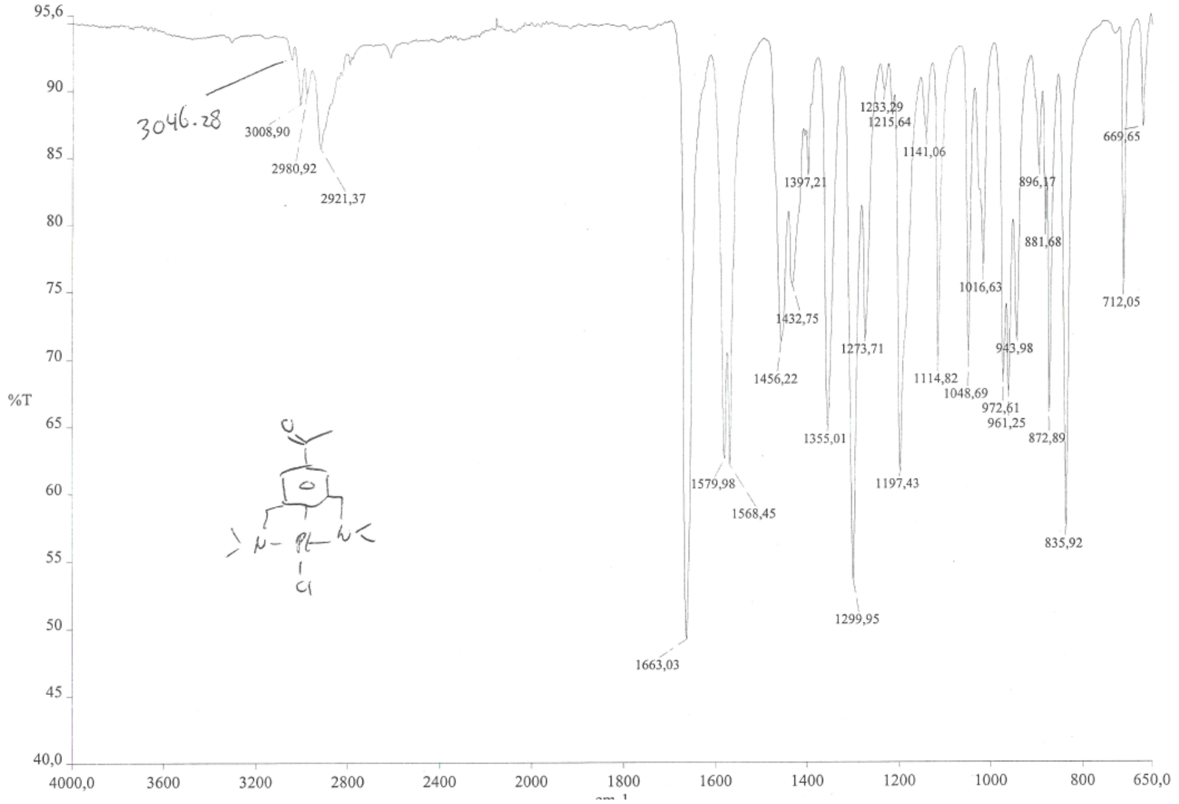

3,5-Dimethyl-4-bromo-propiophenone (18). ^{1}H NMR (200 MHz, $CDCl_3$):

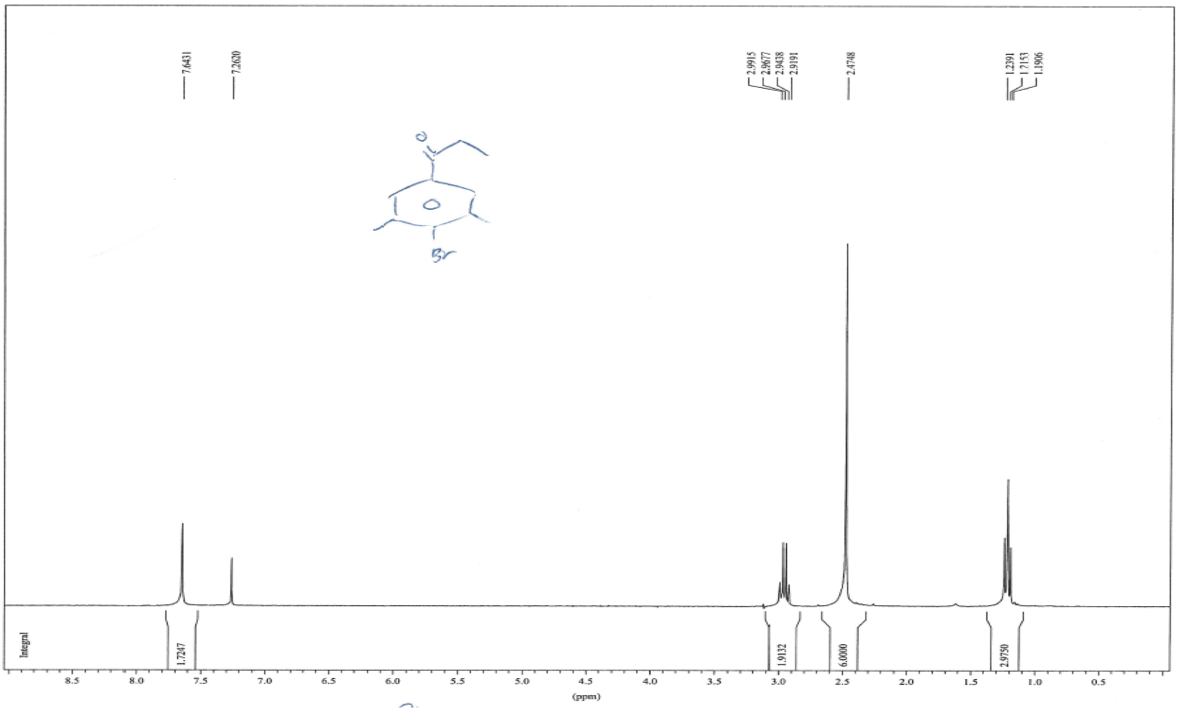

3,5-Dimethyl-4-bromo-propiophenone (18). $^{13}C\{^{1}H\}$ NMR (75 MHz, $CDCl_3$):

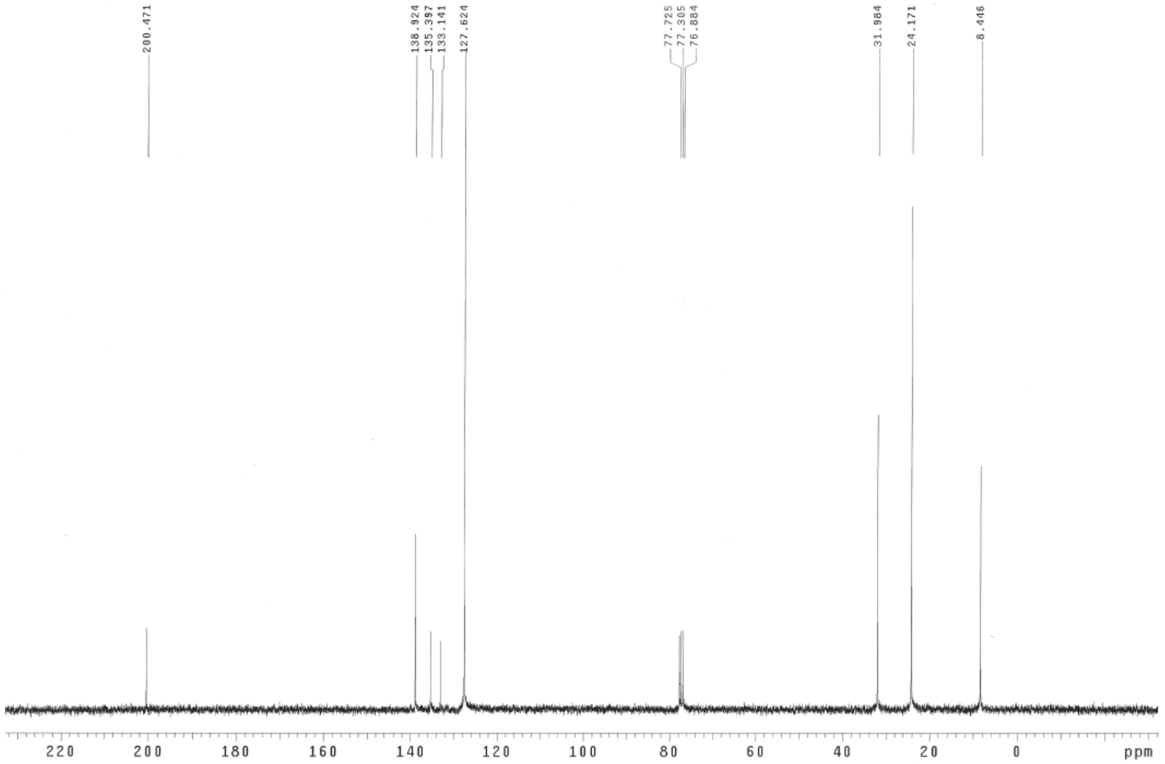

3,5-Dimethyl-4-bromo-propiophenone (18). IR (ATR):

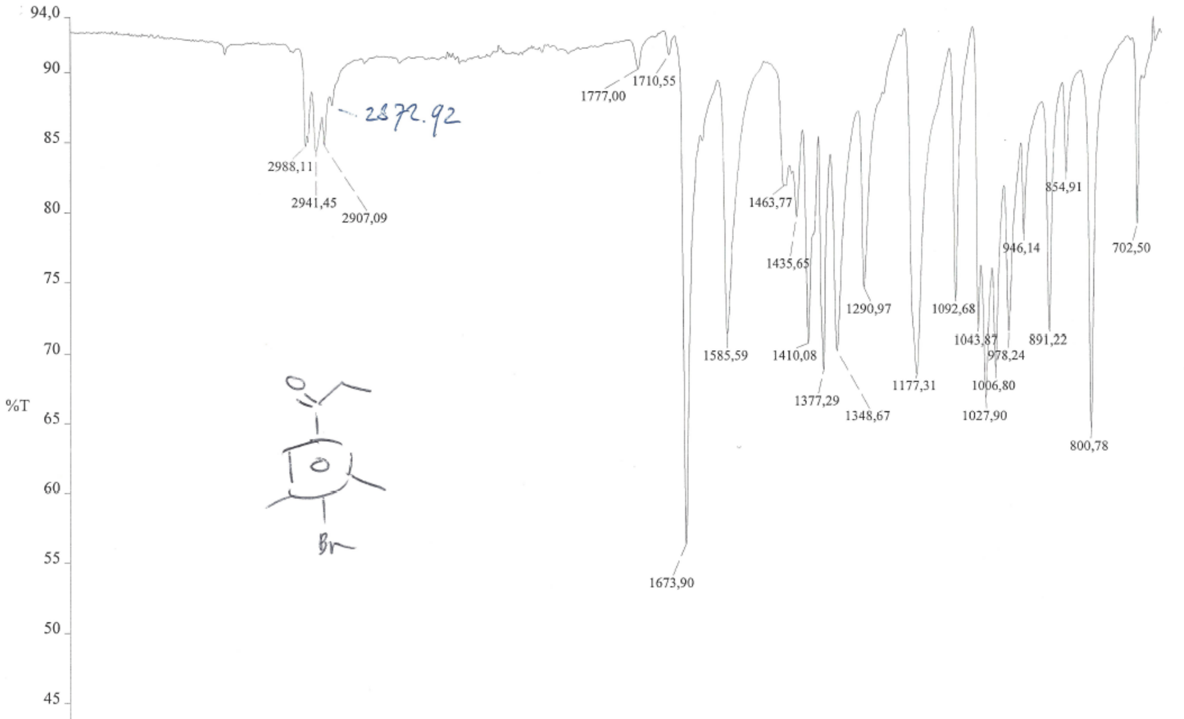

3,5-Bis(bromomethyl)-4-bromo-propiophenone (19). ^{1}H NMR (400 MHz, $CDCl_3$):

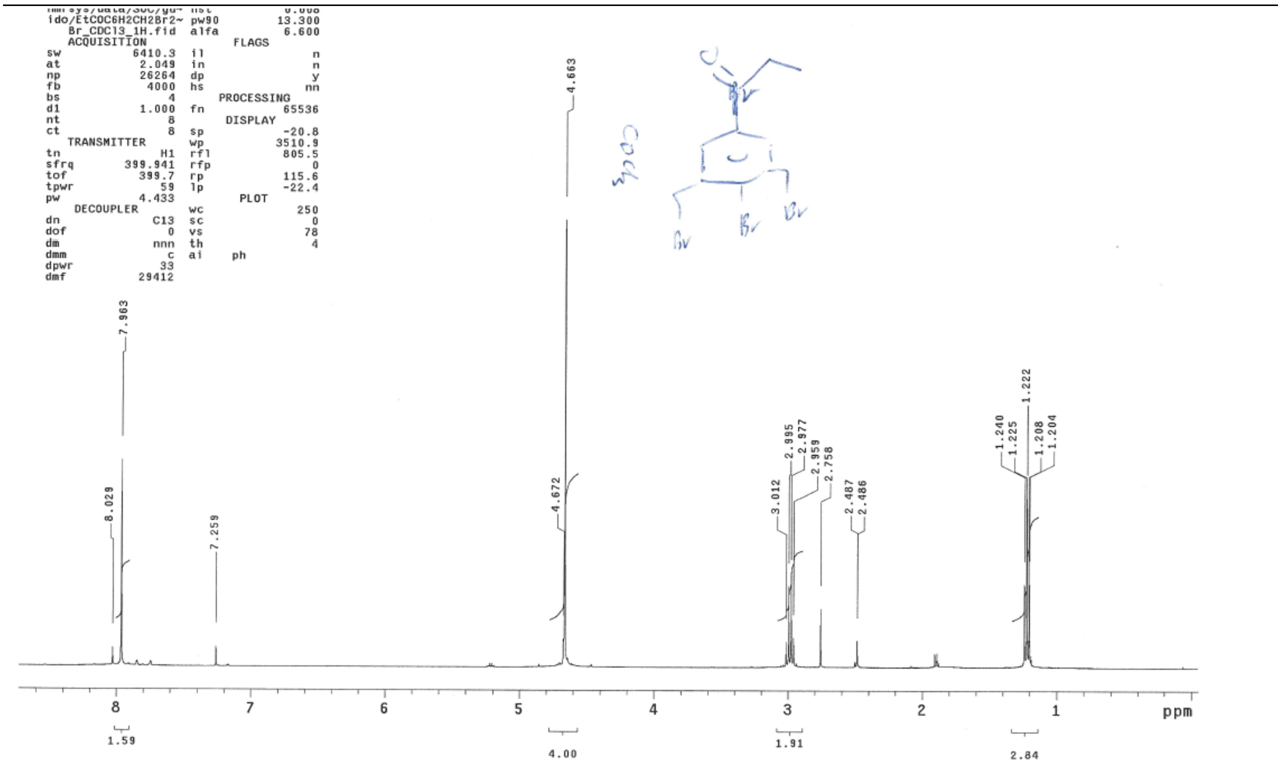

3,5-Bis(bromomethyl)-4-bromo-propiophenone (19). $^{13}C\{^{1}H\}$ NMR (101 MHz, $CDCl_3$):

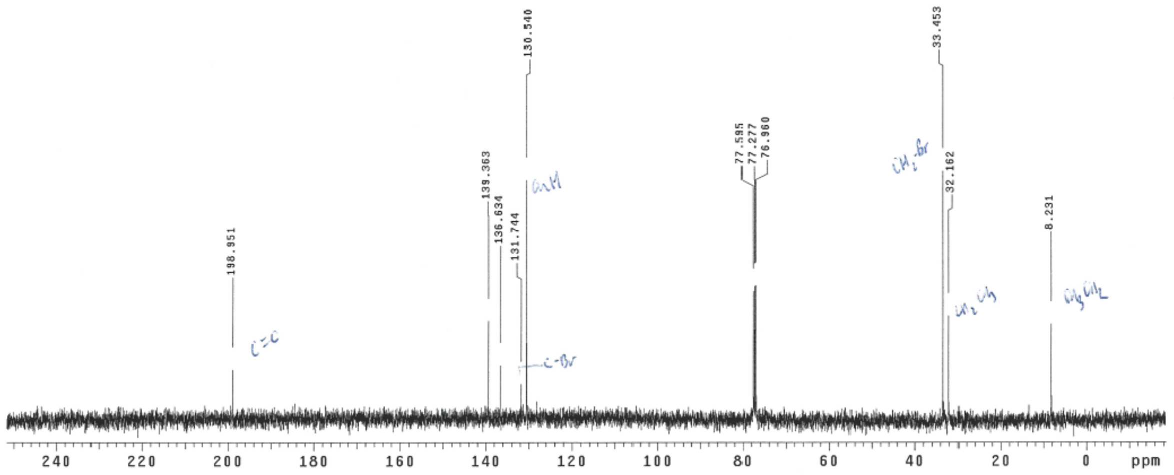

3,5-Bis(bromomethyl)-4-bromo-propiophenone (19). IR (ATR):

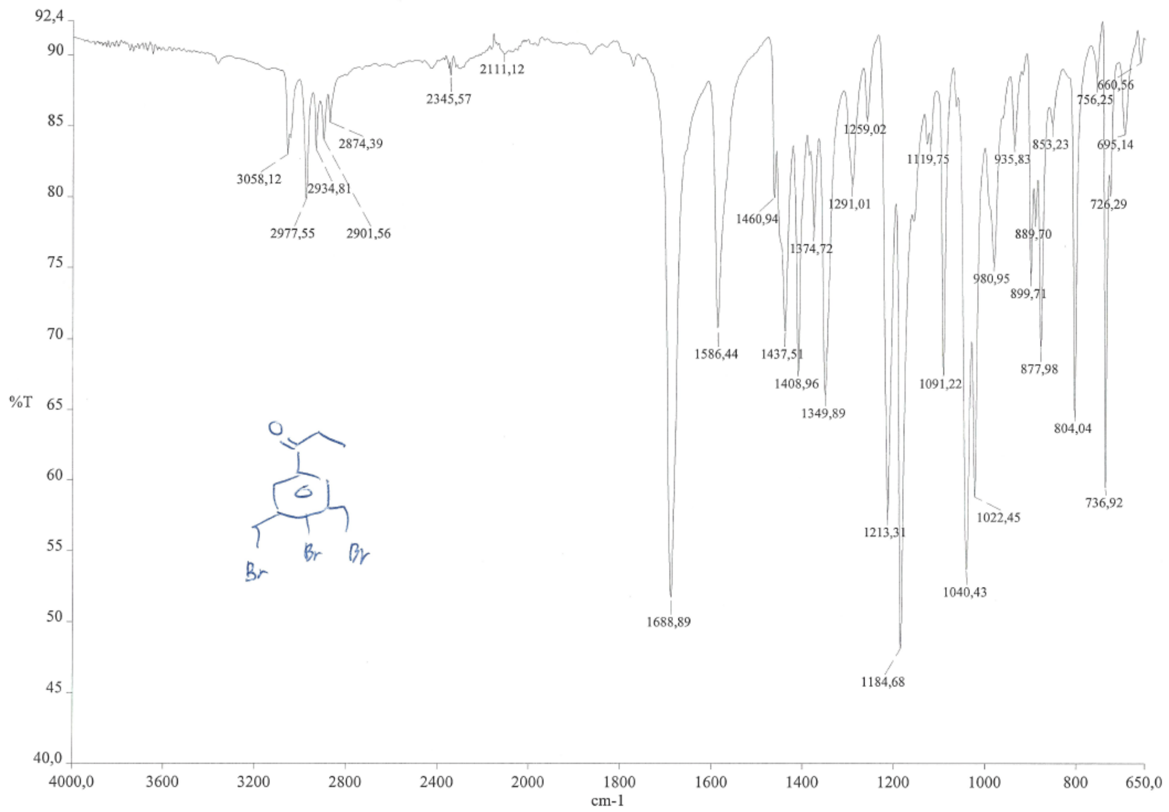

3,5-Bis[(dimethylamino)methyl]-4-bromo-propiophenone (20). ^{1}H NMR (400 MHz, $CDCl_3$):

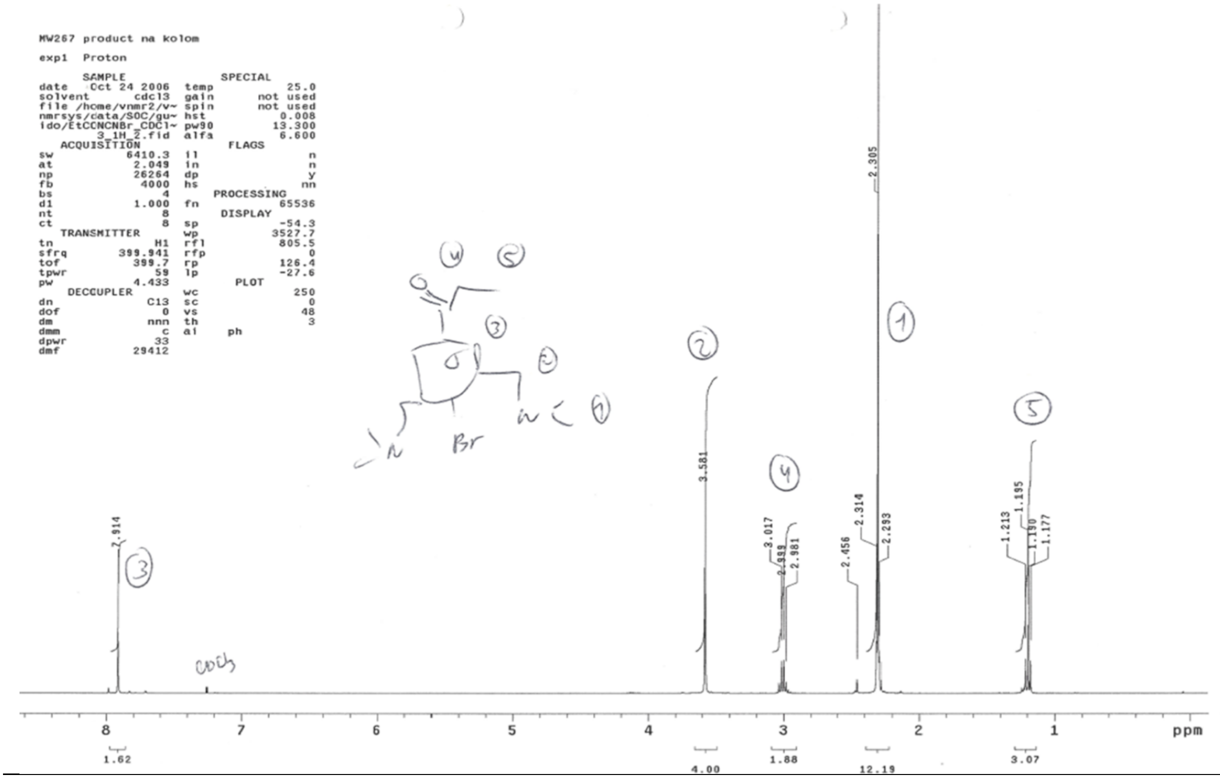

3,5-Bis[(dimethylamino)methyl]-4-bromo-propiophenone (20). $^{13}C\{^{1}H\}$ NMR (101 MHz, $CDCl_3$):

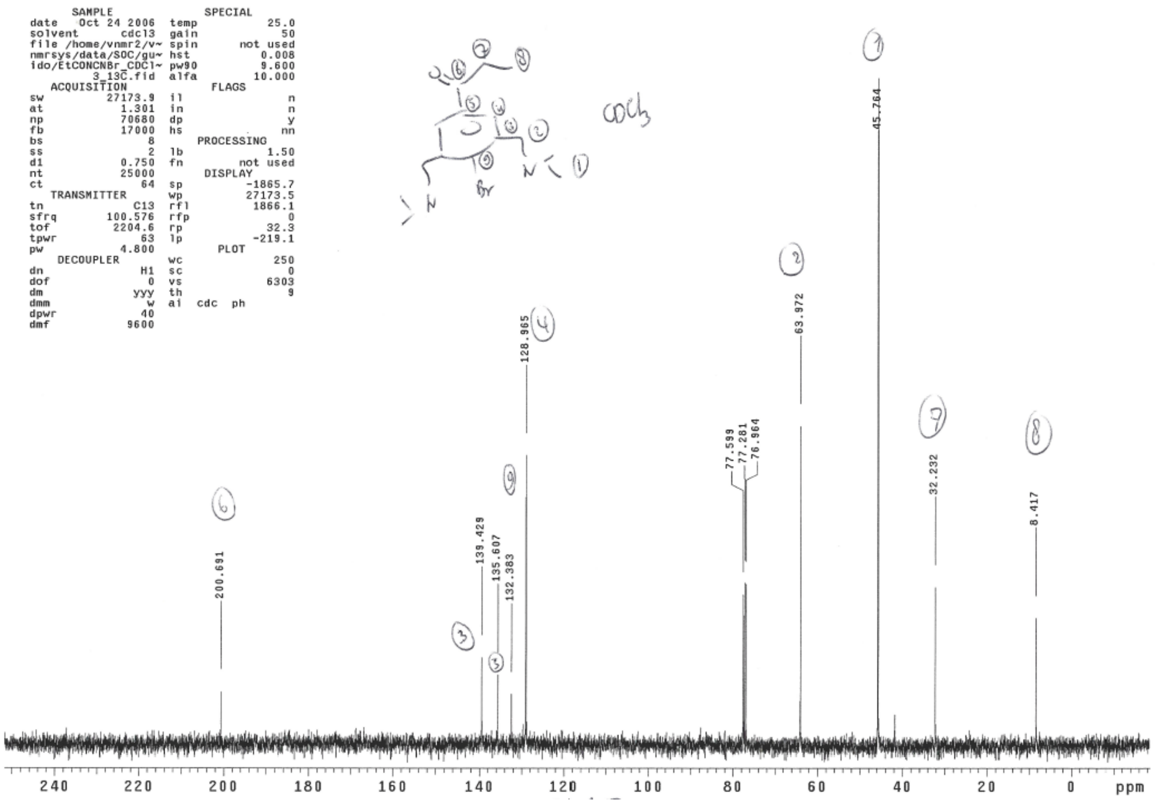

3,5-Bis[(dimethylamino)methyl]-4-bromo-propiophenone (20). IR (ATR):

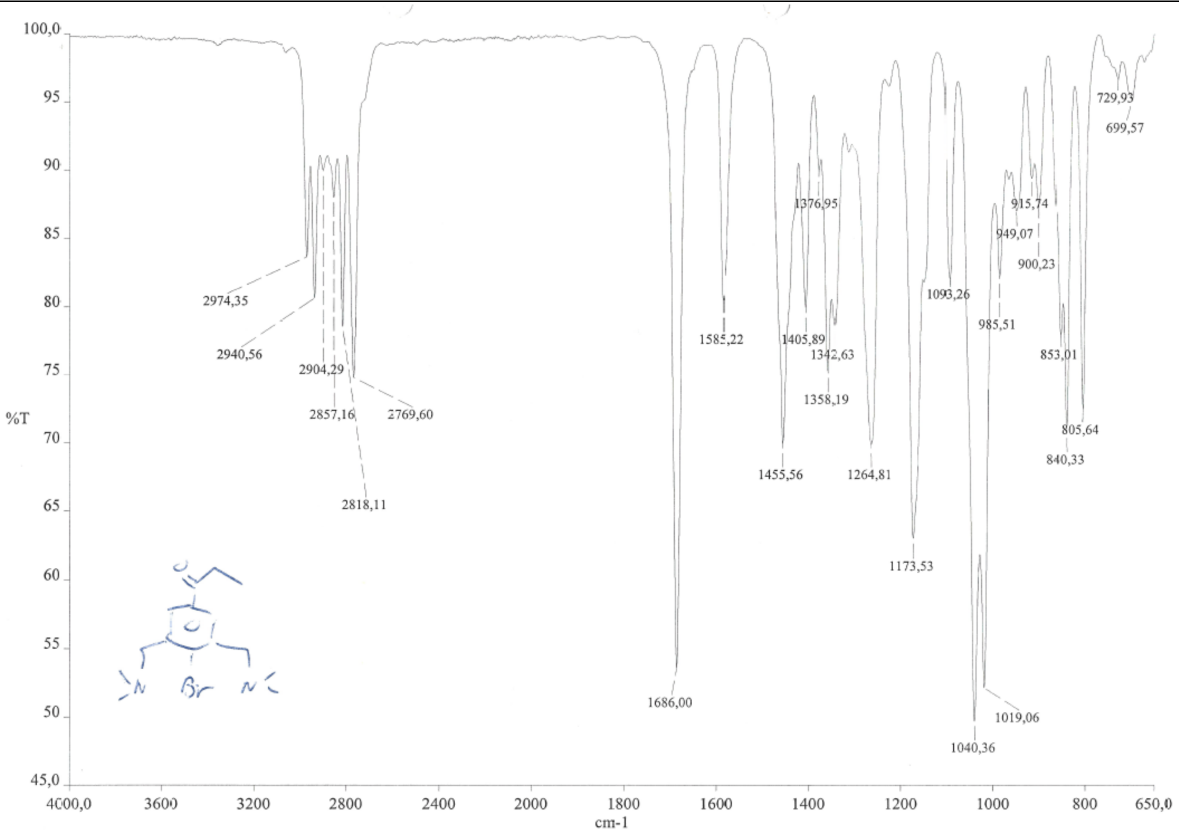

[PtBr(NCN-C(O)Et-4)] (21). ^{1}H NMR (300 MHz, $CDCl_3$):

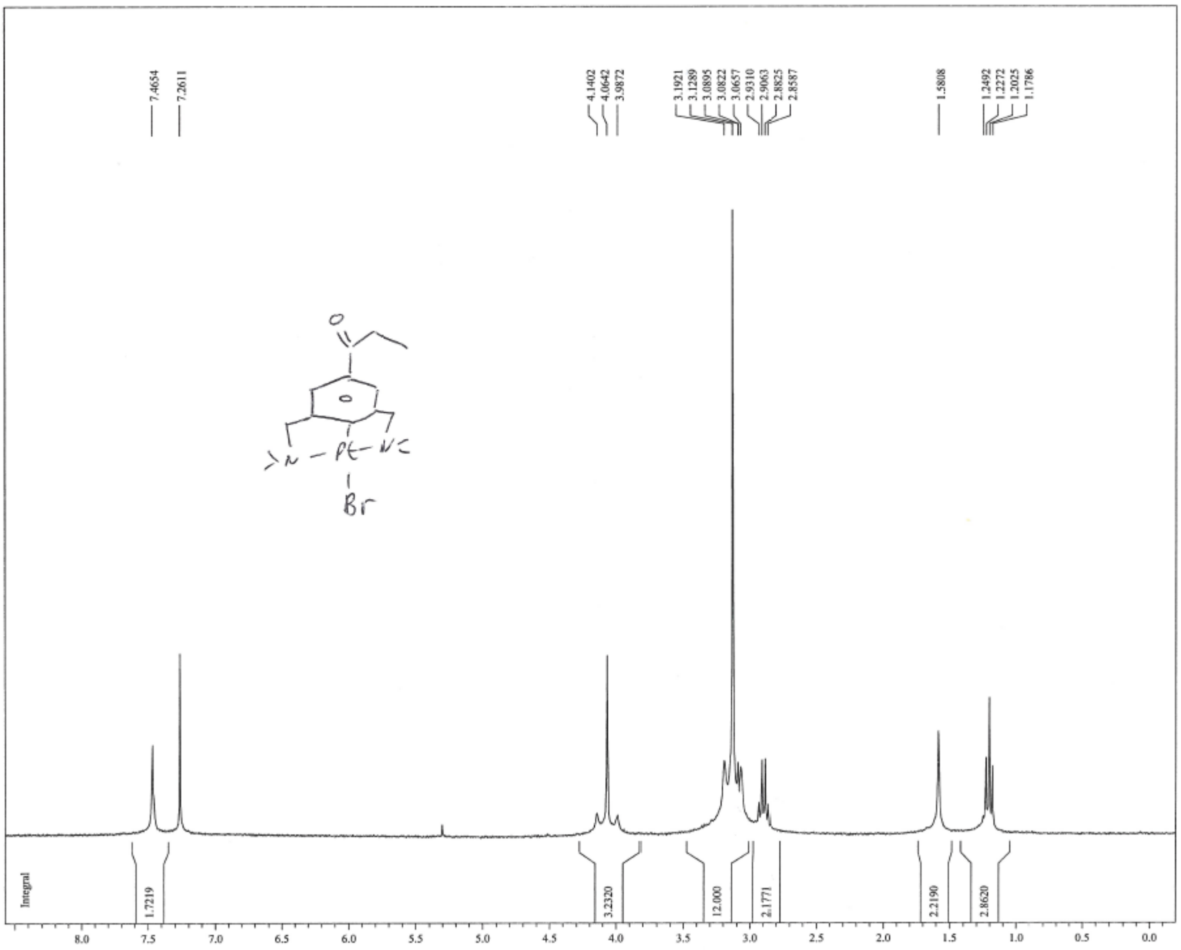

[PtBr(NCN-C(O)Et-4)] (21). $^{13}C\{^1H\}$ NMR (75 MHz, $CDCl_3$):

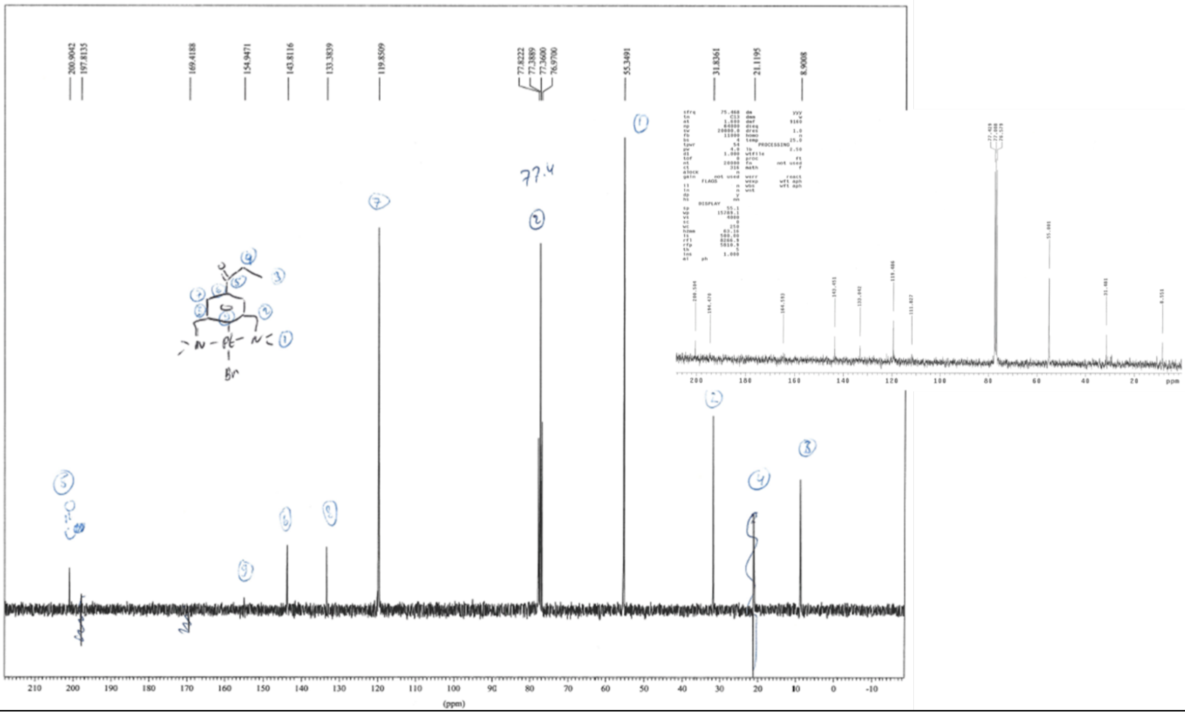

[PtBr(NCN-C(O)Et-4)] (21). IR (ATR):

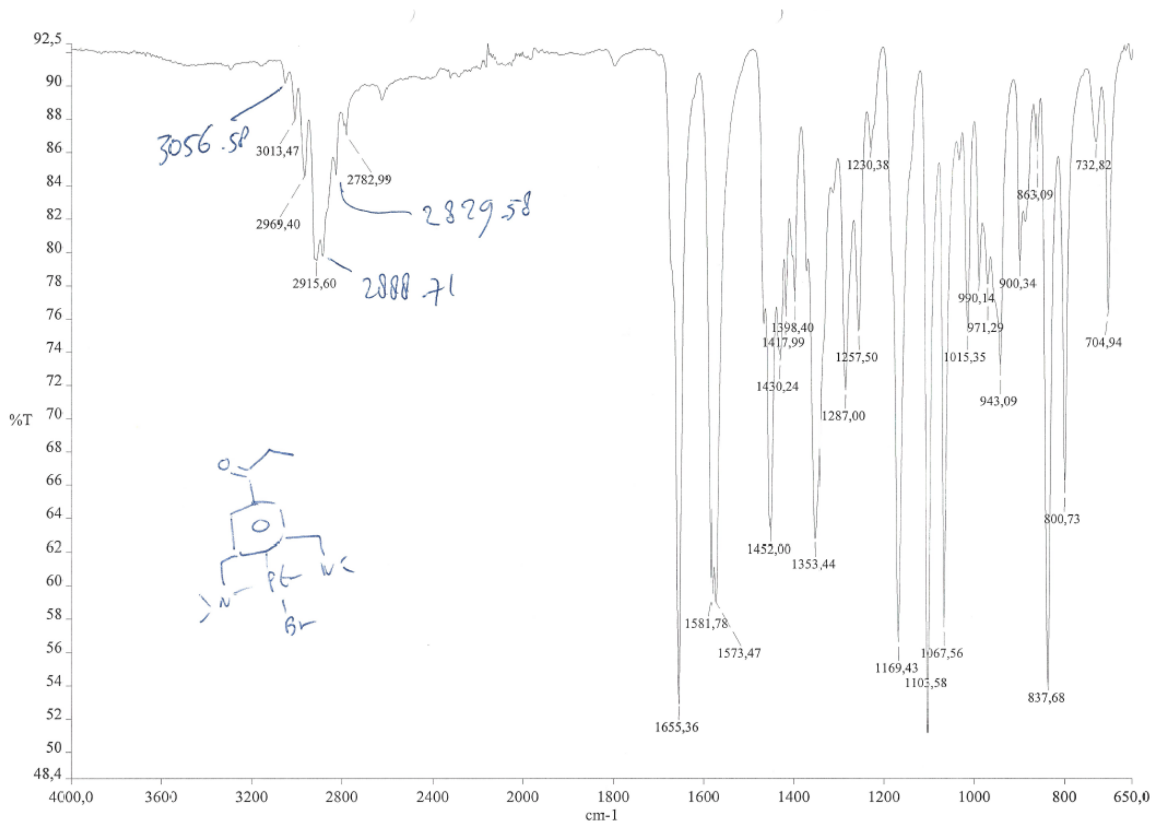

[PtCl(NCN-C(O)Et-4)] (22). ^1H NMR (300 MHz, $CDCl_3$):

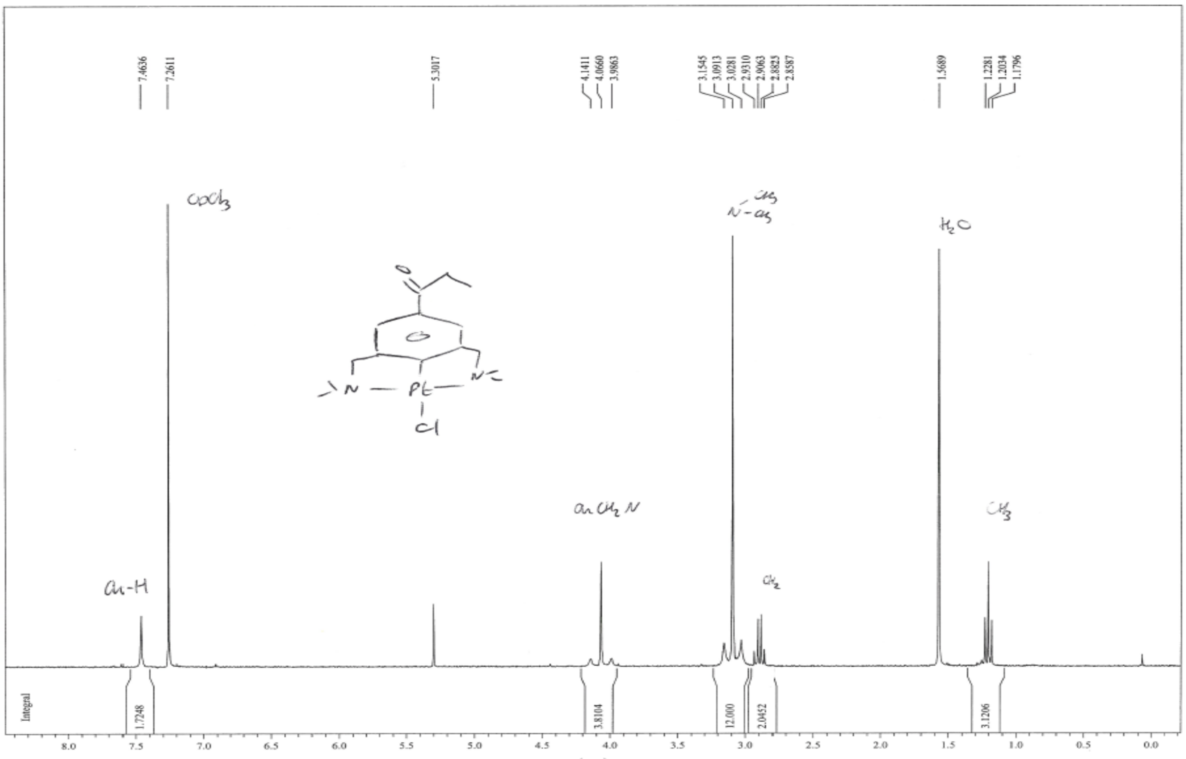

[PtCl(NCN-C(O)Et-4)] (22). $^{13}C\{^{1}H\}$ NMR (75 MHz, $CDCl_3$):

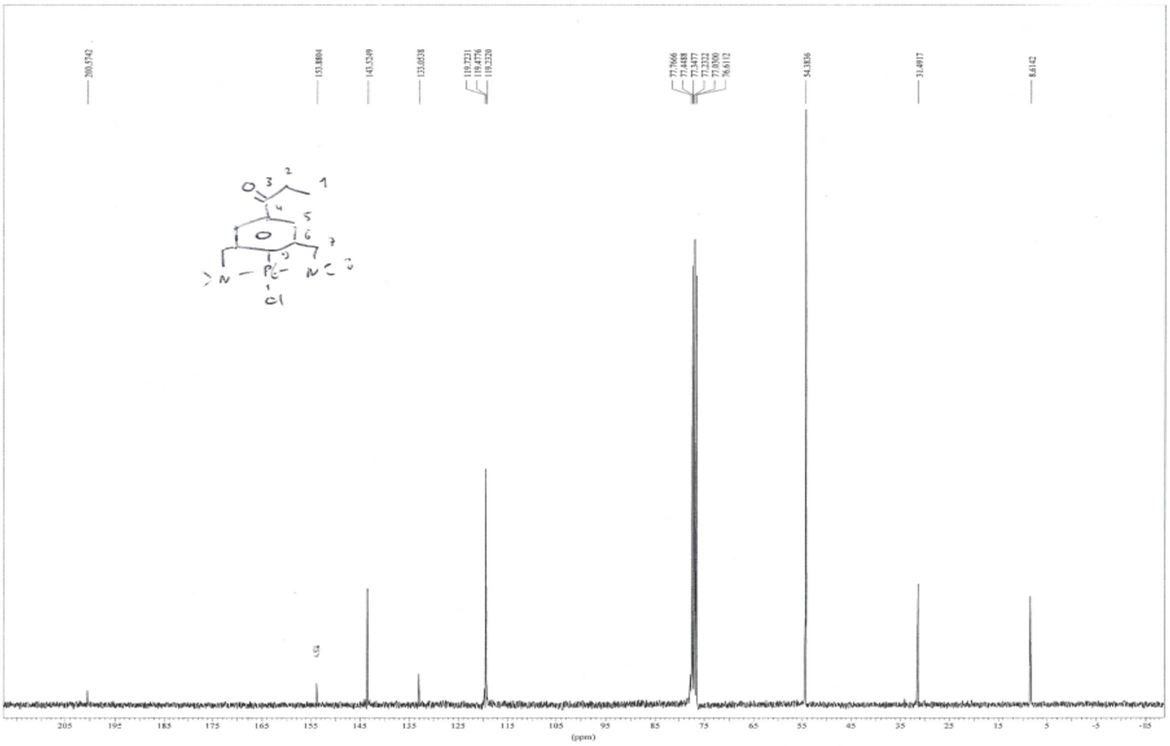

[PtCl(NCN-C(O)Et-4)] (22). IR (ATR):

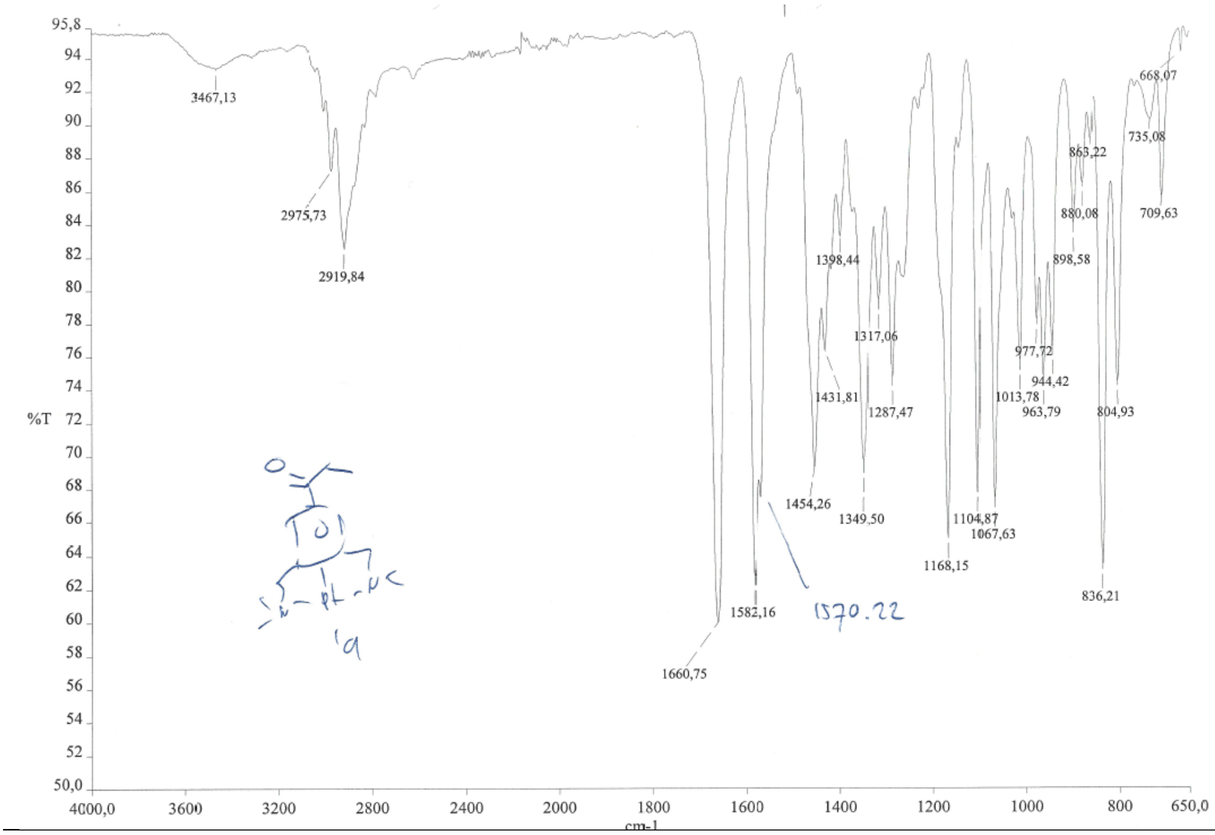

Attempted synthesis of 1-(1,1-Diphenyl-1-propenyl)-4-bromo-3,5-bis[(dimethylamino)methyl]benzene (9); formation of / 1-(1,1-Diphenyl- 1-propenyl)-3,5-bis[(dimethylamino)methyl]benzene (10). For **10**: ^{1}H NMR (300 MHz, $CDCl_3$):

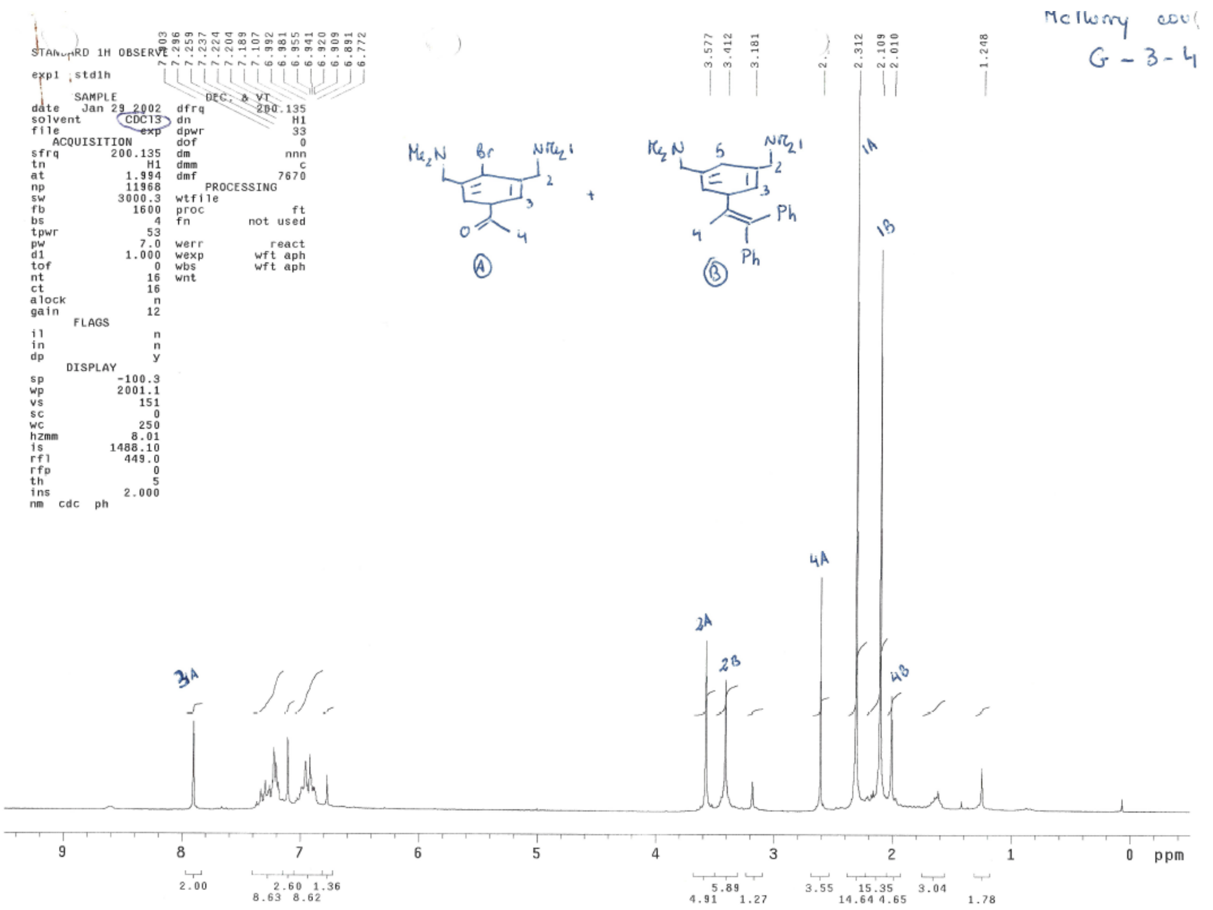

Attempted synthesis of 3,3',5,5'-tetra(dimethylamino)methyl-4,4'-bisplatinumbromide-stilbene (12); formation of 3,3',5,5'- tetra(dimethylaminomethyl)-4,4'-bisplatinumhalide-benzophenone (13), (halide = Br and Cl). ^{1}H NMR (400 MHz, CD_2Cl_2):

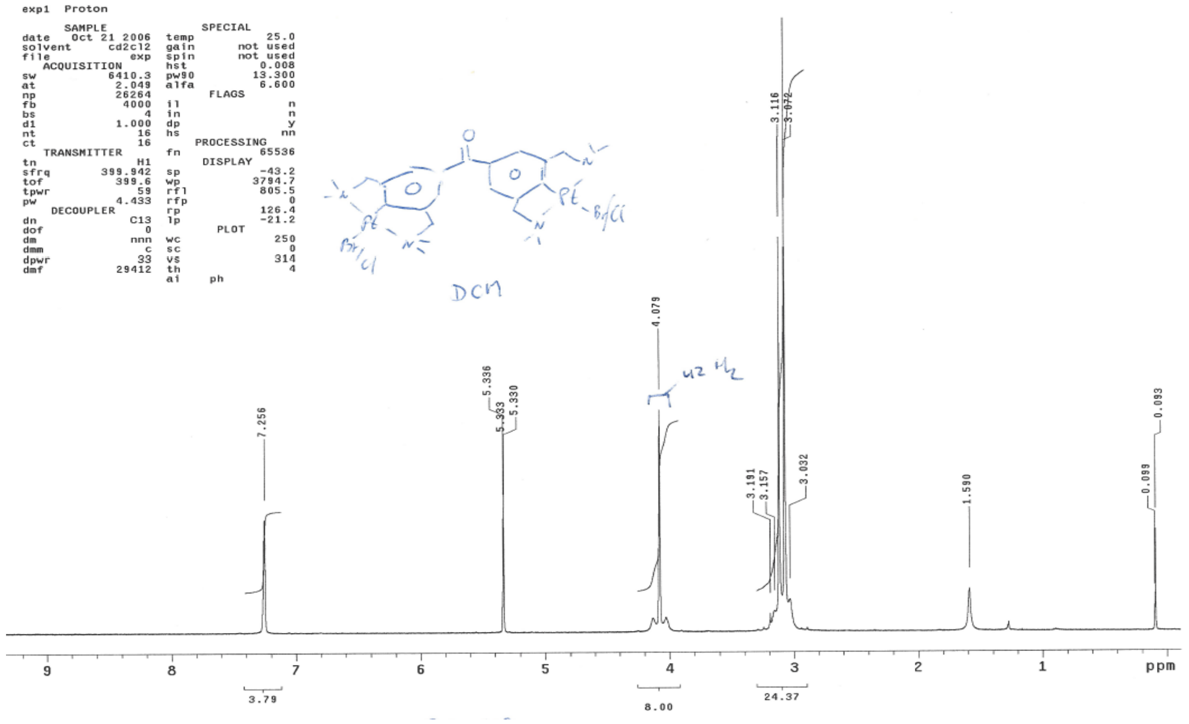

3,3',5,5'-tetra(dimethylaminomethyl)-4,4'-bisplatinumhalide-benzophenone (13), (halide = Br and Cl). $^{13}C\{^{1}H\}$ NMR (101 MHz, CD_2Cl_2):

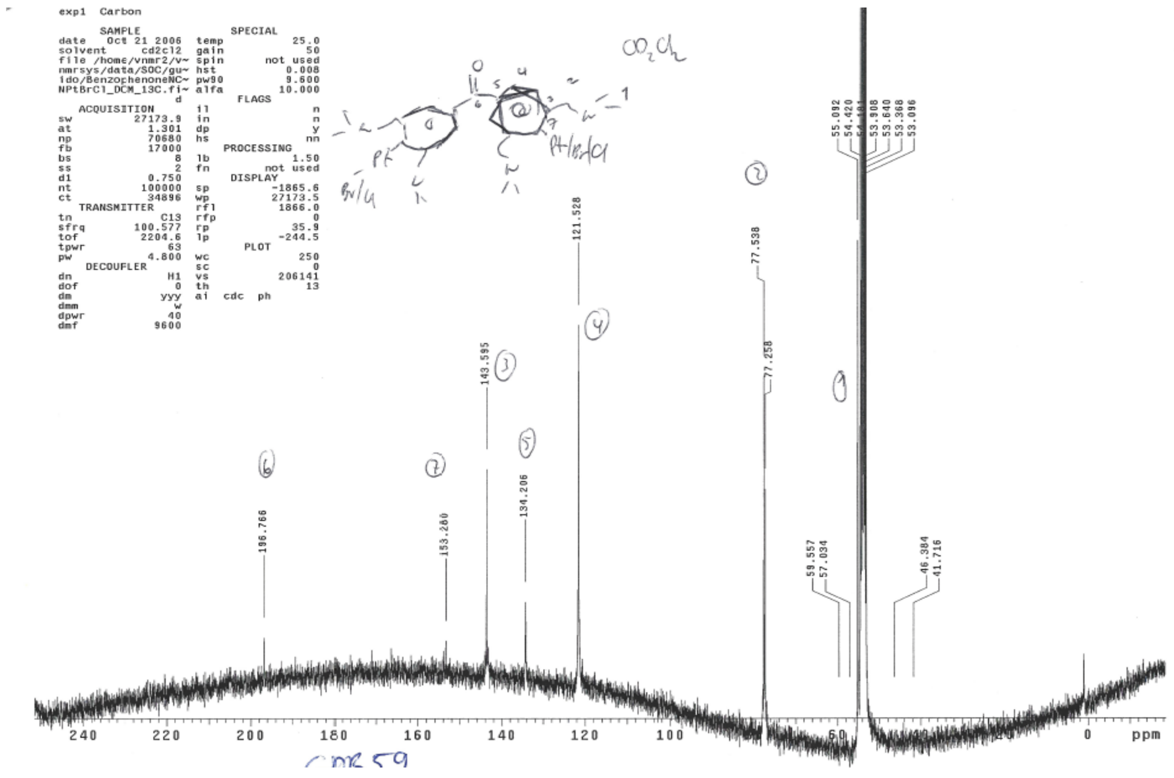

3,3',5,5'-tetra(dimethylaminomethyl)-4,4'-bisplatinumhalide-benzophenone (13), (halide = Br and Cl). $^{195}Pt\{^{1}H\}$ NMR (64 MHz, CD_2Cl_2):

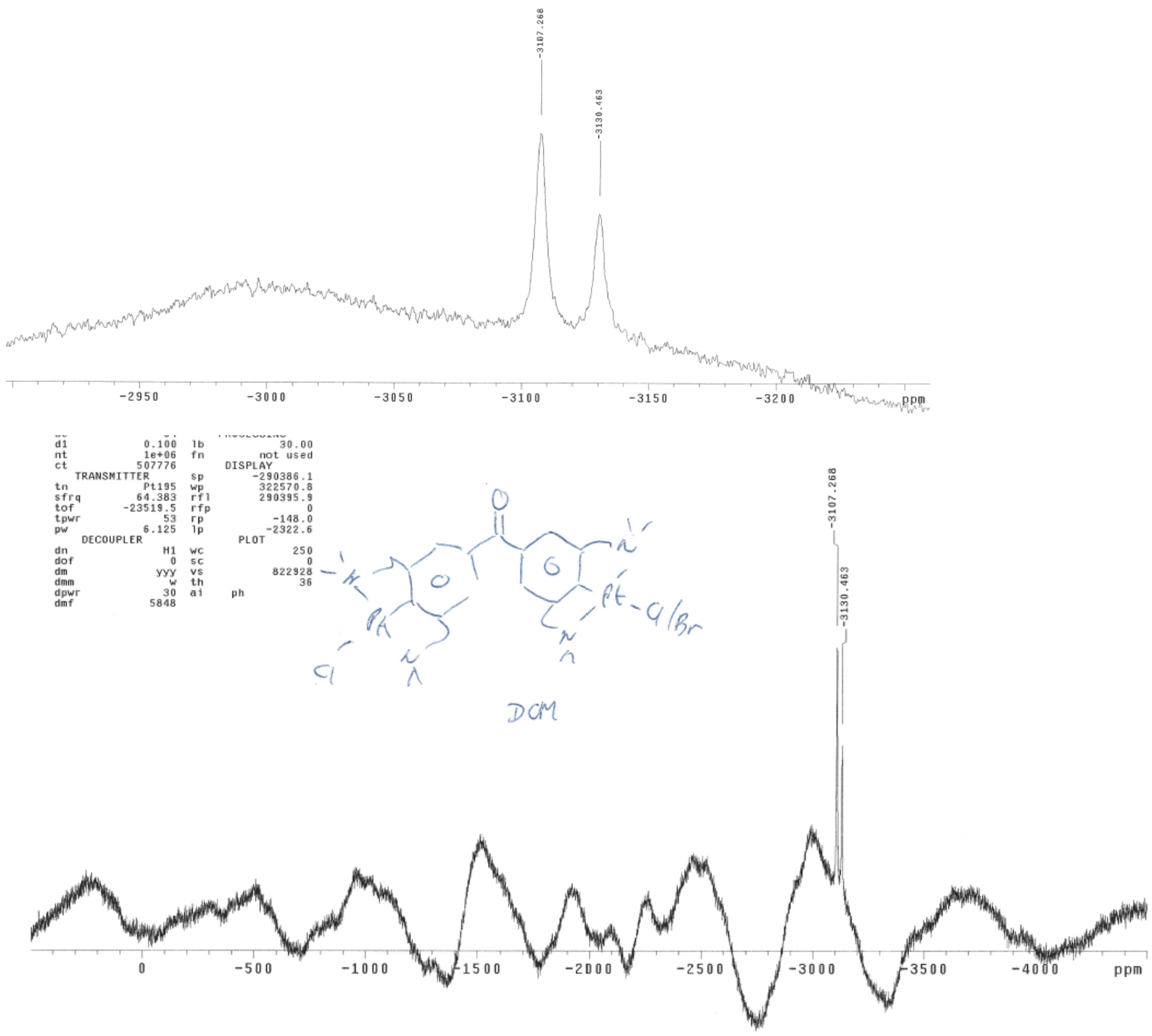

3,3',5,5'-tetra(dimethylaminomethyl)-4,4'-bisplatinumhalide-benzophenone (13), **(halide = Br and Cl)**. IR (ATR):

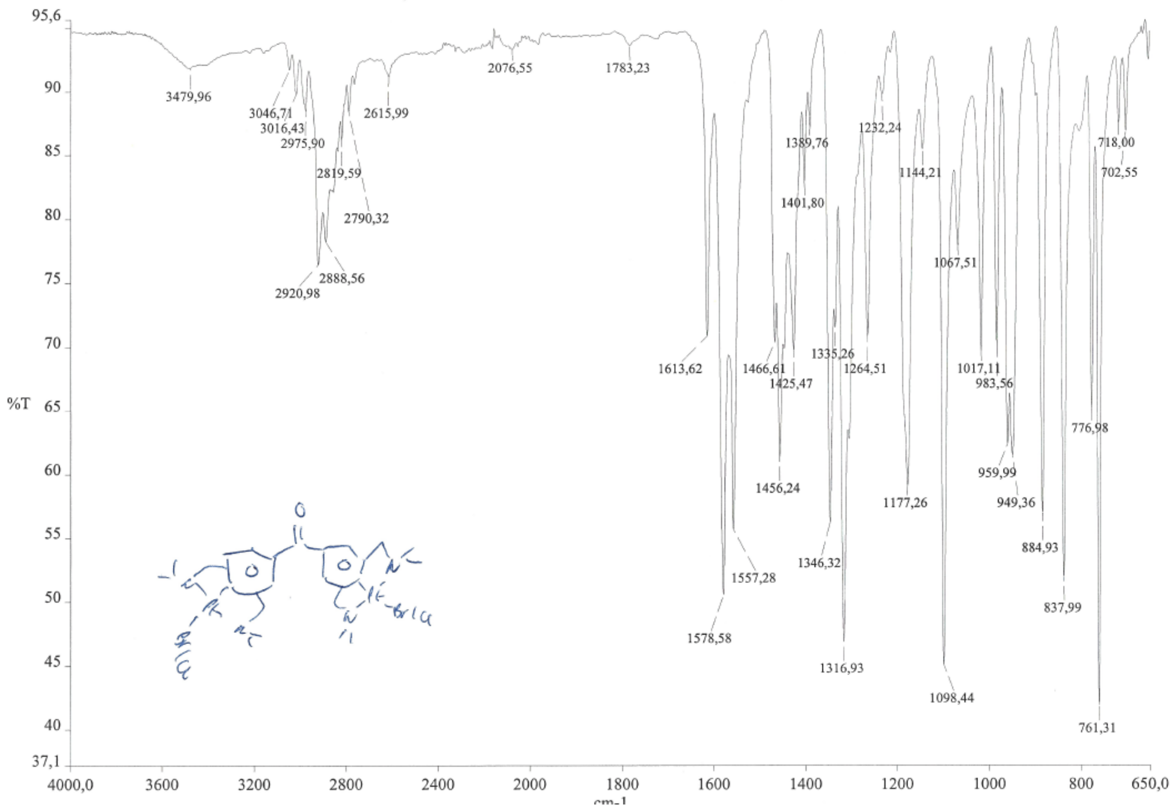

1-(2-(1-[(4-dimethylaminoethoxy)phenyl]-1-phenyl-1-butenyl))-4-PtCl-3,5-bis[(dimethylamino)methyl])benzene (5). For **5a**: ^{1}H NMR (300 MHz, $CDCl_3$):

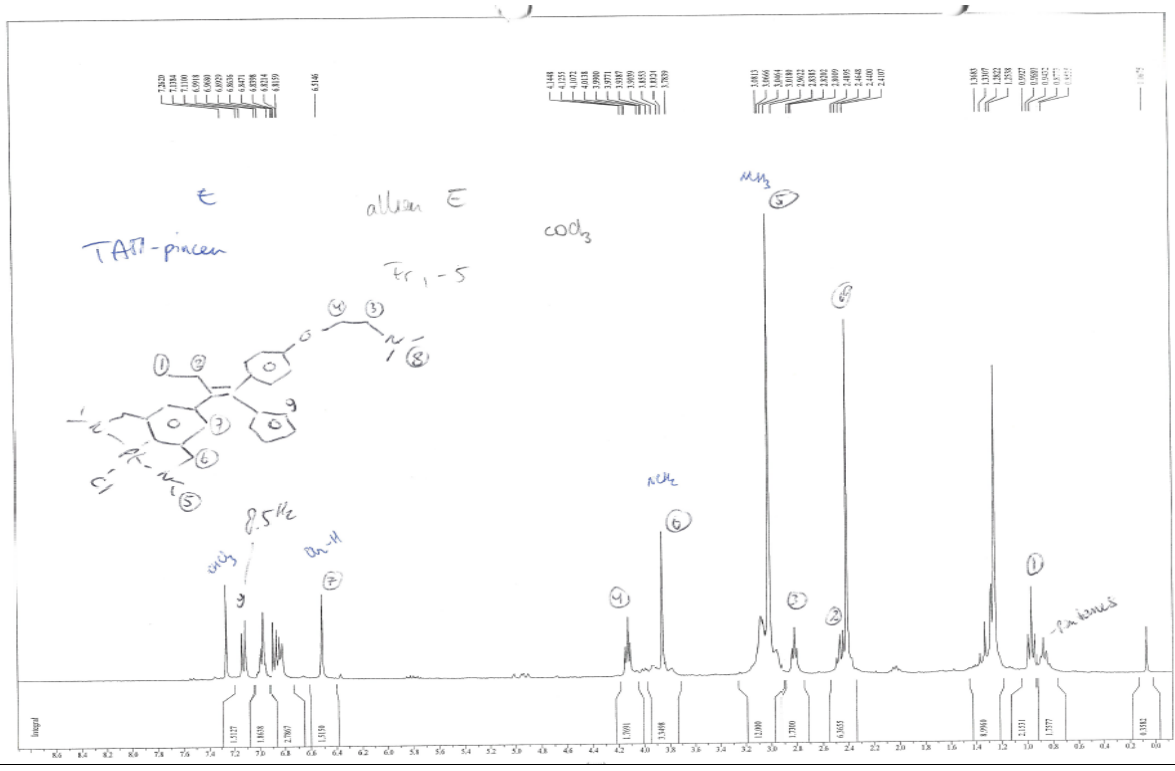

1-(2-(1-[(4-dimethylaminoethoxy)phenyl]-1-phenyl-1-butenyl))-4-PtCl-3,5-bis[(dimethylamino)methyl])benzene (5). For **5a**: $^{13}C\{^{1}H\}$ NMR (75 MHz, $CDCl_3$):

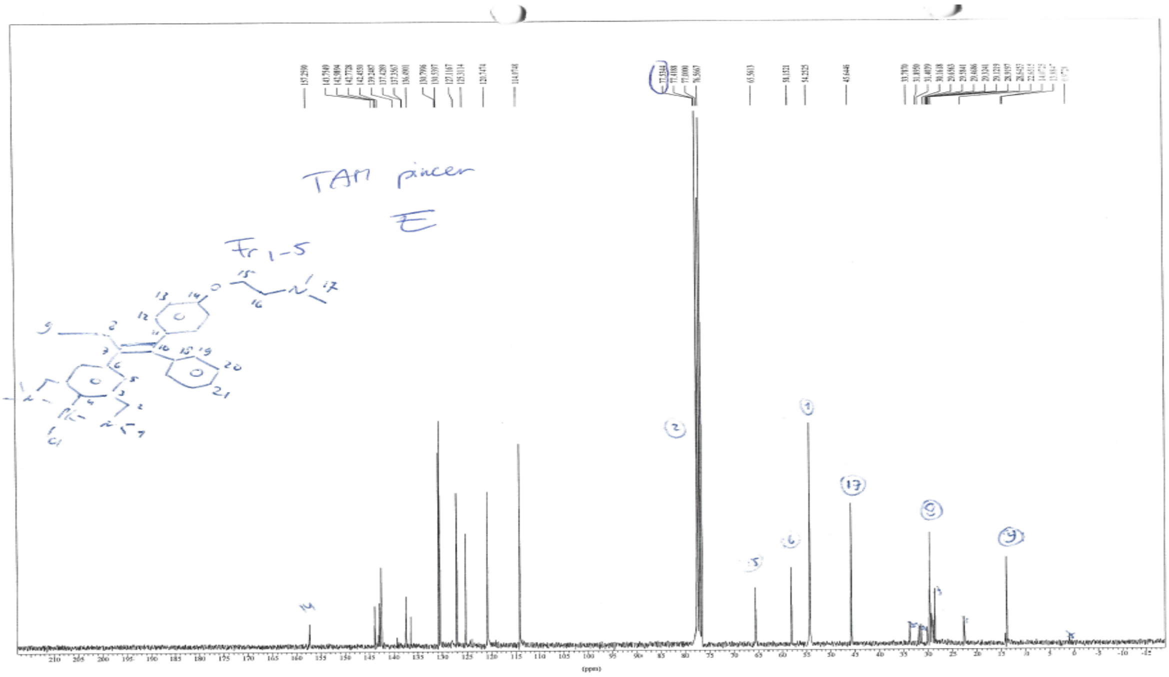

1-(2-(1-[(4-dimethylaminoethoxy)phenyl]-1-phenyl-1-butenyl))-4-PtCl-3,5-bis[(dimethylamino)methyl])benzene (5). For **5a**: $^{195}Pt\{^{1}H\}$ NMR (64 MHz, $CDCl_3$):

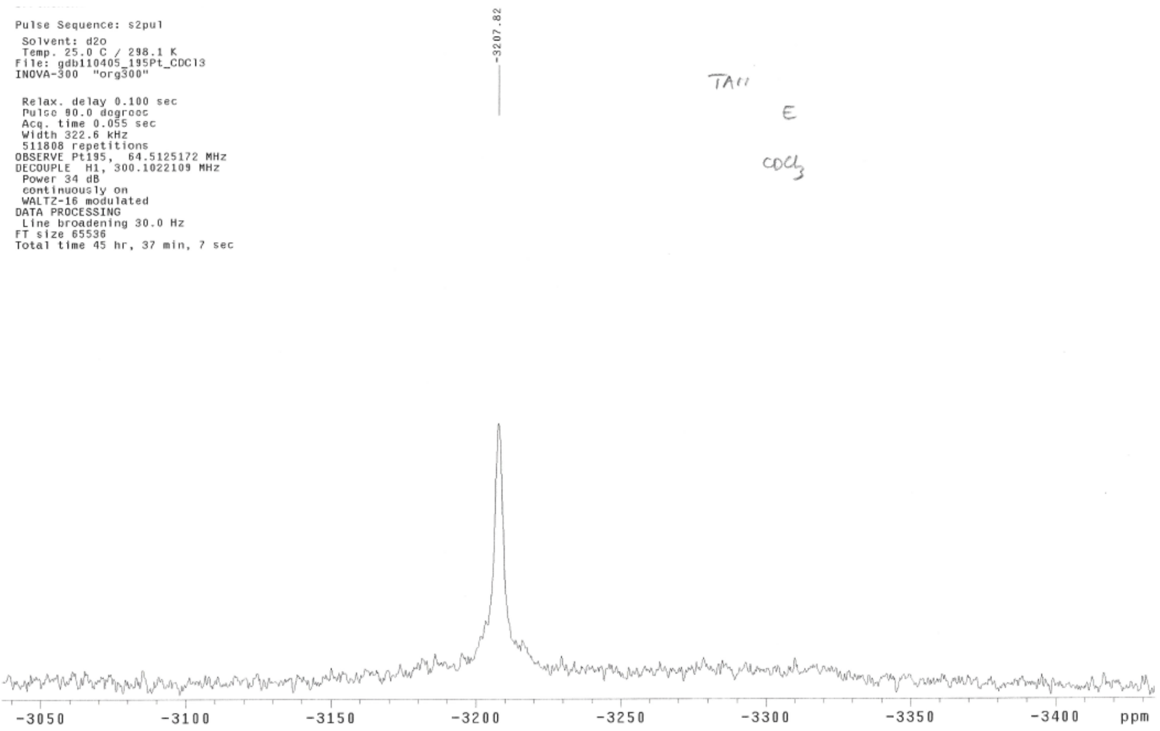

1-(2-(1-[(4-dimethylaminoethoxy)phenyl]-1-phenyl-1-butenyl))-4-PtCl-3,5-bis[(dimethylamino)methyl])benzene (5). For **5a**: IR (ATR):

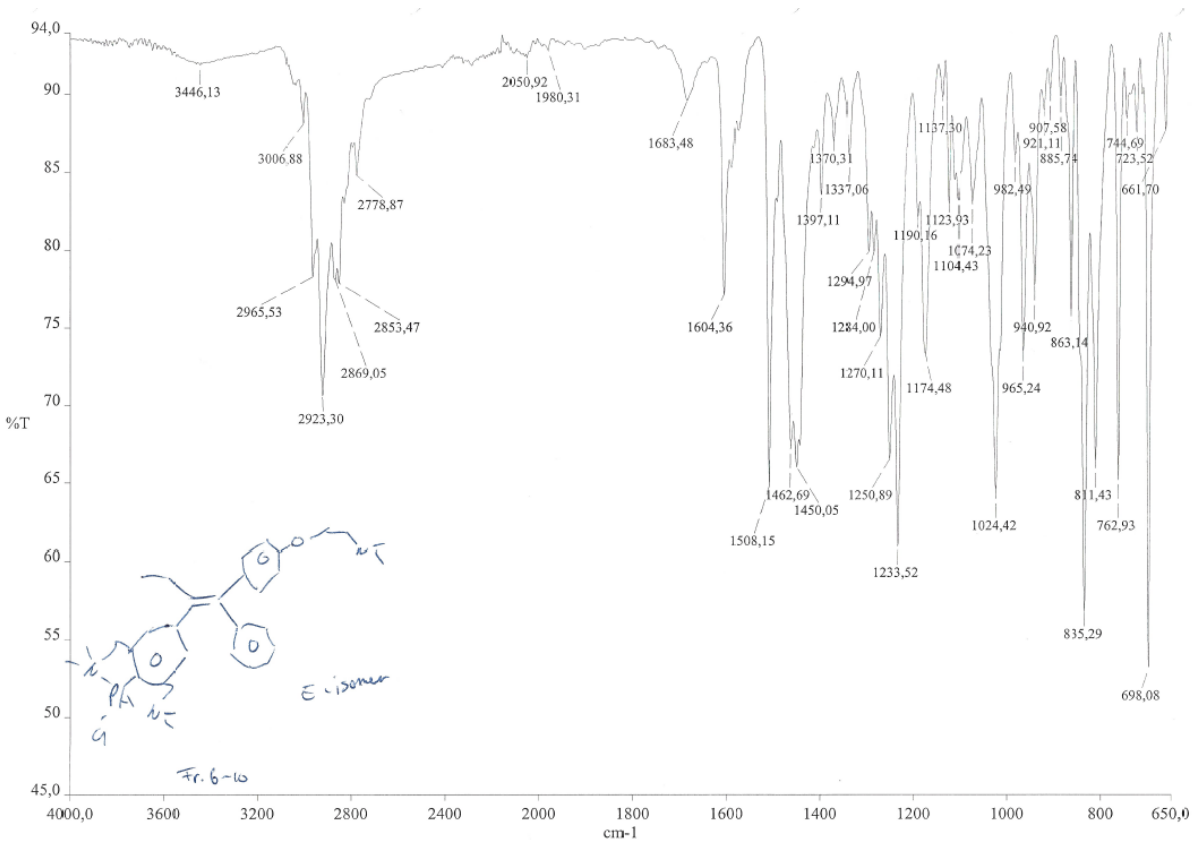

1-(2-(1-[(4-dimethylaminoethoxy)phenyl]-1-phenyl-1-butenyl))-4-PtCl-3,5-bis[(dimethylamino)methyl])benzene (5). For **5b**: ^{1}H NMR (300 MHz, $CDCl_3$):

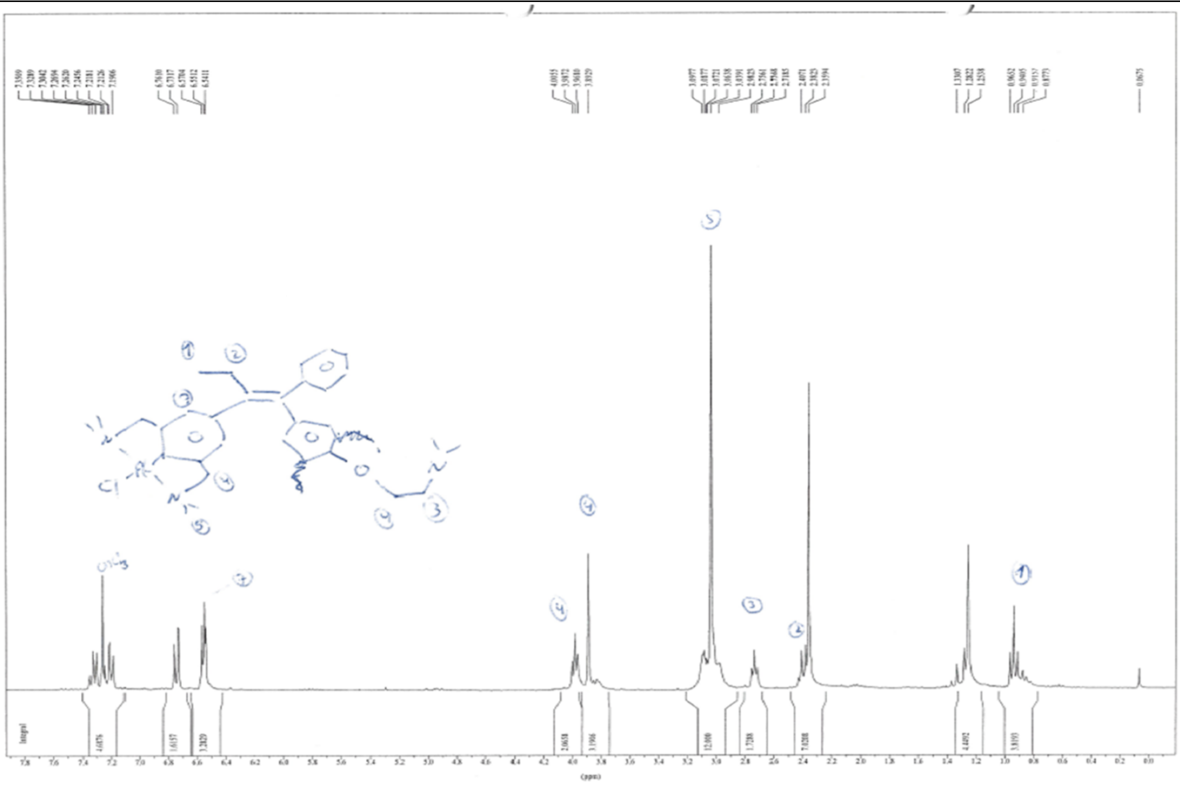

1-(2-(1-[(4-dimethylaminoethoxy)phenyl]-1-phenyl-1-butenyl))-4-PtCl-3,5-bis[(dimethylamino)methyl])benzene (5). For **5b**: $^{13}C\{^{1}H\}$ NMR (75 MHz, $CDCl_3$):

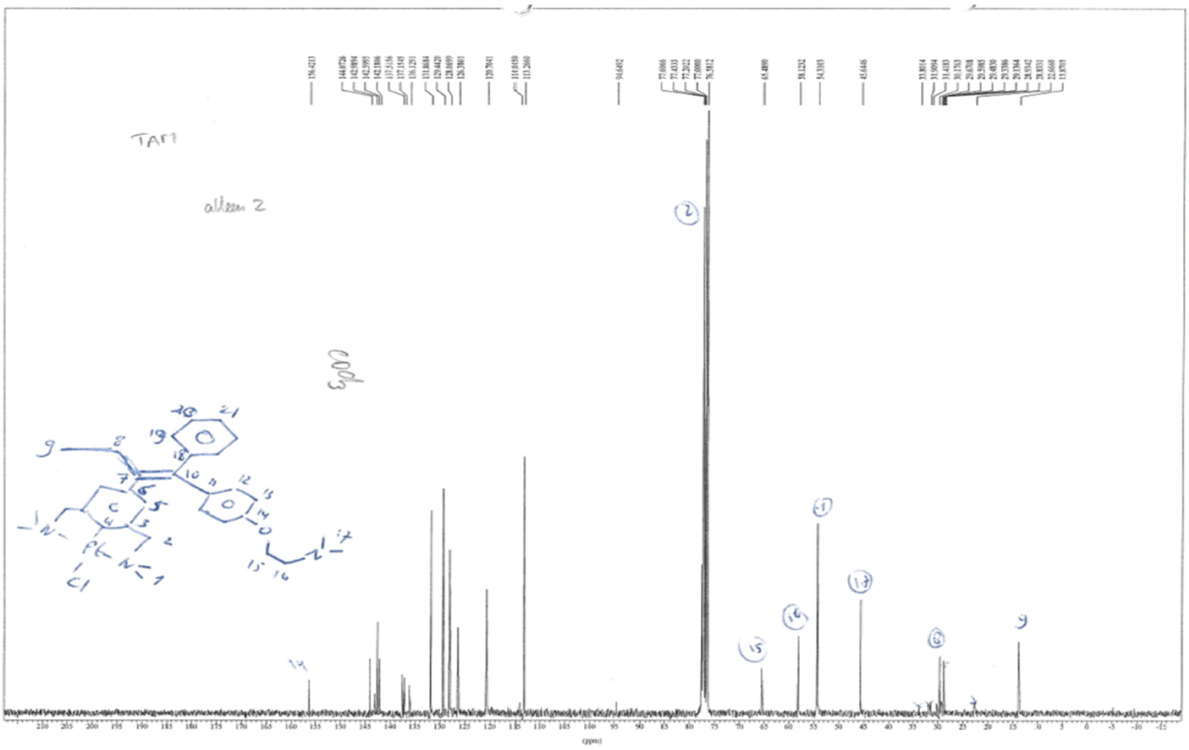

1-(2-(1-[(4-dimethylaminoethoxy)phenyl]-1-phenyl-1-butenyl))-4-PtCl-3,5-bis[(dimethylamino)methyl])benzene (5). For **5b**: $^{195}Pt\{^{1}H\}$ NMR (64 MHz, $CDCl_3$):

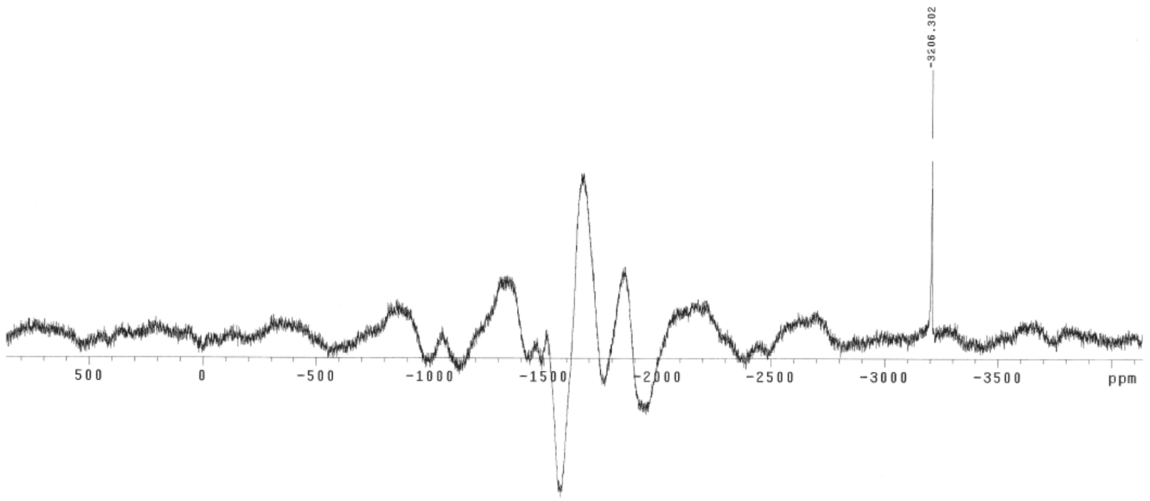

1-(2-(1-[(4-dimethylaminoethoxy)phenyl]-1-phenyl-1-butenyl))-4-PtCl-3,5-bis[(dimethylamino)methyl])benzene (5). For **5b**: IR (ATR):

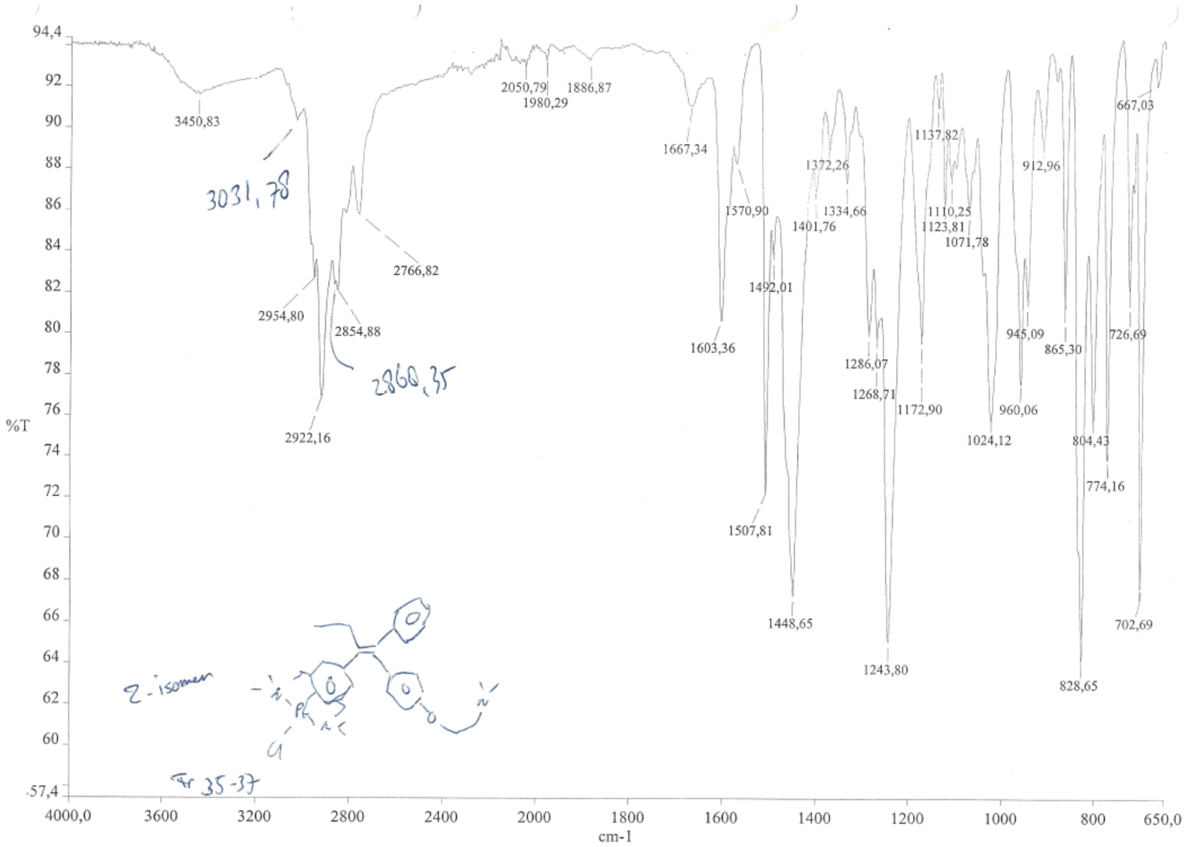

1-(4-[2-(dimethylamino)ethoxy]phenyl)-1-(4-trimethylacetoxyphenyl)-2-(NCN-PtCl)but-1-ene (26). E/Z mixture 1H NMR (400 MHz, C6D6):

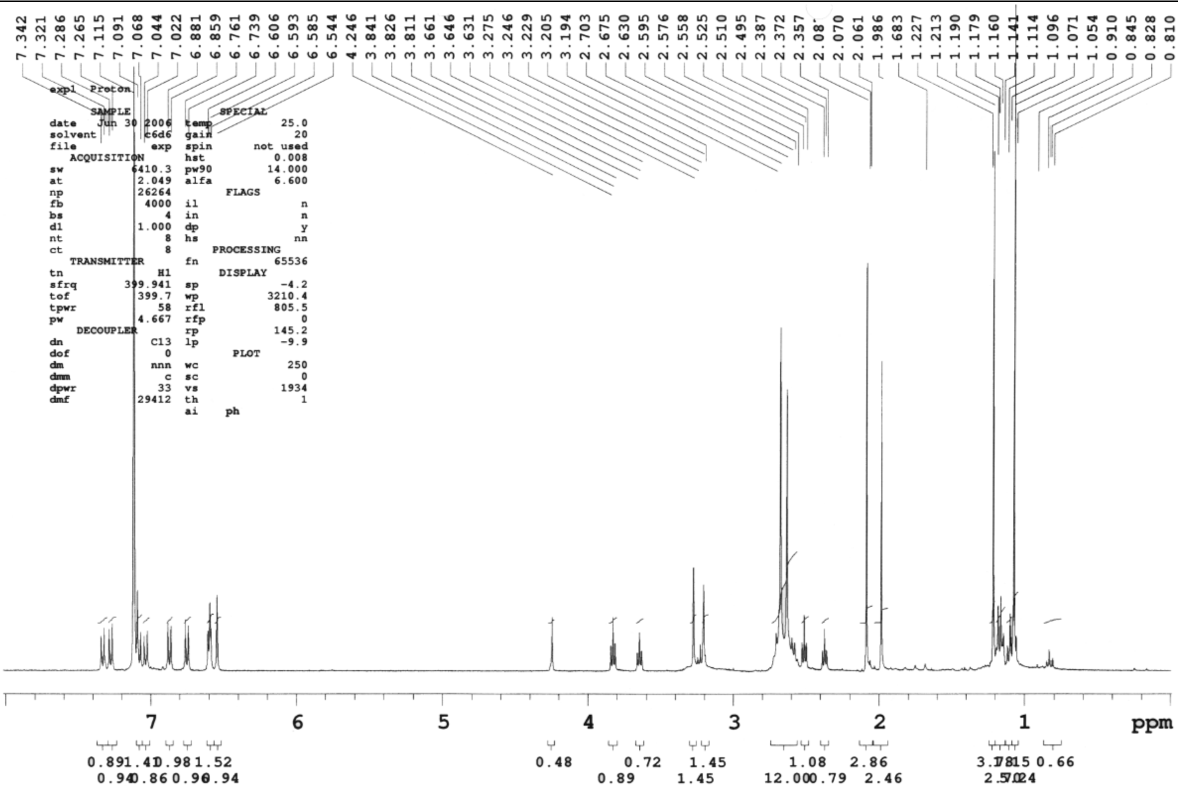

1-(4-[2-(dimethylamino)ethoxy]phenyl)-1-(4-trimethylacetoxyphenyl)-2-(NCN-PtCl)but-1-ene (26). Major isomer 26. 1H NMR (400 MHz, C6D6):

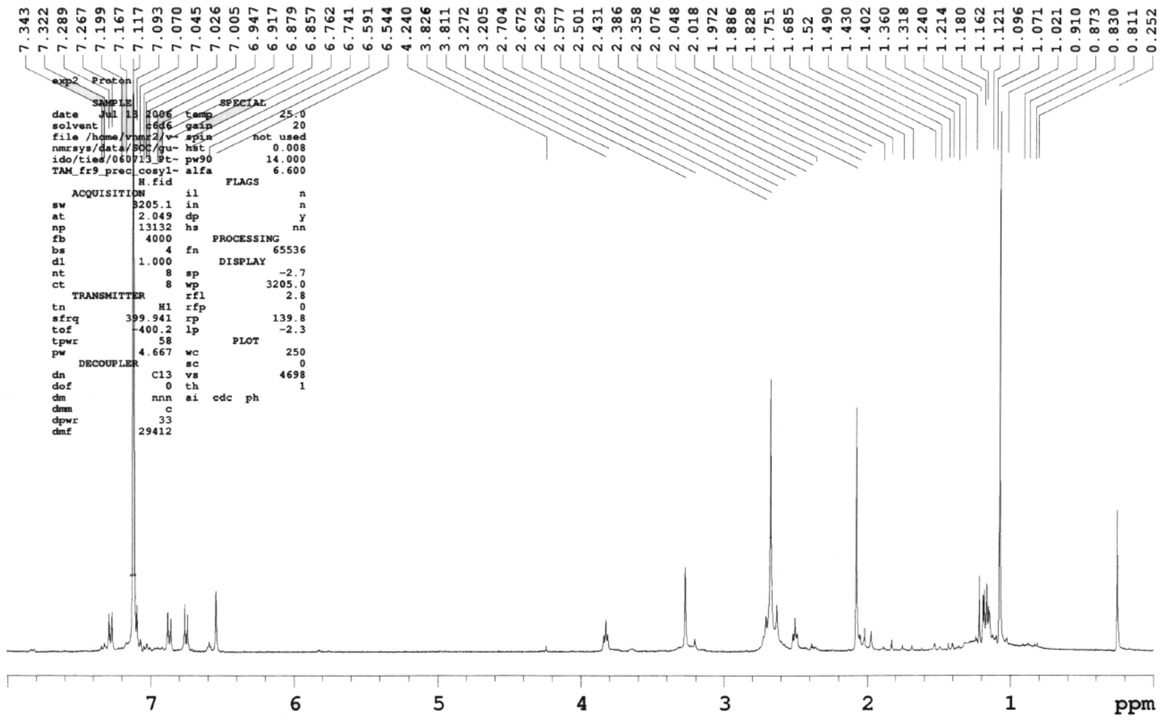

1-(4-[2-(dimethylamino)ethoxy]phenyl)-1-(4-trimethylacetoxyphenyl)-2-(NCN-PtCl)but-1-ene (26). Major isomer 26. 195Pt{1H} NMR (64 MHz, CD2Cl2):

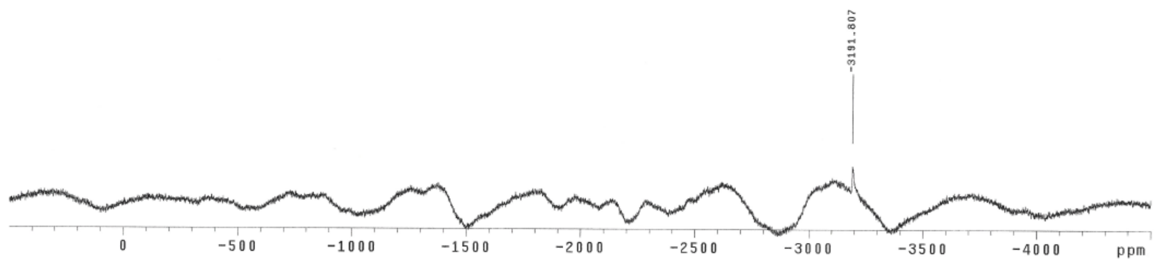

Supporting info COSY, NOESY

1-(4-[2-(dimethylamino)ethoxy]phenyl)-1-(4-trimethylacetoxyphenyl)-2-(NCN-PtCl)but-1-ene (26). Major isomer 26. COSY (400 MHz, C6D6):

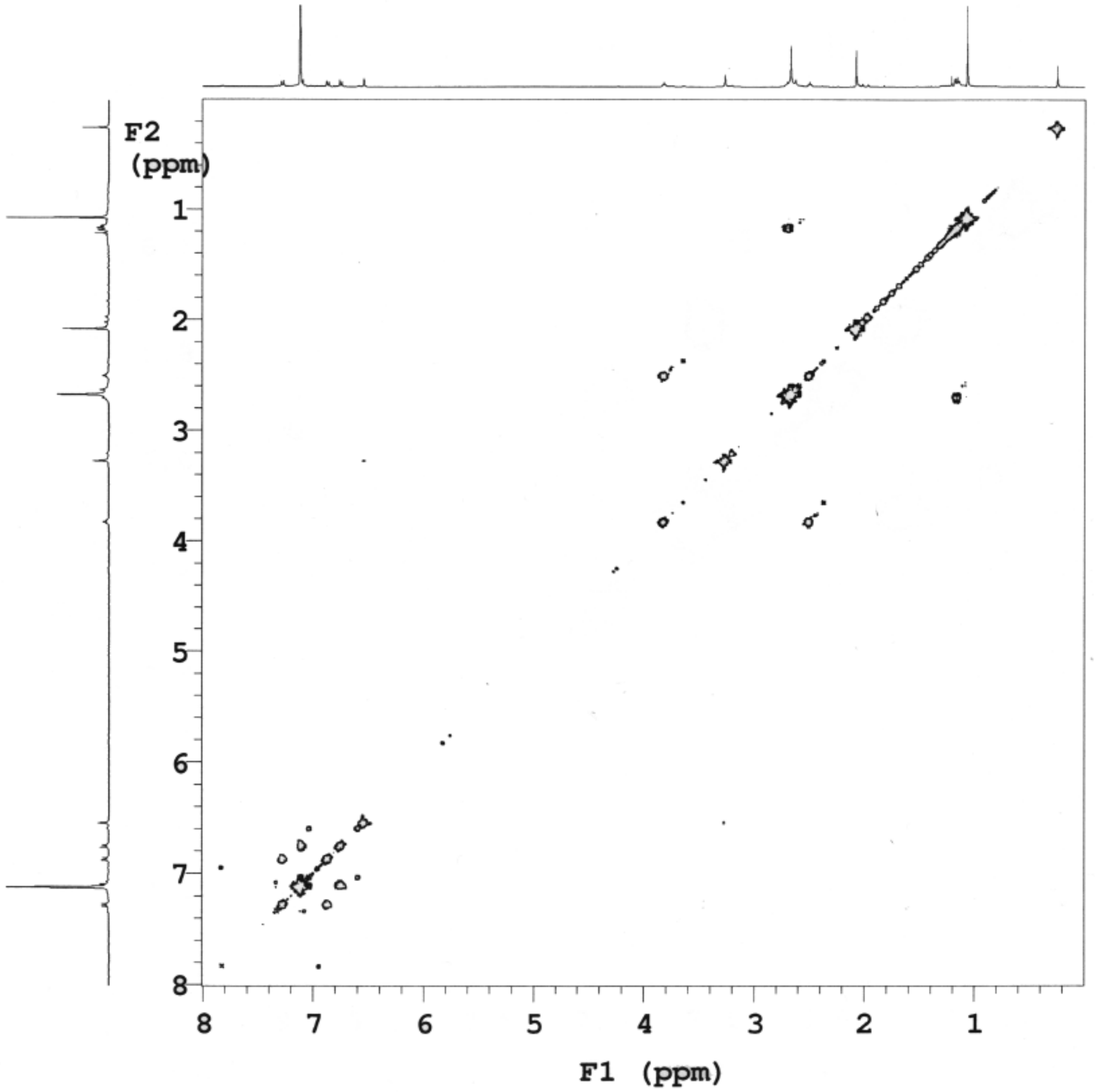
F2
(ppm)
1
2
3
4
5
6
7
8
8
7
6
5
4
3
2
1
F1 (ppm)

1-(4-[2-(dimethylamino)ethoxy]phenyl)-1-(4-trimethylacetoxyphenyl)-2-(NCN-PtCl)but-1-ene (26). Major isomer 26. COSY (400 MHz, C6D6), enlarged from 6.4 to 8 ppm:

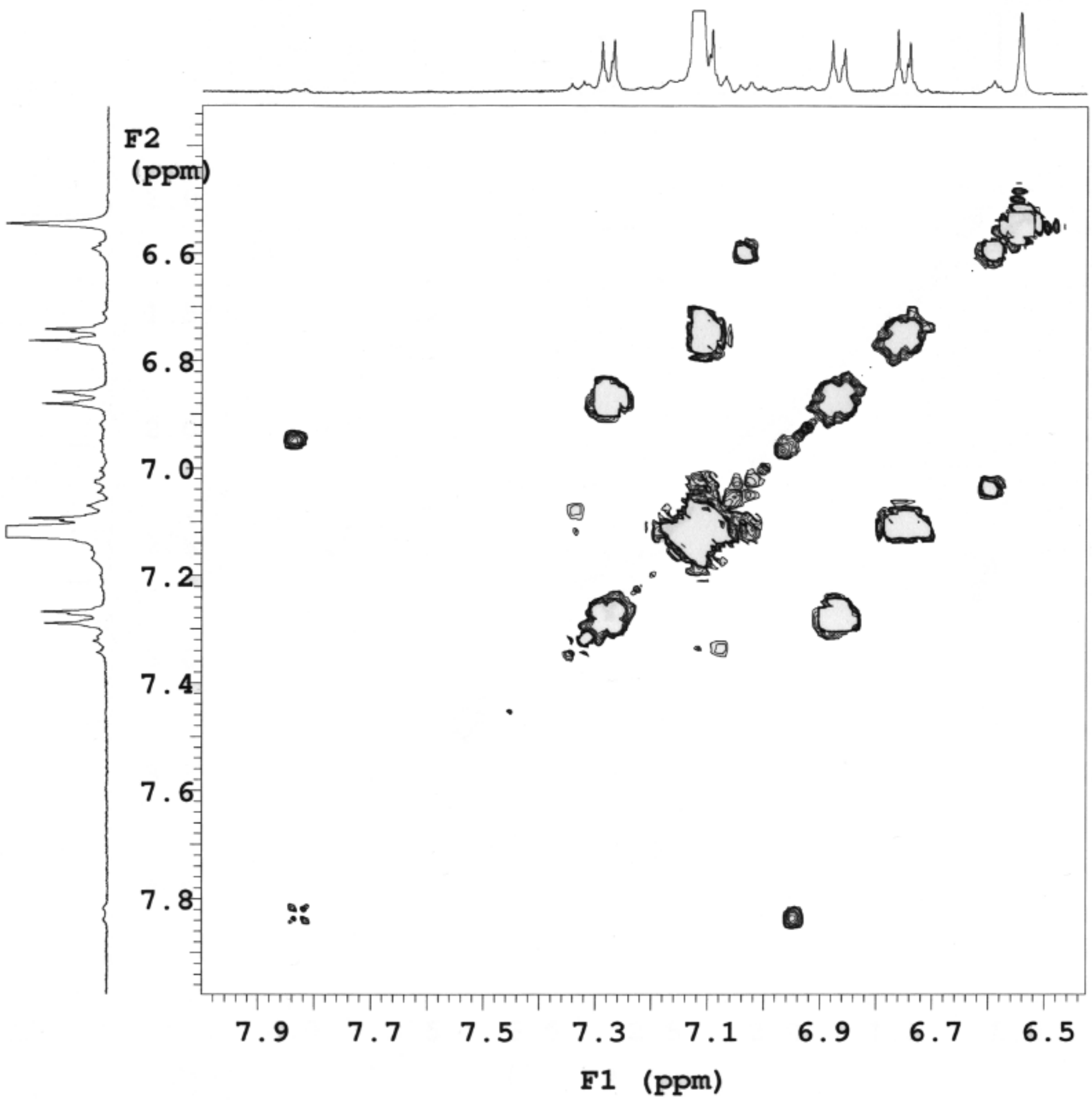

1-(4-[2-(dimethylamino)ethoxy]phenyl)-1-(4-trimethylacetoxyphenyl)-2-(NCN-PtCl)but-1-ene (26). Major isomer 26. COSY (400 MHz, C6D6), enlarged from 0.7 to 4.3 ppm:

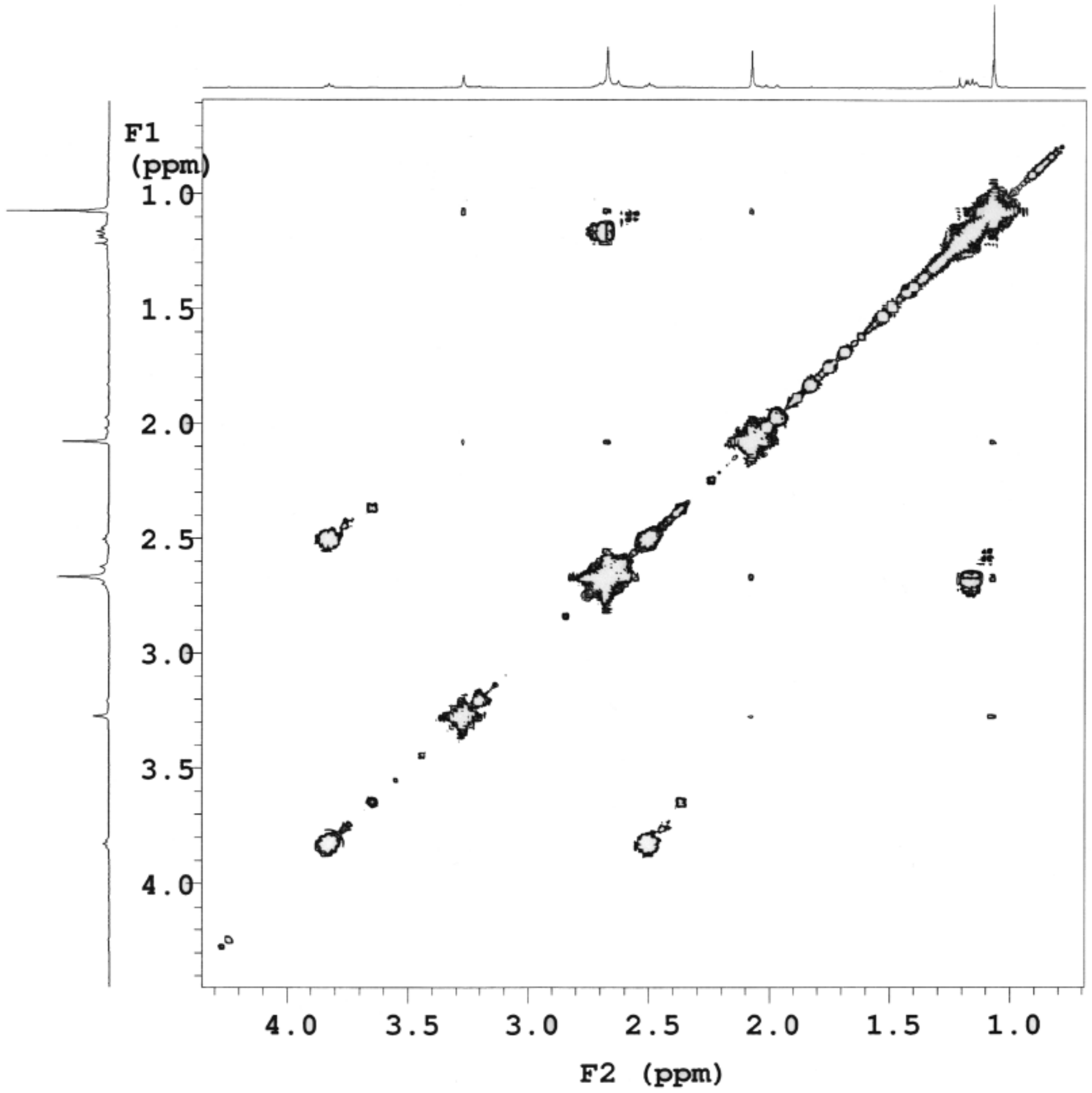

1-(4-[2-(dimethylamino)ethoxy]phenyl)-1-(4-trimethylacetoxyphenyl)-2-(NCN-PtCl)but-1-ene (26). Major isomer 26. NOESY (400 MHz, C6D6):

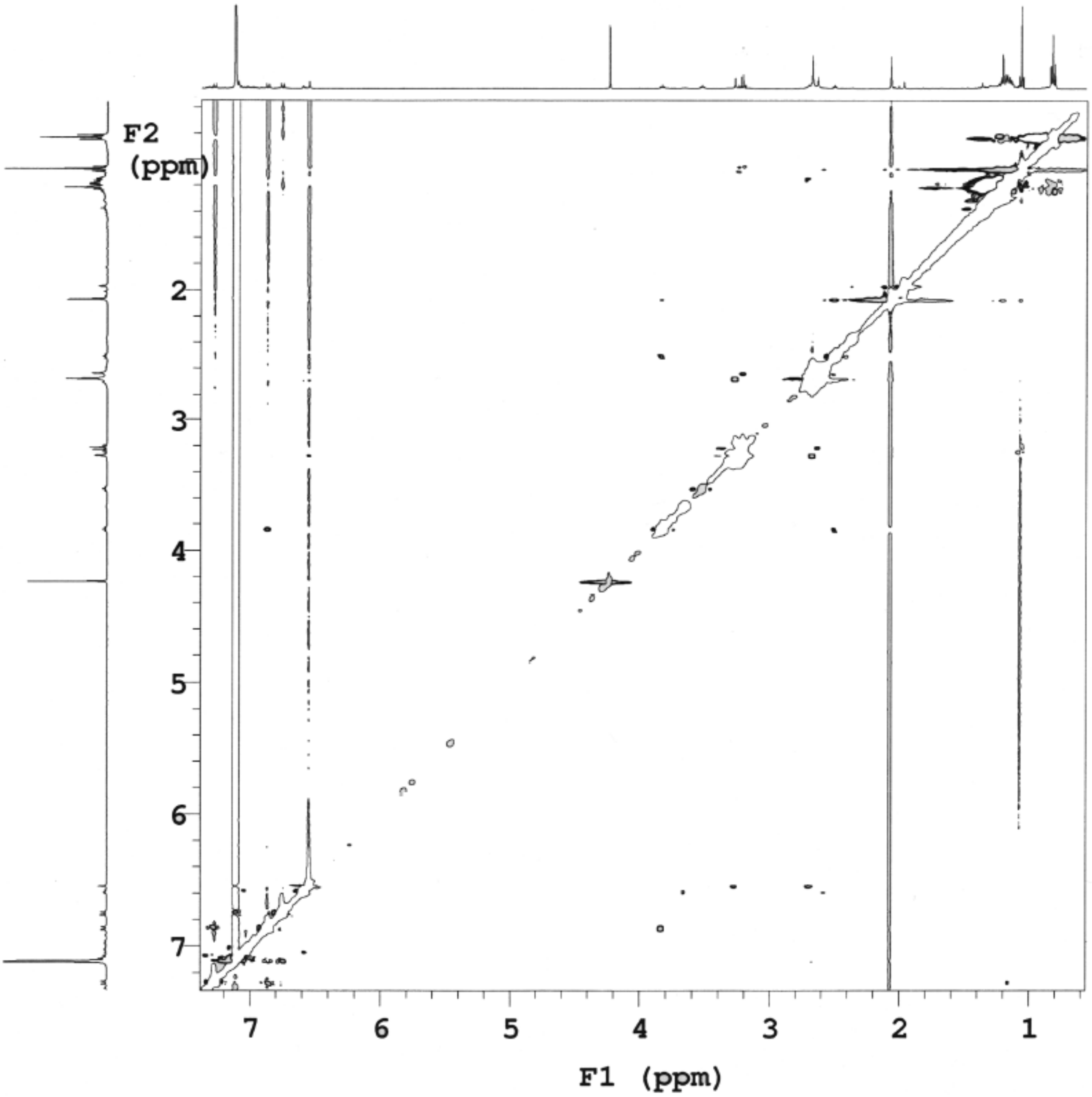

4. X-ray data and Files

Table S2. Selected bond lengths [Å], angles and torsion angles [°] of **13**

**Bond lengths**		**Bond angles / torsion angles**	
Pt1-C1	1.913(3)	C1-Pt1-N1	82.51(10)
Pt1-N1	2.084(2)	C1-Pt1-N2	81.98(10)
Pt1-N2	2.089(2)		
C4-C13	1.484(3)		
C13-O1	1.237(5)		
C1-C2	1.389(4)		
C1-C6	1.384(3)	C4-C13-C4^{i}	122.4(3)
C2-C3	1.387(4)	C4-C13-O1	118.81(16)
C3-C4	1.402(4)	Pt1-N1-C7-C2	-29.0(3)
C4-C5	1.406(4)	Pt1-N2-C10-C6	-29.2(2)

C5-C6	1.385(3)	C3-C4-C13-O1	22.9(2)
		C5-C4-C13-O1	-156.08(18)
**Interplanar angle**			
[C1-C2-C3-C4-C5-C6], [O1, C4, C13, $C4^{i}$]		23.47(13)	

5. Symmetry code *i*: -x, y, ½-z

5. Comparison of the structural features of ***5b****,* ***1*** *and* ***3****.*

Comparison of the structural features of **1,** *Z*-tamoxifen*,* vd Waals representation, with the corresponding structures of its organometallic analogues **3** and **5b** (this study), see Chart 1 in 1. Introduction of full text.

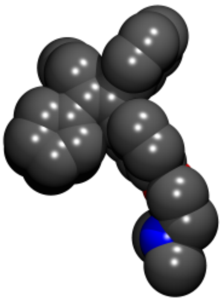

1, *Z-tamoxifen*
R=H; TTAMOX01

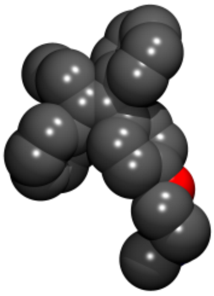

3, *Z*-ferrocefin
R=H; PUFKEG

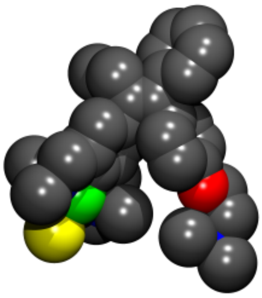

5b, *Z-pincercifen*
R=H: NUWJIB

Blue = N, red = O, Green is Pt, yellow = Cl; in **3** the CpFe moiety is covered by the Cp ring

1, TTAMOX01: Precigoux, G et al. *Acta Cryst.* **1979,** *B35*, 3070, cf. ref [8],
3, PUFKEG: Top, S. et al. *J. Organomet. Chem*. **1997,** *541*, 355 [47],
5, NUWJIB: Kooijman,et al. *CSD Communication*, **2020** [34]
